# Optimized Manufacture of Lyophilized Dermal Fibroblasts for Next-Generation Off-the-Shelf Progenitor Biological Bandages in Topical Post-Burn Regenerative Medicine

**DOI:** 10.3390/biomedicines9081072

**Published:** 2021-08-23

**Authors:** Alexis Laurent, Corinne Scaletta, Philippe Abdel-Sayed, Murielle Michetti, Marjorie Flahaut, Jeanne-Pascale Simon, Anthony de Buys Roessingh, Wassim Raffoul, Nathalie Hirt-Burri, Lee Ann Applegate

**Affiliations:** 1 Regenerative Therapy Unit, Lausanne University Hospital, University of Lausanne, CH-1066 Épalinges, Switzerland; alexis.laurent@unil.ch (A.L.); corinne.scaletta@chuv.ch (C.S.); philippe.abdel-sayed@chuv.ch (P.A.-S.); murielle.michetti@chuv.ch (M.M.); Marjorie.Flahaut@chuv.ch (M.F.); nathalie.burri@chuv.ch (N.H.-B.); 2 Manufacturing Department, TEC-PHARMA SA, CH-1038 Bercher, Switzerland; 3 Manufacturing Department, LAM Biotechnologies SA, CH-1066 Épalinges, Switzerland; 4 Unit of Legal Affairs, Lausanne University Hospital, University of Lausanne, CH-1011 Lausanne, Switzerland; Jeanne-Pascale.Simon@chuv.ch; 5 Romand Burn Center, Lausanne University Hospital, University of Lausanne, CH-1011 Lausanne, Switzerland; anthony.debuys-roessingh@chuv.ch (A.d.B.R.); wassim.raffoul@chuv.ch (W.R.); 6 Children and Adolescent Surgery Service, Lausanne University Hospital, University of Lausanne, CH-1011 Lausanne, Switzerland; 7 Plastic, Reconstructive, and Hand Surgery Service, Lausanne University Hospital, University of Lausanne, CH-1011 Lausanne, Switzerland; 8 Center for Applied Biotechnology and Molecular Medicine, University of Zurich, CH-8057 Zurich, Switzerland; 9 Oxford OSCAR Suzhou Center, Oxford University, Suzhou 215123, China

**Keywords:** cell biobanking, biotechnology, cell therapies, lyophilization, manufacturing optimization, organ donation, regenerative medicine, skin fibroblast progenitor cells, standardized transplants, wound healing

## Abstract

Cultured fibroblast progenitor cells (FPC) have been studied in Swiss translational regenerative medicine for over two decades, wherein clinical experience was gathered for safely managing burns and refractory cutaneous ulcers. Inherent FPC advantages include high robustness, optimal adaptability to industrial manufacture, and potential for effective repair stimulation of wounded tissues. Major technical bottlenecks in cell therapy development comprise sustainability, stability, and logistics of biological material sources. Herein, we report stringently optimized and up-scaled processing (i.e., cell biobanking and stabilization by lyophilization) of dermal FPCs, with the objective of addressing potential cell source sustainability and stability issues with regard to active substance manufacturing in cutaneous regenerative medicine. Firstly, multi-tiered FPC banking was optimized in terms of overall quality and efficiency by benchmarking key reagents (e.g., medium supplement source, dissociation reagent), consumables (e.g., culture vessels), and technical specifications. Therein, fetal bovine serum batch identity and culture vessel surface were confirmed, among other parameters, to largely impact harvest cell yields. Secondly, FPC stabilization by lyophilization was undertaken and shown to maintain critical functions for devitalized cells in vitro, potentially enabling high logistical gains. Overall, this study provides the technical basis for the elaboration of next-generation off-the-shelf topical regenerative medicine therapeutic products for wound healing and post-burn care.

## 1. Introduction

Major and multifactorial bottlenecks have recently been affecting and often hindering the industrial transposition and clinical translation of emerging regenerative medicine protocols and defined cell therapy products [1,2,3,4,5]. When holistically considering the development and commercialization lifecycles of such products (i.e., from conception to post-marketing surveillance), recurrently reported gridlocks include technical and technological hurdles (i.e., sustainability and stability of proposed therapeutic cell sources, respectively), as well as regulatory challenges (i.e., increasingly cumbersome requirements), impacting both public and private sponsors [6,7,8,9,10,11,12]. While numerous and various promising starting biological materials (e.g., diverse tissues, somatic or stem cell sources) and processing workflows (e.g., serial cultivation of primary cell types or cell lines, derivation of exosomes, vesicles, lysates, etc.) have been proposed and discussed, the multifactorial requirements for tangible large-scale manufacturing of therapeutic cells and cell-derived products often represent high technical complexity [13,14,15]. Therein, systematic and iterative optimization of standardized processes for biological starting material sourcing, cell isolation, culture expansion, and subsequent processing are essential for assurance of adequate (i.e., safe, effective, and consistent) therapeutic biological product availability to large numbers of patients [16,17,18,19]. The aforementioned methodological aspects represent prerequisites or technical foundations for further research in view of cell-based or cell-derived therapeutic product development, wherein selection of optimal starting and raw materials constitute cornerstones. 

Cultured primary fibroblast progenitor cells (FPC), which are normal human adherent diploid and fibroblastic cell types in vitro, have been a main focus of translational research in Swiss allogeneic regenerative medicine. Over two decades of clinical experience have outlined both safety and performance of such cell sources for enhanced therapeutic management of pediatric burn wounds, donor site wounds, and refractory geriatric ulcers in particular [20,21,22,23,24,25]. Noteworthy inherent technical and technological advantages of FPC source exploitation comprise high sustainability and robustness, excellent consistency and stability, adaptability to industrial-scale manufacture, and potential for effective repair stimulation or regeneration promotion of wounded tissues [21,22,26]. Importantly, as assessed from manufacturing and ethical standpoints, we had previously demonstrated that specifically devised primary cell biobanking strategies, integrated in validated ad hoc FPC transplantation programs, potentially enable the generation of over 39 billion therapeutic cell-based topical product doses (e.g., progenitor biological bandages, PBB) after a single qualifying organ donation [25,27]. Regulatory-wise, therapeutic products consisting of or containing cultured FPCs as active pharmaceutical ingredients (API) are considered as standardized transplants (TrSt) under applicable Swiss laws and as (combined) advanced therapy medicinal products (ATMP) in European classifications, due to the defined “substantial manipulations” incurred by and within cell banking workflows [28,29]. Quality-driven enhanced meeting of clinical needs (i.e., early coverage of burn wounds) and simplified logistical workflows (i.e., refrigerated cold chain use instead of dry ice or liquid nitrogen product shipping) are key objectives driving the development of a standardized and stabilized form of FPC-based active substance (e.g., devitalized integral cells or cell-free derivatives) for the next generation of PBBs [26,27].

Herein, we present original data on stringently optimized and up-scaled processing (i.e., multi-tiered biobanking and cell stabilization) of dermal FPC sources (i.e., FE002-SK2 cell type), with the objective to address sustainability, stability, and logistics issues around the considered standardized therapeutic biological materials. Detailed and specific goals of the present study comprised the specification of the most appropriate cell culture parameters or processes for banking and manufacture of the FE002-SK2 cell source (i.e., for maximized production yields and with minimal direct costs), as well as the in vitro validation of qualitative and functional equivalence between freshly harvested cells, corresponding cell lysates, and corresponding cell lyophilizates for cutaneous wound repair promotion. Firstly, benchmarking of appropriate manufacturing parameters, key reagents (e.g., medium supplement source, dissociation reagent), consumables (e.g., culture vessels), and technical specifications enabled global process quality and efficiency optimization, approaching validated practices in the field of industrial biotechnological substrates [30,31,32,33,34,35]. Therein, establishment of detailed processes, related controls, and applicable quality criteria for the considered manufacturing phases provided an enhanced understanding of key and critical factors implicated in large-scale dermal FPC production in GMP settings. Secondly, stabilization of banked dermal FPCs using lyophilization was undertaken, for subsequent qualitative and functional qualification of the obtained stable and devitalized FE002-SK2 cells, as compared to the same freshly harvested viable material and/or derivatives. Holistically optimized processes as described herein aim to enable immediate and sustainable provision of safe and consistent biological materials to be used as standardized transplant APIs, alternative cellular starting materials, or cell-based cell-free components in tissue engineering products or medical devices, thereby potentially diversifying therapeutic armamentariums currently available in regenerative medicine. Specifically, the work presented herein provides the technical basis for the elaboration and development of next-generation off-the-shelf topical regenerative medicine therapeutic products for wound healing and post-burn care.

## 2. Materials and Methods

### 2.1. Establishment and Qualification of Dermal FPC Sources in GMP Settings

Two skin organ donations at 14 weeks of gestation (i.e., FS20/E16 and FE002 donations) served as starting materials for establishment of the dermal FPC types (e.g., FS20/E16-Sk and FE002-SK2 cell types, respectively) used in the investigations presented herein. Both organ donations were procured in view of clinical applications in tissue engineering and were included in specific FPC transplantation programs, devised for clinical cell banking, and registered with federal health authorities since 2008. Prior to these specific donations, the federally registered Swiss FPC transplantation program was originally initiated in 1993 to study developmental cellular biology and oxidative stress. Therein, developmental stage selection for FPC isolation and culture initiation had been firstly optimized (i.e., extensive study of 29 individual skin donations, gestational ages of 13–18 weeks). Thus, to prepare adequate cellular materials for the present study, isolated cutaneous biopsies were mechanically and/or enzymatically processed for culture initiation of adherent dermal progenitor fibroblasts in GMP-compliant manufacturing suites, as described previously [26]. Briefly, procured tissue samples were thoroughly washed in conserved buffer and were either minced in conditioned tissue culture Petri dishes or were submitted to enzymatic cell isolation using trypsin-EDTA as a dissociation reagent before plating in culture dishes (Supplementary Methods). After addition of adequate cell culture medium, the culture vessels were incubated at 37 °C in humidified incubators under 5% *v*/*v* CO_2_. After regular successive medium exchange procedures, confluent preliminary cell cultures were harvested by trypsinization, and resulting cell populations were used to perform sub-cultures following defined technical specifications (Supplementary Methods). Once these sub-cultures were appropriately maintained and harvested, the resulting biological materials were cryopreserved in individual vials for establishment of dermal FPC parental cell banks (PCB) at passage 1. Thereinafter, mechanically isolated dermal FPCs were used for the cell banking and manufacturing optimization steps presented herein. After appropriate testing and qualification of PCB materials, these were used as starting materials in defined serial expansion workflows, in order to establish pilot working cell banks (WCB) at passage 5 in GMP-accredited facilities (BioReliance, Merck Group, Glasgow & Stirling, UK) and in the CHUV (Lausanne, Switzerland) (Supplementary Methods). Obtained materials were submitted to specific analyses (i.e., morphology recording in phase contrast microscopy and transmission electron microscopy, karyology study, and DNA fingerprinting), for confirmation of identity and quality. Thereafter, dermal FPC materials were used for analysis of cell surface marker (i.e., clusters of differentiation) profile by FACS analysis and for additional confirmation of cell population identity and purity by analysis of p63 nuclear marker expression (Supplementary Methods).

### 2.2. Manufacturing Optimization of Dermal FPC Types in GMP Settings

Pilot WCB vials were used in recovery procedures and expanded to confluency in adherent monolayers, before dermal FPCs were harvested and used in various assays for GMP manufacturing parameter optimization. Investigated parameters and variables comprised raw materials (i.e., fetal bovine serum manufacturer and lot number) and ancillary materials (i.e., trypsin-EDTA versus TrypLE™ dissociation reagent), as well as contact-process consumables (i.e., culture vessel surface area) and general technical specifications (i.e., cell seeding densities, culture medium volume per flask, total culture periods). Optimization of manufacturing parameters and related technical specifications were guided by cell harvest yield maximization, while retaining acceptable cell proliferation characteristics (i.e., morphology of proliferating cells, expansion kinetics) and overall quality. Based on the acquired experience and available data, appropriate key process parameters, critical process parameters, in-process controls, post-process controls, acceptance criteria, and critical quality attributes were defined and included in optimized workflows and related technical specifications for primary cell type establishment and for standardized dermal FPC culture, respectively. Thereafter, the optimized specifications were used and applied in large-scale GMP banking of FE002-SK2 cells up to passage 12 (i.e., EOPCB lots), starting from original PCB materials. Therein, multiple parameters (e.g., proliferative cell morphology, cellular viability upon initiation) were monitored and assessed by two senior operators, in addition to standard quality controls and appropriate testing (Supplementary Methods).

### 2.3. Optimization of Lyophilization Parameters for Stabilization of Dermal FPCs

In order to obtain the most adequate formulations for preservation of the therapeutic potential of the cellular components, various parameters of the lyophilization process were optimized within a personal privileged collaboration with Prof. Louis Rey and thereafter in collaboration with a specialized platform (Aérial, Centre de Ressources Technologiques, Institut Technique Agro-Industriel, Illkirch, France). Investigated and optimized parameters comprised the composition of the cryo- and lyo-protectant solution mix and the lyophilization cycle parameters. Therefore, WCB vials of FE002-SK2 dermal FPCs were used for the manufacture of pilot lyophilizate production batches. Therein, once sufficient quantities of cells were available, these were enzymatically harvested, rinsed, enumerated, and resuspended in various sterile cryo- and lyo-protective solutions designed for obtention of lyophilization cakes or for further processing in characterization experiments (Appendix A, Table A1). Final cell titers in the resulting suspensions were 10^6^ to 10^7^ total cells/mL. Corresponding placebos were prepared using bovine serum albumin (BSA) to replace the cellular components, based on equivalent total protein quantities, determined by bicinchoninic acid (BCA) assays (Supplementary Methods). Cell suspensions were then aseptically dispensed in 2R lyophilization vials, with final bulk filling volumes of 0.75 mL/vial. Vials were stoppered in open position and placed in small individual groups in LyoGuard^®^ lyophilization containers (Gore, Newark, DE, USA) or collectively in adaptiQ^®^ nests (Schott, Mainz, Germany) themselves subsequently placed in dedicated Lyoprotect^®^ lyophilization bags (Teclen, Oberpframmern, Germany) (Supplementary Methods). Resulting packaged materials were frozen in a defined process using ultra-low temperature freezers until further processing. Then, for appropriate process parameter optimization and semi-industrial production of dermal FPC lyophilizates, differential thermal analysis (i.e., cooling and heating phases) and impedance measurements allowed to establish optimal temperature, time, and vacuum settings for the lyophilization cycle, with the objective (i.e., for product development) of obtaining stable cakes and a residual relative humidity level under 2% (Supplementary Methods). Thereafter, packaged lyophilization vial lots were transferred during production on an adapted cooled stainless-steel tray and were loaded for freeze-drying following the optimized process. After the lyophilization cycle, vials were automatically fully stoppered and manually sealed using aluminum seals (Supplementary Methods). Obtained lyophilizates were labelled and stored at 4 °C until further analysis.

### 2.4. Qualitative and Functional Characterization of Lyophilized Dermal FPCs

General quality controls were firstly performed, such as visual descriptive analysis of obtained lyophilizate cakes, using predefined parameters and acceptance criteria. Based on these preliminary results, the cryo- and lyo-protective formulas (i.e., sugar or polymer-based solutions) which did not allow obtention of acceptable cakes were excluded from further investigations pertaining to product development. Then, residual humidity in preliminarily conforming preparations was measured following the Karl Fisher method (Supplementary Methods). Sample homogeneity was assessed by particle size distribution analysis using laser diffraction (Supplementary Methods). Total protein contents and protein profiles of lyophilized dermal FPCs were compared to those of freeze-thawed dermal FPC suspensions after analysis using BCA assays (Supplementary Methods). Cell devitalization was assessed by microscopic observation using Trypan blue exclusion dye, and was confirmed by in vitro cell adhesion and proliferation recovery experiments using complete growth medium (Supplementary Methods). Thereafter, for qualitative and quantitative composition determination of obtained materials, a multiplex comparative proteomic analysis was performed on lyophilized dermal FPCs and on corresponding freshly lysed cells after sample processing by a specialized platform (Eve Technologies, Calgary, AB, Canada, Supplementary Methods). Furthermore, analysis of cell surface markers after lyophilization processing allowed to assess the physical impact thereof, as compared to direct analysis of fresh cells or cells submitted to steam sterilization processing (Supplementary Methods). Then, for assessment of dermal FPC function preservation during processing by lyophilization, the stimulation potential toward primary keratinocyte proliferation, juvenile and adult fibroblast proliferation (i.e., with and without culture inserts), and HaCaT cell migration (i.e., scratch assay) were determined for several lyophilizates (Supplementary Methods). Finally, the effects of dermal FPC lyophilizates on interleukin (i.e., IL-6, IL-8) production by adult fibroblasts were evaluated in vitro in a TNF challenge assay (Supplementary Methods).

### 2.5. Statistical Analysis

For statistical comparison of average values from two sets of data, an unpaired Student’s T-test was applied, following appropriate evaluation of the normal distribution of data, wherein a *p* value < 0.05 was retained as a base for statistical significance determination. For statistical comparison of values from multiple sets of quantitative data from experiments wherein multiple variables apply (e.g., multiple groups, various treatment concentrations), a one-way ANOVA test was performed, and was followed (i.e., when appropriate) by a post hoc Tukey’s multiple comparison test or was substituted by a Kruskal-Wallis one-way analysis of variance (i.e., for non-parametric data such as gradings), wherein a *p* value < 0.05 was retained as a base for statistical significance determination. The calculations were performed using Excel (Microsoft Corporation, Redmond, WA, USA) and GraphPad Prism v. 8.0.2 (GraphPad Software, Inc., San Diego, CA, USA).

## 3. Results

### 3.1. Establishment and Qualification of Dermal FPC Sources in GMP Settings

Basic technical aspects of dermal FPC banking required specific optimization, as they play major roles on the resulting cellular material yields, quality, and related direct costs during transposition to good manufacturing practice (GMP) platforms. For applied research and eventual clinical application purposes (i.e., FPC transplantation), donated skin samples (i.e., ventral and dorsal cutaneous tissue) were made available after processing and dissection by a specialized pathologist. For maximized safety and traceability, biological starting materials were all procured through the Swiss FPC transplantation program for differential (i.e., mechanical or enzymatic tissue processing) parallel primary cell type isolation and parental cell bank (PCB) establishment (Supplementary Figures S1–S4). Specific characterization experiments confirmed the identity and quality of the cell source of interest (i.e., morphology, evolutive karyology, DNA fingerprint, cell surface marker profile, and p63 nuclear marker expression, Supplementary Figures S5–S10). Based on the various optimization and validation steps performed during starting material procurement and processing, an optimized workflow for primary FPC type establishment (i.e., including process parameters and controls, acceptance criteria, general and specific risk analyses) was elaborated (Supplementary Figure S11, Process Parameter and Risk Analysis Matrix Supplementary Documents). It is to note that the selected nomenclature for description of in vitro cell age was the passage number in the present study, instead of the cumulative cell population doubling value. This is based on the technical fact that the FE002-SK2 cells have been isolated in culture and further propagated using defined and consistent relative cell seeding densities and culture periods. Therefore, each incremental population doubling value remains consistent between passages within the qualified in vitro lifespan of the cell type. Typically, and depending on the exact production system, the mean population doubling value of the FE002-SK2 cell type is comprised within 4.85 ± 0.75 for each individual in vitro passage (i.e., ≤passage 8).

### 3.2. Optimization of Technical Specifications for Dermal FPC Industrial GMP Manufacture

**Figure 1:**
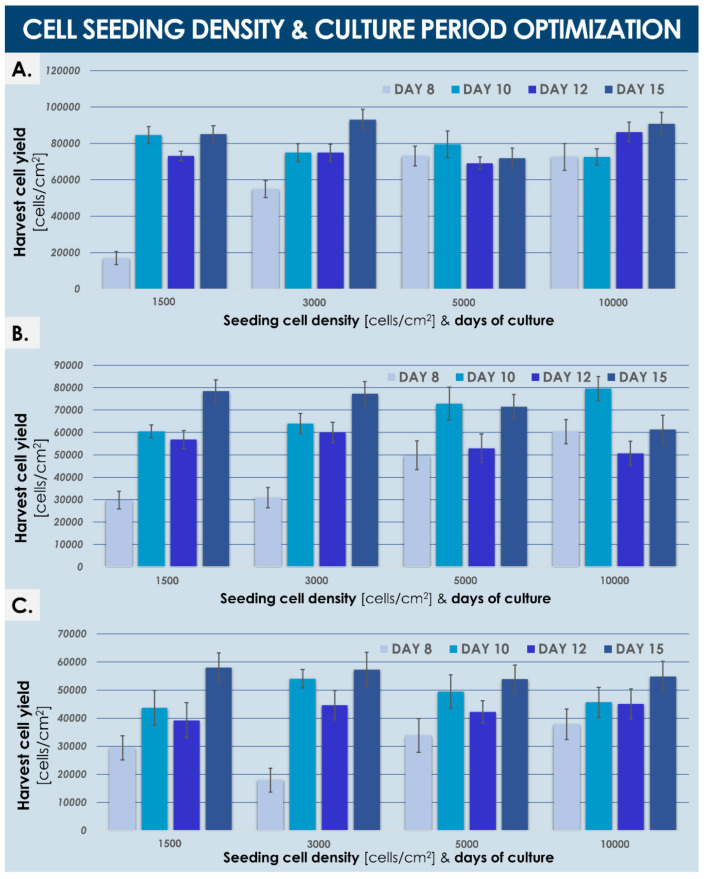
Results from the pilot GMP banking campaign for optimization of dermal FPC seeding density, culture vessel surface, and total culture period. Expansion of dermal FPCs at passage 6 took place in standard culture flasks of different surfaces (i.e., (**A–C**), T175, T225, and T500 flasks, respectively). Expansions were performed using a Sigma^®^ clinical-grade FBS lot and the relative volume of culture medium was of 0.2 mL/cm^2^. Culture medium was exchanged twice per week. The total number of days in culture was calculated from the time of vessel seeding to the time of confluent (i.e., 100% confluency) cell monolayer harvest. Standard deviations are presented as error bars. Overall optimized conditions (i.e., a compromise between sparing use of resources and consistent quality of manufactured cells) were established with the use of T225 flasks, a cell seeding density of 1.5 × 10^3^ ± 200 cells/cm^2^, and harvest of confluent cells after 15 days of culture maintenance. FBS, fetal bovine serum; FPC, fibroblast progenitor cells; GMP, good manufacturing practices.

GMP pilot cell banking campaigns enabled holistic manufacturing process optimization to be conducted using mechanically isolated dermal FPCs. This was performed in order to select the most adequate raw materials (e.g., medium supplement source), ancillary materials (e.g., dissociation reagent), contact-process consumables (e.g., culture flasks), and culture conditions (e.g., cell seeding densities, culture medium volumes, culture periods) for large-scale manufacture of dermal FPCs (e.g., FE002-SK2 cell type) (Table 1, Figure 1 and Supplementary Figures S12–S14). Optimal in vitro proliferation conditions were established in GMP settings for the FE002-SK2 dermal FPC type using a fetal bovine serum (FBS) clinical-grade lot (Sigma^®^, St. Louis, MO, USA) for cell culture medium supplementation, using 225 cm^2^ T225 cell culture flasks (Nunc™, Thermo Fisher Scientific, Waltham, MA, USA), a relative cell seeding density of 1.5 × 10^3^ ± 200 viable cells/cm^2^, relative culture medium volumes of 0.2 mL/cm^2^, culture medium exchanges twice per week, and 14–15 days of culture maintenance before harvest and filling (Table 1, results partially shown, Figure 1).

**Figure 2:**
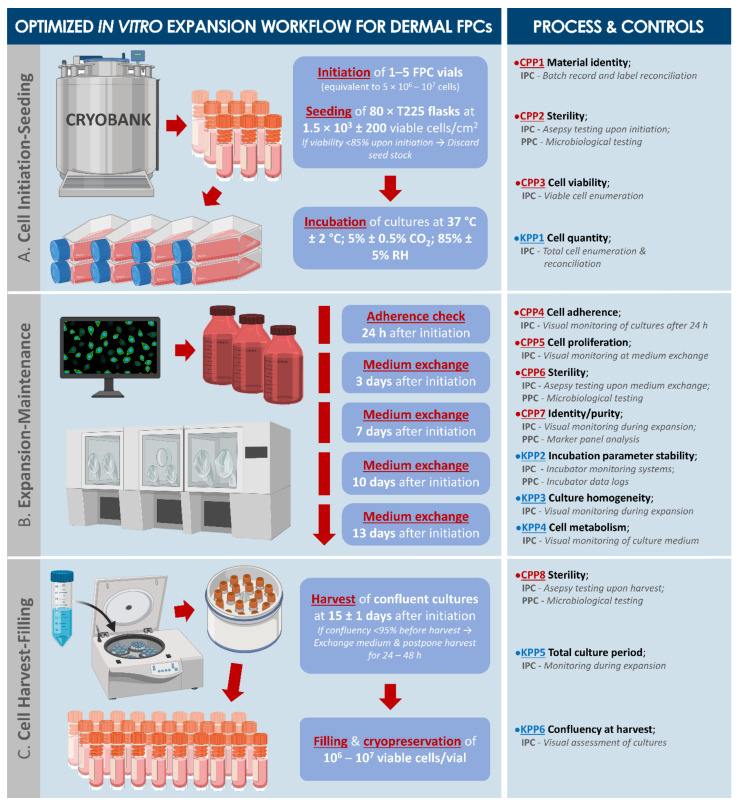
Optimized in vitro expansion workflow and ad hoc technical specifications for large-scale banking of dermal FPCs in GMP platforms (e.g., preparation of API batch from FPC WCB vials). Such technical specifications are adapted for culture of cells at passage levels relevant for clinical application. (**A**) Use of relatively low cell seeding densities (i.e., 1.5 × 10^3^ cells/cm^2^) enables the sustainable exploitation of FPC banks, with reduced enzymatic stress on the cells, incurred by harvest procedures. (**B**) Expert visual monitoring and assessment is critical in ensuring the adequate quality of the production batch and to detect potential problems (e.g., population contamination, microbial contamination, early senescence) during expansion phases. (**C**) Harvesting of fully confluent cultures (i.e., 100% confluency) enables maximization of manufacturing yields, associated with reduced direct production costs. Experimentally, systematic technical approaches enabled maximization of manufacturing efficiency for harvest of homogenous cellular materials. Complete culture medium was composed of DMEM, supplemented with 10% *v*/*v* FBS and additional L-glutamine 1%. Cryopreservation medium was composed of 50% *v*/*v* complete medium, 40% *v*/*v* FBS, and 10% *v*/*v* DMSO. CPPs were defined as parameters exerting a critical effect on the quality of the final manufactured cell batch. KPPs were defined as parameters exerting a key effect on the quality of the final manufactured cell batch. API, active pharmaceutical ingredient; CPP, critical process parameter; DMEM, Dulbecco’s modified Eagle medium; DMSO, dimethyl sulfoxide; FBS, fetal bovine serum; FPC, fibroblast progenitor cells; GMP, good manufacturing practices; IPC, in-process control; KPP, key process parameter; PPC, post-process control; RH, relative humidity.

Based on the various optimization and validation steps performed during pilot large-scale banking phases, an optimized workflow for primary FPC type culture-expansion (i.e., including process parameters and controls, acceptance criteria, general and specific risk analyses) was elaborated (Figure 2, Process Parameter and Risk Analysis Matrix Supplementary Documents).

Specifically validated processes (e.g., use of TrypLE™ dissociation reagent) were then selected, and optimal specifications were applied for multi-tiered cell banking, wherein several sub-cultures were established from the original dermal FPC PCB materials (i.e., passage 1 cells) through serial in vitro expansion in appropriate inducive culture conditions, forming master cell banks (MCB, passage 2), working cell banks (WCB, passages 3–7), and end of production cell banks (EOPCB, passage 12) (Supplementary Figures S14 and S15). Therein, the adopted cell bank tier nomenclature was specifically based on in vitro cell passage numbers, rather than on cell population doubling values (PDV), based on the fact that both initial cell seeding and endpoint culture harvest parameters were predefined and consistent throughout production passages. Eventually, during the large-scale cell banking campaigns of dermal FPCs, proliferative cell morphology, cell viability, and batch sterility (i.e., absence of extraneous contaminants) were systematically assessed throughout passages. Therein, results systematically complied with specifications and defined quality controls. Proliferative cellular morphology was observed to be consistently fibroblastic (i.e., spindle-shaped elongated cells) and characteristic for such dermal FPC types, as microscopically assessed by two senior operators specialized in FPC culture. Results of relative cellular viability determination upon cell initiation from cryopreservation (i.e., assessed by Trypan blue exclusion dye staining) were systematically ≥95% in value, thereby confirming excellent cell stability during cryopreservation in a DMSO-containing medium for time periods exceeding twelve years. Overall, the multi-tiered dermal FPC banking workflow devised for the FE002-SK2 cell type was experimentally repeated and validated at least four times (Supplementary Figure S15).

With the further sub-tiering of WCB production lots between passages 3 and 7, a highly sustainable model for dermal FPC source exploitation was adopted, with the potential to ultimately abolish the resort to repeated original organ donations. Therein, projections confirmed that up to several billion WCB vials (i.e., ≥3.9 × 10^9^ vials at passage 7) may be derived from original dermal FPC PCBs through robust cell banking workflows (Supplementary Figure S15).

### 3.3. Optimization of Dermal FPC Stabilization by Lyophilization

#### 3.3.1. Protective Formula Benchmarking and Lyophilization Cycle Optimization

Adapted criteria were defined for lyophilized FPC-based preparation obtention, which guided the optimization of the cryo- and lyo-protective mix composition and of the lyophilization cycle parameters (Table A1, Table A2 and Table A3). Specifically, several cryoprotective mix formulas (i.e., formulas K–N) were preliminarily excluded for further formulation of cell lyophilizates based on the inability to form structurally coherent lyophilization cakes after processing (Supplementary Figure S16). Formulas B, F, and I were retained for further studies, as resulting cakes were preliminarily characterized as optimal (i.e., batch uniformity, physical appearance, reconstitution, residual moisture, etc.) within the panel of proposed formulas (Table 2, Supplementary Figure S17).

Furthermore, differential thermal analysis of formulas B, F, and I indicated that products underwent subcooling at temperatures comprised between −13 °C and −10 °C, followed by crystallization and a rapid rise of product temperature at around −2 °C (Table A2). Further cooling did not reveal additional or noteworthy thermal behaviors upon cooling products down to −50 °C. Upon subsequent heating, results did not evidence any vitreous transition phenomena or recrystallization peaks. Experimental results revealed that electrical conductivity remained maximal (i.e., freezing phase) at temperatures comprised between −7 °C and −21 °C. Electrical impedance (Zsinφ) values rose rapidly during the freezing phase, to attain 1 MΩ at temperatures comprised between −15 °C and −18 °C. During the heating phase, electrical impedance (Zsinφ) values diminished progressively but rapidly, starting at −50 °C. The Zsinφ = 1 MΩ point was reached at temperatures comprised between −14 °C and −10 °C, and the impedance reached a null value at temperatures comprised between −4 °C and −2 °C, respectively. Analysis of the product sublimation process indicated that a limit temperature of −41 °C should be respected for effective lyophilization, while avoiding micro- and macro-collapse of the product.

**Figure 3:**
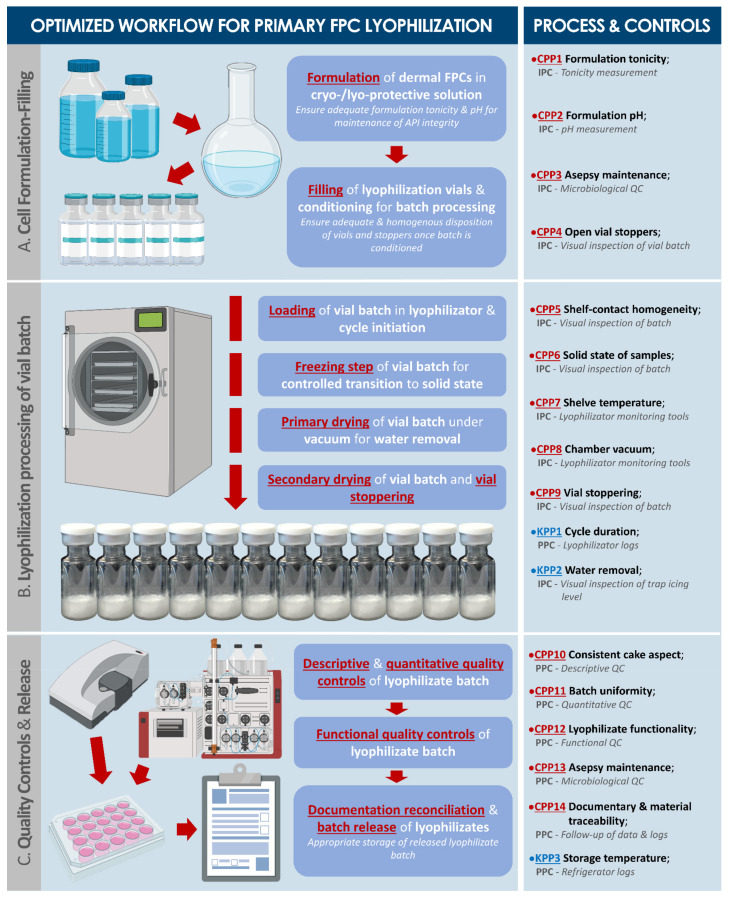
Optimized schematic workflow for primary dermal FPC stabilization by lyophilization. (**A**) Physico-chemical specifications of unprocessed bulk and of lyophilized products (e.g., final tonicity and pH after reconstitution), defined during the development phase, are critical in maintaining the functionality of processed biological materials and must comply with general requirements of the considered administration route. (**B**) Adequate primary and secondary drying phases are critical for appropriate stabilization of the considered formulations, by consistently obtaining low residual relative water contents while maintaining cake structural integrity. (**C**) Definition of controls and related variables for final lyophilized products are highly formulation-dependent and are of critical importance for assurance of batch-to-batch homogeneity. CPPs were defined as parameters exerting a critical effect on the quality of the final lyophilizate batch. KPPs were defined as parameters exerting a key effect on the quality of the final lyophilizate batch. API, active pharmaceutical ingredient; CPP, critical process parameter; FPC, fibroblast progenitor cells; IPC, in-process control; KPP, key process parameter; PPC, post-process control; QC, quality control.

Based on the above-mentioned results of electrical and thermal behavior analysis, an optimized lyophilization cycle was devised, integrating the limit product temperatures to be maintained during freezing and sublimation phases in order to attain successful lyophilization without product collapse (Table A3, Appendix A). These optimized parameters were applied for all subsequent stabilization processing of biological materials. Based on the various optimization and validation steps performed during dermal FPC processing by lyophilization and stabilized product characterization, an ad hoc optimized workflow (i.e., including process parameters, controls, and acceptance criteria) was elaborated (Figure 3, Process Parameter and Risk Analysis Matrix Supplementary Documents).

#### 3.3.2. Qualitative and Quantitative Study of Lyophilized Dermal FPCs

**Figure 4:**
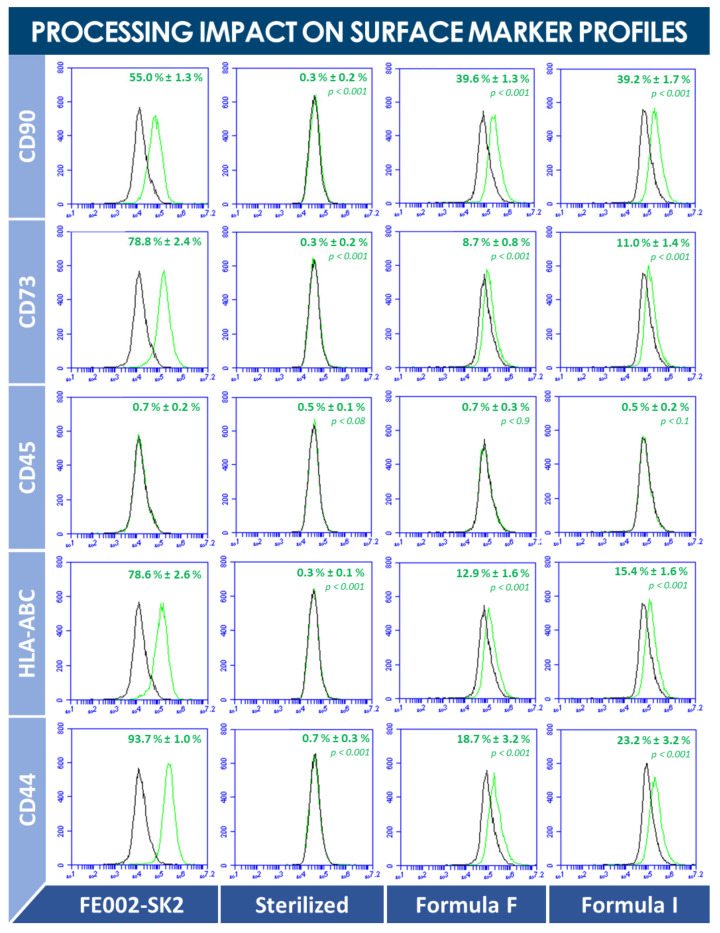
Comparative surface marker study results of dermal FPCs (i.e., viable cells, sterilized cells, and lyophilized cells) using FACS analysis, following EP method 2.7.24. “*Flow cytometry*”. Comparative analysis shows the effects of processing on the detection of cell surface markers. The mean percentages of detected labelled cells are specified on each plot, along with corresponding standard deviations. Statistically significant differences between quantitative data obtained for sterilized or lyophilized cells and the control group (i.e., freshly harvested cells) are indicated by *p* values < 0.05. Control curves are presented in black on each plot. Results indicated that while sterilization renders the detection of surface markers impossible, lyophilization of FPCs allows for qualitatively conserved specific detection thereof. Comparable quantitative data from formulas F and I were not found to be statistically different for each considered surface marker, respectively, indicating that the effect of lyophilization on cell surface markers was the same for both formulas. CD, cluster of differentiation; EP, European pharmacopoeia; FACS, fluorescence-activated cell sorting; FPC, fibroblast progenitor cells; HLA, human leukocyte antigen.

Following production of several batches of lyophilized dermal FPCs using the optimized lyophilization cycle parameters (i.e., presented in Table A3, Appendix A), qualitative and quantitative compositions of obtained preparations were investigated, in order to evaluate the effects of processing on protein contents or detection thereof. Therein, lyophilized batches (i.e., formula M, FE002-SK2 cells suspended in PBS alone, for avoidance of lyophilization mix interference) were analyzed for total protein contents (i.e., BCA assay), in view of the assessment of batch-to-batch uniformity, and by comparative proteomic analysis (i.e., in parallel to fresh cell lysates), to determine the effects of lyophilization processing on the cells of interest at a molecular level (Supplementary Methods). Relatively abundant proteins (i.e., ≥1 mg/mL) or proteins of interest for cutaneous wound healing, detected in one or in both groups (i.e., FE002-SK2 dermal FPCs, processed into fresh cell lysates, and corresponding cell lyophilizates), comprised follistatin, FGF-2, VEGF-A, HGF, TIMP-1, TIMP-2, MMP-1, MMP-2, MMP-3, MMP-13, sgp130, sVEGFR-2, sEGFR, sVEGFR-1, sVEGFR-3, sTNFRI, sIL-1RII, leptin, HB-EGF, IL-8, and TGF-β1 (Supplementary Table S1). Significant modifications (i.e., ≥1 log variation) in detected protein levels were evidenced for follistatin, FGF-2, MMP-3, MMP-13, sgp130, sVEGFR-2, sVEGFR-1, sVEGFR-3, sIL-1RII, leptin, HB-EGF, GRO-a, fractalkine, and TGF-β1, wherein detected protein quantities were systematically relatively more important in the lyophilized cell group (Supplementary Table S1). More generally, for the majority of detected proteins (i.e., detected levels above the individual limits of detection), relative levels were determined to be more important in the lyophilized dermal FPC group (Supplementary Table S1). Finally, comparative FACS analysis of fresh, sterilized, and lyophilized dermal FPCs allowed to outline complete destruction of specified surface markers during sterilization, but relative, partial, and specific conservation thereof following lyophilization (Figure 4).

#### 3.3.3. Functional Quality Control of Lyophilized Dermal FPCs

**Figure 5:**
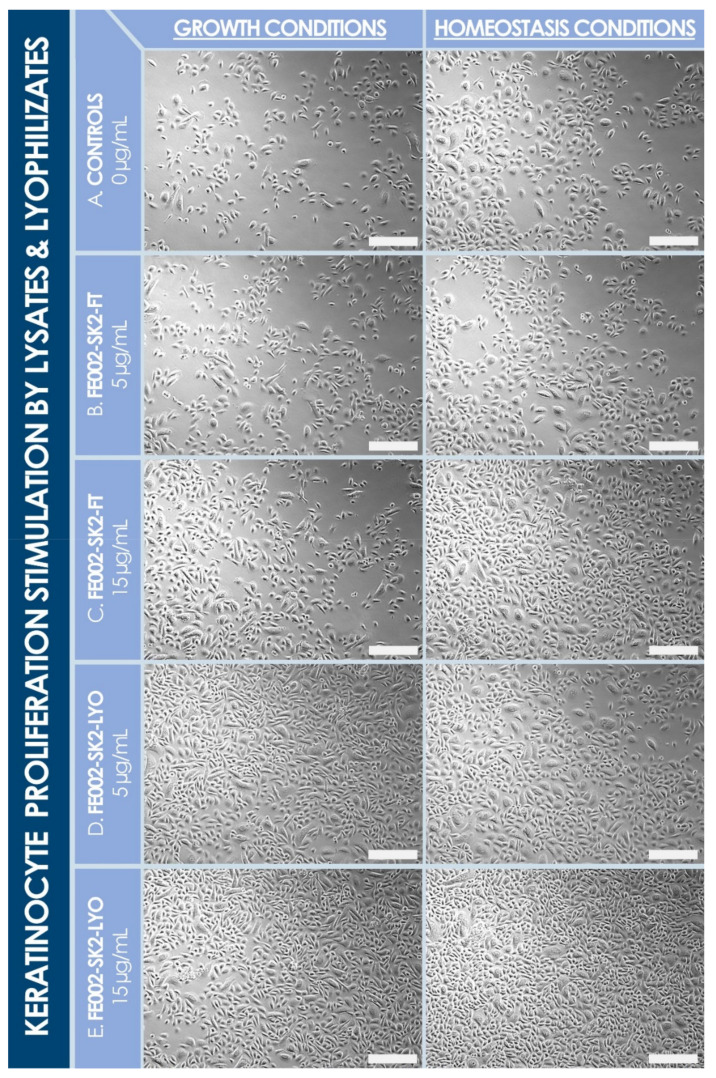
Primary keratinocyte proliferation stimulation potential of various doses (i.e., expressed in normalized total protein contents at the final dilution in the culture medium) of dermal FPC (e.g., FE002-SK2 cell type) lysates (i.e., “FT” for freeze-thaw) and lyophilizates (i.e., “LYO”, formula B), respectively. Photographic imaging was performed in both culture conditions (i.e., growth and homeostasis conditions) after an incubation period of 96 h. (**A**) Control group. (**B**) Lysate group at a concentration of 5 µg/mL. (**C**) Lysate group at a concentration of 15 µg/mL. (**D**) Lyophilizate group at a concentration of 5 µg/mL. (**E**) Lyophilizate group at a concentration of 15 µg/mL. Visual assessment indicates a positive effect of lyophilizates on keratinocyte proliferation at investigated doses, as compared to cell lysates and controls, respectively. Scale bars = 150 µm. FPC, fibroblast progenitor cells; FT, freeze-thaw; LYO, lyophilizate.

**Figure 6:**
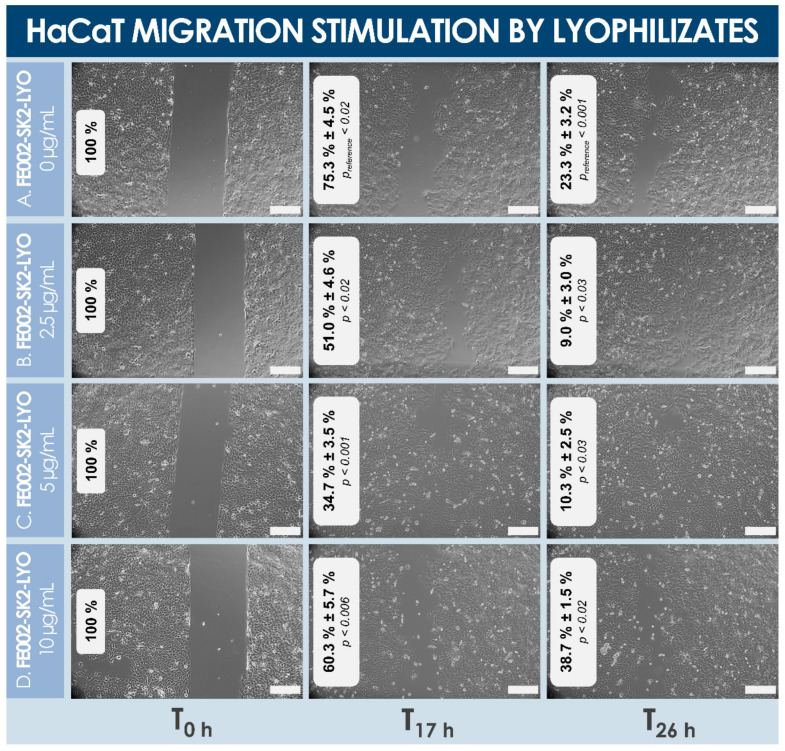
HaCaT cell migration stimulation potential of various doses (i.e., expressed in normalized total protein contents at the final dilution in the culture medium) of dermal FPC (e.g., FE002-SK2 cell type) lyophilizates (i.e., “LYO”, formula M). The culture medium did not contain FBS. Photographic imaging was performed at the time of treatment with the test-items, 17 h later, and 26 h later, respectively. Unpopulated culture surface areas were integrated using ImageJ. (**A**) Control group. Significant differences (i.e., *p_reference_* values < 0.05) were found in the remaining relative unpopulated surfaces at T_17 h_ and T_26 h_ as compared to T_0 h_, respectively. (**B**) Lyophilizate group at a concentration of 2.5 µg/mL. (**C**) Lyophilizate group at a concentration of 5 µg/mL. (**D**) Lyophilizate group at a concentration of 10 µg/mL. Quantitative assessment of remaining relative unpopulated culture surfaces indicated a positive effect of lyophilizates on keratinocyte migration at two investigated doses (i.e., 2.5 and 5 µg/mL), as compared to controls. Higher doses (i.e., 10 µg/mL) of lyophilizate promoted cell migration at T_17 h_, as compared to controls, but hindered cell migration at the T_26 h_ timepoint, as compared to controls. At both timepoints and at all treatment concentrations, statistically significative differences (i.e., *p* < 0.05) were found for remaining unpopulated surface values, as compared to corresponding values from the control group (i.e., no treatment). Scale bars = 200 µm. FBS, fetal bovine serum; FPC, fibroblast progenitor cells.

The in vitro stimulatory effects of lyophilized dermal FPCs (e.g., FE002-SK2 cells) were determined, in view of assessing the function of such devitalized and stabilized materials, as compared to corresponding dermal FPC lysates. Specifically, cell lysates and reconstituted lyophilizates (i.e., formula B) were shown to similarly stimulate cellular proliferation of primary keratinocytes when added in final concentrations of 5 µg/mL and 15 µg/mL (i.e., expressed in normalized total protein contents) in the cell culture media (Figure 5, Table 3). Therein, stimulation of cell proliferation was highly comparable in terms of amplitude, considering average cell enumeration data for both assay groups (i.e., cell lysates versus lyophilizates) and in both culture media (i.e., homeostasis versus proliferation medium, Table 3). Conversely, the effects of both cell lyophilizate concentrations (i.e., Figure 5D,E) were photographically recorded as relatively more important than that of corresponding cell lysate concentrations (i.e., Figure 5B,C). Furthermore, cell lyophilizates (i.e., formula M) were found to promote migration of HaCaT cells in a dose-dependent manner in a standardized scratch assay (Figure 6).

Specifically, concentrations of 2.5 and 5 µg/mL of lyophilizate optimally promoted cell migration in the experimental assay (i.e., >90% gap closure at the 26 h timepoint, Figure 6B,C), whereas a concentration of 10 µg/mL produced a less potent effect on gap closure than saline controls (i.e., ~60% gap closure at the 26 h timepoint, Figure 6A,D). Therefore, based on the obtained in vitro results of dermal FPC lysate and lyophilizate effects, said effects were determined to be comparable across groups, and to be dose-dependent, with an apparent loss of function at relatively higher doses (Figure 5 and Figure 6, Table 3).

**Figure 7:**
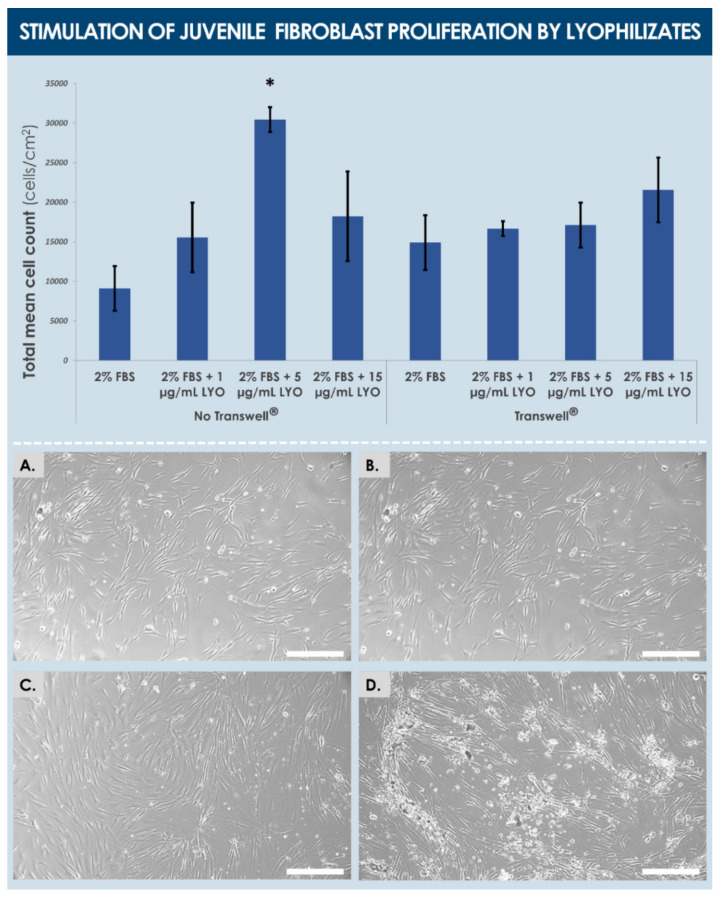
Primary juvenile fibroblast cell proliferation stimulation potential of various doses (i.e., expressed in normalized total protein contents at the final dilution in the culture medium) of dermal FPC (e.g., FE002-SK2 cell type) lyophilizates (i.e., “LYO”, formula M). The culture medium contained 2% *v*/*v* FBS. Photographic imaging was performed 48 h following the second treatment with the test-items. (**A**) Control group, no Transwell^®^ insert. (**B**) Lyophilizate group at a concentration of 1 µg/mL, no Transwell^®^ insert. (**C**) Lyophilizate group at a concentration of 5 µg/mL, no Transwell^®^ insert. Note the statistically significant difference in endpoint cell counts in this condition (i.e., *p* < 0.03), as indicated by the “*” symbol. (**D**) Lyophilizate group at a concentration of 15 µg/mL, no Transwell^®^ insert. Note the presence of lyophilizate particles adhering to the proliferating cells in this condition. Overall, obtained results suggested that direct cell contact is necessary for the observation of a positive effect of lyophilizates on fibroblast proliferation, but that high product concentrations do not allow observation of this effect because of strong adhesion of product particles to assay cells in vitro. Cell count values are presented as averages, with standard deviations as error bars. Scale bars = 100 µm. FBS, fetal bovine serum; FPC, fibroblast progenitor cells; LYO, lyophilizate.

Experimental results of fibroblast proliferation stimulation by dermal FPC lyophilizates (i.e., formula M, without cryo-protectants) showed that said effects depended on the presence of culture inserts, wherein juvenile fibroblasts were significantly promoted in their growth by a dose of 5 µg/mL of lyophilizate in the absence of a Transwell^®^ membrane (Figure 7).

**Figure 8:**
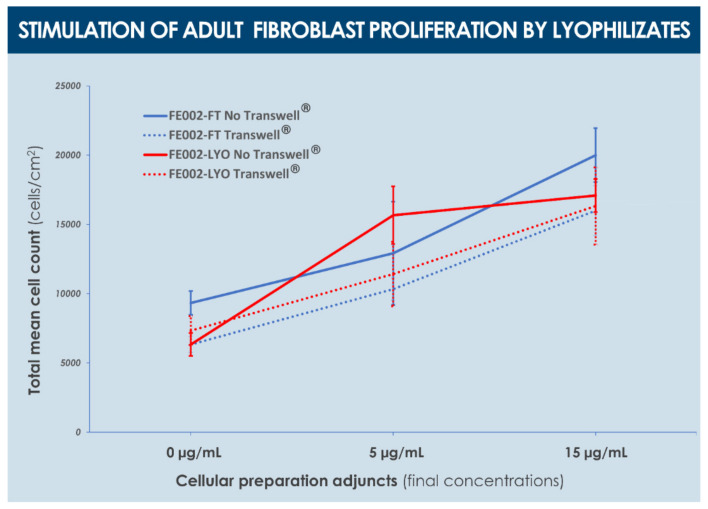
Adult primary fibroblast cell proliferation stimulation potential of various doses (i.e., expressed in normalized total protein contents at the final dilution in the culture medium) of dermal FPC (e.g., FE002-SK2 cell type) lysates (i.e., “FT” for freeze-thaw) and lyophilizates (i.e., “LYO”, formula I), respectively. The culture medium contained 10% *v*/*v* FBS. Cell enumeration was performed 96 h following treatment with the test-items. No statistically significant differences were found across the different groups, for each specific timepoint. FBS, fetal bovine serum; FPC, fibroblast progenitor cells; FT, freeze-thaw; LYO, lyophilizate.

This result suggested that direct contact between the assay cultures and the products was necessary in order for the effect to be measurable. However, higher lyophilizate doses or the presence of a Transwell^®^ membrane did not allow us to conclude on significantly increased fibroblast proliferation, suggesting here again that the stimulatory effects disappear at relatively higher doses, and that the effects exerted by soluble factors alone do not suffice to produce an observable juvenile fibroblast proliferation stimulation (Figure 7). In contrast, the proliferation of adult fibroblasts was significantly and similarly promoted, in a dose-dependent manner and independently from the presence of a culture insert, by the treatment with cell lysates and alternative dermal FPC lyophilizates (i.e., formula I, with cryo-protectants, Figure 8).

**Figure 9:**
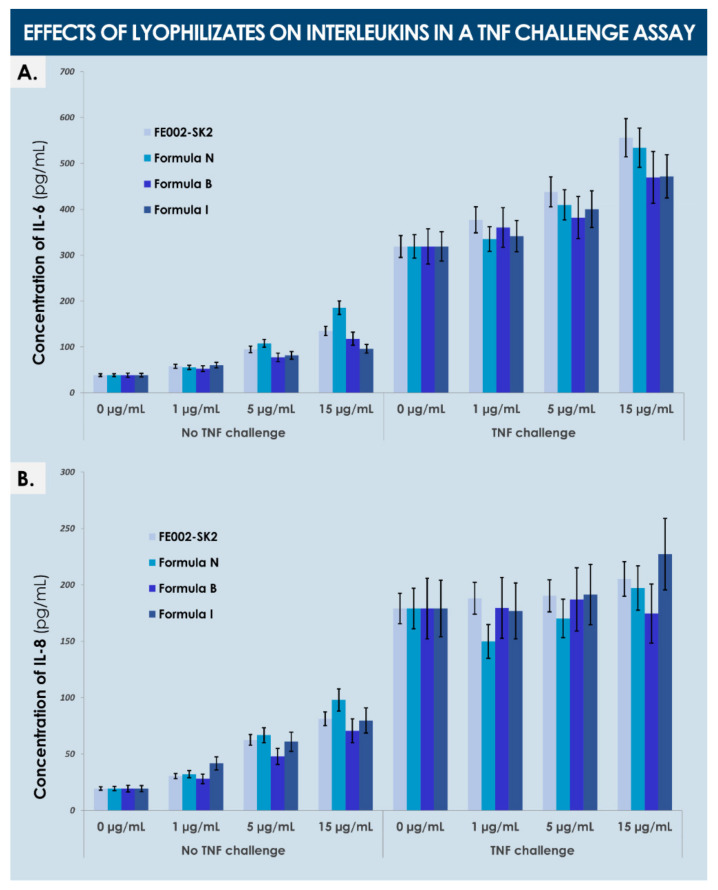
Effects of dermal FPC lyophilizates on interleukins in a TNF challenge assay. Adult fibroblasts were expanded, treated with various doses and formulations of lyophilized dermal FPCs (i.e., using fresh FE002-SK2 lysate as a control), and stimulated for 24 h with 10 ng/mL TNF-α. Thereafter, the levels of IL-6 and IL-8 were quantified in cell culture supernatants by ELISA. (**A**) Comparative results of IL-6 quantification for both assay conditions (i.e., with or without TNF stimulation). (**B**) Comparative results of IL-8 quantification for both assay conditions (i.e., with or without TNF stimulation). ELISA, enzyme-linked immunosorbent assay; FPC, fibroblast progenitor cells; TNF, tumor necrosis factor.

Although non-significant, a trend was observed in the assay wells without a culture insert, wherein cell counts were systematically determined to be higher than in corresponding wells with an insert (Figure 8). Finally, analysis of interleukin production by adult fibroblasts after treatment by dermal FPC preparations (i.e., formulas B, I, and N) in a TNF challenge assay showed an increased production (i.e., dose-dependent) of both IL-6 and IL-8 after treatment by lyophilizates in the control groups, an increased production (i.e., dose-dependent) of IL-6 following treatment with various lyophilizates in the TNF group, but no increase of IL-8 levels in the same treatment settings (Figure 9).

## 4. Discussion

### 4.1. Optimized Dermal FPC Multi-Tiered Cell Banking for Regenerative Medicine Products

In the present work, we report stringently optimized starting biological material sourcing and related multi-tiered biobanking of dermal FPC sources, with specific focus set on manufacturing process definition (i.e., parameters, controls, and ad hoc criteria, Figure 2 and Figure 3).

The objective was therein to address the quality and sustainability (i.e., original sourcing, cell bank exploitation, and manufacturing) of biological starting material sources included in the manufacture of therapeutic standardized transplant products, such as cell therapies used for treating burn victims [36]. Therein, we experimentally confirmed that a single organ donation is sufficient for the sustainable provision of adequate starting materials (i.e., through a defined cell bank system) for several decades of scientific and clinical research, as well as product development. Therefore, through a process-oriented and risk-based approach of optimized workflow elaboration, we showed that dermal FPCs may eventually be obtained in the form of high quality, safe, and effective biological APIs.

It is to note that specific FPCs are relatively simple to maintain and manufacture, as compared to various types of stem cells, extensively studied for potential therapeutic uses. Indeed, homogeneity and consistency of cultured therapeutic FPCs may be optimally demonstrated, with the definition of clear and relevant control parameters and acceptance criteria, in view of overall risk mitigation and quality maximization in regulatory dossiers. Furthermore, for sound development of manufacturing workflows at industrial scales for cell therapy product registration and eventual commercialization, many aspects such as efficiency and incurred direct costs of cell banking represent key factors [25]. Therein, validated basic parameters such as those investigated herein for technical specification optimization are prerequisites for sound technology transposition and upscaling in GMP manufacture (Table 1, Figure 1 and Supplementary Figure S16). For each newly considered cell type, proper benchmarking of relevant materials and parameters should always be considered during or following qualification and release of FPC PCB vial lots, but in any case, before initiation of full-scale banking campaigns.

From a manufacturing point of view, conjugation of primary FPC sourcing with stringently devised multi-tiered biobanking as presented herein is partially based on the applied research of Hayflick et al. from the 1960s, wherein many of the diploid cell types subsequently qualified as vaccine production substrates were established [33,34]. Therein, several ethical aspects (i.e., absence of donor consent or consent retroactively contested), as well as stability and availability issues have emerged around historically and widely used cell types (e.g., MRC-5 and WI-38 fetal lung fibroblasts). Therefore, specific legal frameworks such as FPC transplantation programs have additionally been devised in Switzerland by our group since the 1990s, for optimization of anonymous yet exhaustive traceability of starting materials and safety of progeny cells [36]. Therein, dermal FPCs (e.g., FE002-SK2 cell type) have been shown to be optimally adapted for use in homologous allogeneic regenerative medicine, in particular for managing extensive pediatric burns and refractory geriatric ulcers, for which we have gathered over two decades of safe clinical experience [20,23,36]. From a technical and manufacturing standpoint, such primary diploid human cell types are characterized as optimally stable, consistent, and sustainable.

As the original organ donation from the FE002 donor occurred at 14 weeks of gestation, the derived cellular materials are considered as pre-immunocompetent. Therefore, the probability of eliciting an immune response in recipients of these cells is quite low and such a response has never been observed in our two decades of clinical work [36]. Even after multiple and repeated applications of viable cellular products in our burn center on patients with moderate to severe burns, no specific adverse host reaction has been observed, in pediatric patients in particular [24,36]. We have also gathered extensive clinical experience with these progenitor cells in treating chronic lower-limb ulcers in geriatric patients and have not observed any specific type of adverse host reaction [23]. Therefore, based on the specific inherent characteristics of these fibroblasts, as well as our extensive clinical hindsight in cutaneous regenerative medicine, we can safely assess that the considered cell types do not pose a major threat or require special care with regard to adverse host reactions [26]. These various aspects confer tangible advantages to appropriately processed FPCs over alternative therapeutic biological material sources (e.g., various pooled sources of stem cells and/or related extracellular vesicles), considered in regenerative medicine for degenerative and inflammatory affections [37,38].

Regulatory-wise, the most straightforward pathway and classification processes for cultured FPCs consist in the development of a biological medicinal product or tissue engineering product (TEP) [27]. However, due to the high complexity of such registration, and due to the fact that therapeutic cell viability is not necessarily a criterion for efficacy or function, alternative regulatory pathways may be considered, such as in the field of medical devices. Therein, the borderline characteristic which will determine the validity of such classification and applicability of medical device regulation resides in the definition of the primary/principal and ancillary mechanisms of action of the product [39]. Specifically, while the primary mechanism of action of medical devices may not be classified as pharmacological, immunological, or metabolic in nature in Europe, an ancillary mechanism of action may be of this nature, complementing the primary mechanism of action in the obtention of the claimed therapeutic effect (i.e., exertion of the principal intended action on the body, which is necessary and sufficient) [39]. Therefore, fine interpretation of specific regulations necessarily constitutes the basis of the product design, indication definition, and regulatory classification steps, in order to rationalize the risks, costs, and length of product development.

Notably, with reference to the data presented herein on the activity of dermal FPC lysates and lyophilizates, the stimulation of cellular proliferation and migration are assimilated to metabolic effects. Therefore, the use of such materials in a medical device is excluded, unless the primary/principal mechanism of action is appropriately determined to be non-pharmacological, non-immunological, and non-metabolic, and that a potential metabolic mechanism of action of the biological material is determined to be ancillary. Such demonstrations require, in all probability, the appropriate conjugation of FPC-based materials to vehicles or scaffolds, and appropriate modulation of relative material doses. Overall and apart from the regulatory considerations of product development, it remains of high interest to include cells or cell derivatives in cutaneous repair promotion products, as the outcomes of available standard bandages and devices remain unsatisfactory in many cases. Although several types of natural (e.g., plant-based) or synthetic matrices (e.g., polymeric scaffolds, hydrogels) and components may be included in products to obtain superior pain relief activity, wound closure promotion, and regenerative process enhancement, a biological or cellular component and appropriate stimuli are required for optimal deployment of the regenerative triad in cutaneous affections [25].

### 4.2. Benchmarking of Materials and Consumables for Efficient Large-Scale Primary FPC Manufacturing

The results presented herein for the devising of an optimized FPC culture manufacturing workflow have shown that each parameter may bare significant impacts on the harvested cell batch, mainly in terms of harvest cell yield in the case of dermal FPCs. Therein, the accumulation of sub-optimal individual technical specifications may result in drastically elevated direct costs of manufacture. Benchmarking of culture vessel surfaces, cell seeding densities, and total culture periods has shown that optimal results may be obtained in culturing the considered dermal FPCs in T225 flasks, with relative cell seeding densities of 1.5 × 10^3^ cells/cm^2^ for 15 days (Figure 1). Therein, analysis of comparative data revealed a trend toward reduction of endpoint harvest cell yields with the use of increasingly large individual culture surfaces, all other parameters remaining equal (Figure 1). Such a “negative upscaling factor” was already observed by our group for alternative primary FPC types in similar cell culture systems (data not shown) and might be explained by specific gaseous exchanges or distribution in the different vessels. Therefore, T225 vessels appeared as an optimal compromise between maximized manufacturing yields and minimized manutention for culture maintenance (Table 1, Figure 1). Furthermore, use of relatively low cell seeding densities (i.e., 1.5 × 10^3^ cells/cm^2^) and slightly longer culture periods (i.e., culture maintenance for 15 days) appeared to be an optimal compromise between sparing use of biological starting materials and manufacturing suite use (Figure 1). Indeed, while the use of relatively more important cell seeding densities (i.e., 3 × 10^3^ to 10^4^ cells/cm^2^) allows rapid (i.e., after 8 to 10 days of culture) obtention of important numbers of cells (i.e., close to maximal cell yields in individually considered systems), the sparing use of cell seeds outweighs the additional costs incurred by the extended culture periods (Figure 1).

Additional parameters submitted to benchmarking were the source of FBS and the relative volume of culture medium used in cell culture systems (Table 1). Therein, high variability was evidenced between different FBS manufacturers and between different lots from the same manufacturer (Table 1, Supplementary Figure S12). This aspect is especially important to consider with the use of clinical-grade FBS, wherein the specific raw material processing may incur high variability in endpoint cell yields, prompting the need for repeated batch qualification phases during large manufacturing campaigns (i.e., if FBS lots of sufficient size are not available). Additionally, as it represents a main source of potential extraneous agent contamination, the included FBS lot must be carefully selected, notably with regard to origin and processing methods. Although there is a regulatory guidance which would designate elimination of animal-based materials altogether, technical and biological experience would claim that such changes should be performed with extreme caution and extensive validation. Furthermore, it was shown that a minimal culture medium volume was sufficient (i.e., 0.15–0.20 mL/cm^2^) (Table 1). Finally, the validation of dissociation reagent equivalence between trypsin-EDTA and TrypLE™ did not evidence statistically significative differences in proliferative or endpoint cell counts (Supplementary Figure S13).

With regard to the sustainability of considered cell types within regenerative medicine product development, the biological materials used in production are consistently comprised in the first two-thirds of the characterized and qualified in vitro lifespan of the cell source. Notwithstanding this restriction, the exponential model of multi-tiered banking of dermal FPCs allows for sufficient production of WCB materials (e.g., >3 × 10^9^ WCB_T5_ vials of FE002-SK2 cells, passage 7, Supplementary Figures S14 and S15). Specifically, the obtainable quantities of therapeutic materials outnumber necessary quantities in active substance (i.e., coverage of the entire expected product lifetime). Furthermore, the need for repeated organ donations is negated by the extensive potential material yield of a single source, thereby ameliorating bulk biological material and end-product homogeneity and safety.

### 4.3. Lyophilization as an Effective Stabilization Method for Therapeutic FPC Biological Materials

Lyophilization or sublimation have historically been proven to be effective techniques for the long-term storage of sensitive biological products (e.g., antibiotics, blood derivatives, vaccines, natural extracts, and proteins) or for logistical optimization (e.g., lyophilized food in the space industry) [40]. In the context of regenerative medicine, such processing protocols have been thoroughly investigated in the past as alternatives to standard cryopreservation of cells and tissues in liquid nitrogen, due to potentially large logistical advantages and drastically reduced storage costs [41,42,43,44,45]. Specifically, excellent results were obtained with various tissues and cell sources processed by lyophilization for subsequent management of cutaneous wounds (e.g., burns and ulcers) [46,47]. Pioneers in the domain of tissue banking and innovation in the field of tissue preservation have notably comprised the US Navy Tissue Bank scientists and Prof. Louis Rey, to cite only a few [47,48,49]. Through careful optimization of product formulation, primary container choice, and freeze-drying process parameters, it has been shown that equivalent quality and functionality may be attained between cryopreserved and lyophilized cellular APIs [50,51,52].

For the present study, in view of additional standardization of dermal FPCs as biological APIs, protocols for stabilization of cultured cells using lyophilization were optimized, with the secondary objective of simplifying logistical workflows and shortening availability delays for off-the-shelf cell therapy products or medical devices. Therein, specific process parameters, controls, and criteria were defined and adopted for the manufacture of dermal FPC lyophilizates (Figure 3, Table 2). Various formulations of cryo- and lyo-protectant solutions were investigated, with the overall goal of obtaining stable, consistent, and effective forms of lyophilized cellular preparations. This was achieved satisfactorily for cells initially suspended in formula I (i.e., saccharose, dextran, DMSO, water, Table A1, Appendix A, Supplementary Figure S17), based on the parameters and controls presented in Table 2. In addition, electrical and thermal behaviours were characterized for the three formulas presented in Table 2, which allowed to define an optimal lyophilization cycle, ensuring adequate formation of a lyophilization cake at macroscopic and microscopic scales (Table A3, Appendix A).

It is noteworthy that several investigated formulas for cell lyophilization were not designed to form structurally coherent or acceptable cakes, yet these were required for the in vitro characterization of lyophilized dermal FPCs, to exclude excipient-related artefacts (Table A1, Appendix A). Presented data on the keratinocyte proliferation stimulation potential of cell lyophilizates versus cell lysates indicated a non-significative difference in endpoint readouts in both groups and culture conditions, which suggests an excellent preservation of dermal FPC function with regard to keratinocyte cell proliferation potential following stabilization (Table 3, Figure 5). This attribute represents a highly desirable characteristic of therapeutic products aiming for restoration of the cutaneous barrier (i.e., notably via re-epithelialization), which necessitates keratinocyte proliferation and migration. Furthermore, lyophilizates were experimentally shown to stimulate keratinocyte migration in vitro, in a scratch assay modeling wound healing (Figure 6). Therein, while low doses of lyophilizate (i.e., 2.5 and 5 µg/mL) resulted in relatively smaller gap areas than controls at both timepoints (i.e., 17 h and 26 h), the higher dose of 10 µg/mL promoted cell migration at the 17 h timepoint but hindered migration at the 26 h timepoint, as compared to controls (Figure 6). Therefore, it can be assessed that the keratinocyte migration stimulatory potential of lyophilized dermal FPCs exists but is dose-dependent.

With regard to juvenile and adult fibroblast proliferation stimulation by dermal FPC lyophilizates, the influence of the cryo-protective formula was evidenced by the observed difference of effects, which might be partly due to differences in target cell properties as well (Figure 7 and Figure 8). Therein, it is to note that practically, the presence of culture inserts is necessary to avoid strong adhesion of lyophilizate particles on proliferating cells in vitro, in order to avoid proliferation hindrance by said particles. Furthermore, a non-significant trend was observed in the assay wells without an insert, wherein cell counts were systematically more important in value than in corresponding wells with an insert (Figure 8). Overall, obtained results suggested that direct cell contact is necessary for the observation of a positive effect of lyophilizates on fibroblast proliferation, but that high product concentrations do not allow observation of this effect because of strong adhesion of product particles. In fine, it can be overall concluded that, based on the presented in vitro data, both investigated functional characteristics (i.e., cell proliferation and migration stimulation potentials) of dermal FPCs appear to be conserved in the appropriate lyophilized form (Figure 5, Figure 6, Figure 7 and Figure 8).

With regard to the qualitative and quantitative comparative analyses of dermal FPC lysates and lyophilizates, results outlined that the physical process of lyophilization maintained the structural integrity of investigated proteins and cell surface markers, as these were detectable in the cell lysate (i.e., proteins) or cell suspension (i.e., surface markers) and in the corresponding lyophilizates (i.e., proteins and surface markers), respectively (Supplementary Table S1, Figure 4). Therein, specific detected protein levels were significatively increased by the lyophilization process (Supplementary Table S1). However, this aspect is in all probability linked to differences in structural modifications incurred either during initial freezing, water sublimation, and eventual sample rehydration before analysis (i.e., for lyophilization) or during the freeze-thaw cycles (i.e., for lysate production). Therefore, observed differences were most probably due to the effect of the differential processing on the availability for detection of the target proteins in the biological material complex submitted to analysis. However, it had already been previously reported that growth factor concentrations (e.g., bFGF, VEGF) were relatively higher in lyophilized preparations than in corresponding cryopreserved preparations of co-cultured fibroblasts and keratinocytes [46].

Investigation into the protein composition of the cell lysates and lyophilizates revealed the abundant presence of molecules of interest for wound healing (i.e., proteins or factors naturally implicated in physiological cutaneous development and healing, Supplementary Table S1). Among these relatively abundant proteins, follistatin appears to play a key role in the development of the skin and during wound healing in general [53]. Basic FGF (bFGF) stimulates cell proliferation and may play a role in vivo in the modulation of wound healing and tissue repair [54]. VEGF-A is a potent mediator of both angiogenesis and vasculogenesis in the fetus and in adults [55]. HGF can accelerate re-epithelialization in cutaneous wound healing [56]. Leptin knock-out mice have presented delayed wound healing, and wound closure was markedly impaired in keratinocyte-specific HB-EGF-deficient mice [57,58]. The pro-inflammatory cytokine IL-8 (CXCL8) promotes neutrophil adhesion to the vascular endothelium and migration to sites of inflammation [59]. TGF-β1 is important in inflammation, angiogenesis, granulation tissue formation regulation, extracellular matrix remodeling, and is essential for re-epithelialization [60,61,62,63,64]. TIMP-1 acts as a cell growth factor (i.e., for both keratinocytes and dermal fibroblasts) and TIMP-2 accelerates wound healing (i.e., increased migration and proliferation of epidermal cells) [65,66]. Therein, topical application of TIMP-2 in a mouse model of chronic dermatitis showed improved symptoms (i.e., less inflammation, reduced epidermal thickness) [67]. A significant role of MMPs is the degradation of fibrillar collagens in extracellular matrix remodeling has been documented [68].

Regarding detected soluble cytokine receptors and other cytokines, soluble gp130 (sgp130) possesses the ability to bind the sIL-6R-Il-6 complex and block trans-signaling pathways, which may be positive for skin barrier function modulation [69,70,71]. sVEGFR1 inhibits VEGFR1 and VEGFR2, wherein regulation of VEGFR expression levels seems important for wound healing and might be compromised in chronic and refractory wounds [72,73,74]. Other detected cytokines such as MCP-1, GRO-a, or fractalkine (CX3CL1) possess chemo-attractive functions toward mononuclear cells, neutrophils, T cells, and monocytes [75,76,77].

Overall, all the main protein components identified and present in relatively important quantities in the cell lysates and lyophilizates submitted to investigation were determined to possess a physiological function or effect which may be interpreted as useful or instrumental in the context of a cutaneous regenerative medicine product development. It is probable that the overall effect of such cell-based preparations or derivatives is a resultant (i.e., additive or synergistic effects) of individual contributions of specified protein components or factors. The combined nature of such effects may be key for product function, as indirectly illustrated by the non-substantial modifications in interleukin (i.e., IL-8) production by adult fibroblasts in the TNF challenge assays (Figure 9). Therein, it is probable that further study of individual mechanisms (e.g., inflammation-related pathways) may be unsatisfactory for the provision of a purely pharmacological mechanism of action of considered biological APIs. Therefore, alternative assays should be chosen or designed to be more representative or adapted to functional characterization of the substances of interest, as few single molecular targets or mechanisms may be outlined to explain the observed clinical effects of FPC-based product applications [20].

### 4.4. Tangible Advantages of Off-the-Shelf FPC-Based Therapeutic Products

The use of appropriate techniques (e.g., lyophilization) for processing and stabilization of standardized cellular materials potentially enables further and consistent access to such substances in the context of regenerative medicine use thereof. Indeed, classical workflows in live cell therapy administration require storage in liquid nitrogen, dry-ice shipping, and short-term storage of products in ultra-low temperature freezers [78,79,80,81,82,83]. This type of cold chain maintenance has been demonstrated as feasible in the context of the unified vaccination efforts against SARS-CoV-2 viruses (e.g., cold chain maintenance for Moderna and Pfizer COVID-19 vaccines), although the logistical costs and delays restrict the availability of sensitive products to relatively small patient populations [84]. Therefore, consistent obtention of stable biologicals which maintain the desired and appropriate activity is of high interest for technically bridging the gap between clinician expectations and the potential of novel cell-based or cell-derived cell-free therapeutic products or medical devices [85,86,87]. This would be made possible in an off-the-shelf setting by the obtention of stable and active cell-based preparations to be extemporaneously reconstituted on an appropriate scaffold, instead of on-demand cell initiation from cryostorage for TrSt preparation (e.g., current version of PBBs) [36]. Therein, the development and proper homologation of FPC preparations (i.e., cells in various states of preservation, either cryopreserved or lyophilized, or further processed into cell-free materials) appears to be a critical pathway to be further investigated for consistency, safety, availability, sustainability, and regulatory compliance assurance.

## 5. Conclusions

The present study describes extensive process and parameter optimization within processing of dermal FPC sources in view of eventual off-the-shelf cell-based therapeutic product development for cutaneous regenerative medicine. Cell banking workflows proposed for FE002-SK2 FPCs potentially enable the consistent manufacture of several billion therapeutic cell-based or cell-derived cell-free products for allogeneic homologous applications. Furthermore, original data was presented on the optimization of FE002-SK2 cell stabilization by lyophilization, demonstrating the maintenance of important composition and function of the processed biological materials, as compared to equivalent fresh cell preparations. Specifically, protein contents and in vitro stimulatory potentials of selected lyophilizates were shown to be of interest in natural processes of cutaneous wound closure and tissular repair. The data presented herein establish the technical basis for studying and applying next-generation off-the-shelf topical regenerative medicine therapeutic products in translational settings. Therefore, further preclinical validation of the functional equivalence of fresh and lyophilized FE002-SK2 FPCs would potentially be a major step to enable global technical and clinical progress in novel medical frameworks dedicated to generalized betterment of patient health.

## Figures and Tables

**Table 1:** Results of multi-parameter optimization and corresponding grading of raw and ancillary materials, contact-process consumables, and culture conditions for large-scale manufacturing of dermal FPCs. FBS is considered here both as a raw and ancillary material, as it is used as a culture medium supplement and as a component of the cell cryopreservation medium. Optimal culture surfaces were selected based on observed proliferative cellular behaviour and harvest cell yields. Optimal FBS sources were chosen based on harvest cell yields and availability of large commercial lots of the appropriate quality. Optimal cell seeding densities, culture medium volumes, and culture periods were selected based on cellular behaviour in culture, harvest cell yields, and sparing use of cell seed stocks, materials, and manufacturing suite use, respectively. Grading was performed using the abbreviated nomenclature presented hereafter. (–) = unsatisfactory, (+) = sub-optimal, (++) = satisfactory, (+++) = optimal. FBS, fetal bovine serum; FPC, fibroblast progenitor cells.

Optimized Parameter	Considered Indicators and Endpoints	Parameter-Specific Targets	Tested Options	Grading ^1^
Culture vessel surface (cm^2^)	Cellular proliferative morphology and behaviour Harvest cell yields	Specific morphology maintenance Homogenous cell monolayer growth Maximized production cell yield	175 cm^2^—T175 225 cm^2^—T225 500 cm^2^—T500	+++ +++ +
FBS source & lot identity	Cellular proliferative morphology and behaviour Harvest cell yields	Specific morphology maintenance Homogenous cell monolayer growth Maximized production cell yield	Sigma^®^—lot A Sigma^®^—lot B Sigma^®^—lot C Invitrogen^®^—lot A Invitrogen^®^—lot B HyClone™—lot A	+++ + +++ ++ – +
Seeding densities (viable cells/cm^2^)	Total culture period Harvest cell yields	Minimized culture period Minimized cell seed stock usage Maximized production cell yield	1.5 × 10^3^ cells/cm^2^ 3.0 × 10^3^ cells/cm^2^ 5.0 × 10^3^ cells/cm^2^ 10^4^ cells/cm^2^	+++ +++ ++ +
Culture medium volume (mL/cm^2^)	Total culture period Harvest cell yields	Minimized culture period and media consumption Maximized production cell yield	0.05 mL/cm^2^ 0.10 mL/cm^2^ 0.15 mL/cm^2^ 0.20 mL/cm^2^ 0.25 mL/cm^2^ 0.30 mL/cm^2^	– + +++ +++ ++ +
Culture periods (days)	Total culture period Harvest cell yields	Minimized culture period Maximized production cell yield	8 days 10 days 12 days 15 days	– + +++ +++

^1^ Overall experimental grades were attributed to each considered option for the selected parameters to optimize, based on the continued assessments of two specialized manipulators qualified for in vitro culture of dermal FPCs. Grading of the various culture surfaces was largely centered on the endpoint harvest cell yields, which are shown to be progressively negatively impacted by the use of relatively larger culture surfaces (Figure 1). Grading of the various FBS sources was based largely on endpoints harvest cell yields, however some serums induced atypical cellular morphologies or reactions and were therefore graded poorly on the applied semi-quantitative scale. Seeding densities were graded almost exclusively on the endpoint harvest cell yields, in order to ensure sparing use of constituted cell stocks. Culture medium volumes were graded almost exclusively on the endpoint harvest cell yields, in order to ensure sparing use of GMP-grade consumables. Culture periods were graded almost exclusively on the endpoint harvest cell yields, in order to ensure best overall cost-effectiveness of the rented GMP manufacturing platform.

**Table 2:** Results of descriptive analysis, selected characterization testing, and corresponding grading for the obtained lyophilizates 48 h after lyophilization of dermal FPCs, formulated in adapted cryo- and lyo-protective solutions. Parameters considered as lyophilized product CQAs are evidenced with an * in the table hereunder. Formula I was experimentally assessed as optimal in the considered test conditions. Grading was performed using the abbreviated nomenclature presented hereafter. (–) = unsatisfactory, (+) = sub-optimal, (++) = satisfactory, (+++) = optimal. CQA, critical quality attribute; FPC, fibroblast progenitor cells; RH, relative humidity.

Parameter	Target	Acceptance Criteria (Cumulative)	Grading of Optimal Formulas		
			N°B	N°F	N°I
Presence of cake *	Presence of a solid cake	Presence of a solid cake No residual liquid phase	+	+	+++
Batch uniformity *	Uniform batch	Vial-to-vial uniform aspect Dry product unitary mass uniformity ^1^	++	++	+++
Cake colour	White cake colour	White cake coloration Monochrome cake Consistent hue, tint, tone, and shade	++	++	+++
Cake structure	Uniform structure	Presence of a single cylindrical solid mass	+	+	+++
Cake density	Dense cake	Presence of small cake pores Absence of gross porosity on sides and bottom of cake	++	++	+++
Cake finish	Shiny finish ^2^	Shiny or sheen finish observed on the top, sides, and bottom of the cake Absence of matte finish	++	++	++
Cake friability	Non-friable cake ^3^	No detachment or detachment of small fragments from the quoins of the cake Free fragments <5% of total cake volume	+	–	+++
Cake topography	Consistent cake topography	Consistent presence of top flakes, bumps, cracks, concavity, or peaks	–	–	++
Cake shrinkage	Minimal cake shrinkage	No horizontal shrinkage Vertical shrinkage <10% from original fill height	–	+	+++
Cake collapse/ meltback	No cake collapse or meltback	Absence of collapse Absence of observable liquid portion of the cake	+	++	+++
Residual material presence	Minimal residual material presence	Minimal residual material present on the upper rim of the cake, on vial surface at the original fill height	+++	+++	+++
Particle presence	Absence of observable contaminating particles	Absence of observable contaminating particles ^4^	+++	+++	+++
Residual moisture level *	Residual moisture level <2% ^5^	Residual moisture level <2%	+++	+++	+++
Cake reconstitution *	Full reconstitution time <90 s ^6^	Absence of observable solid and undissolved mass after 90 s	+++	+++	+++
Cell structural integrity maintenance	Presence of structurally integral cells	Structural integrity confirmed microscopically and by size distribution analysis ^7^	++	+++	+++
Cellular viability upon reconstitution	Absence of viable cells	Absence of viability confirmed by staining of cells with Trypan blue	+++	+++	+++

^1^ Product mass uniformity determination is based on method 2.9.5. “*Uniformity of mass of single-dose preparations*” of the current European Pharmacopoeia (EP) and the acceptance level is set at mean mass ± 10%. ^2^ Products are examined under direct laboratory lighting. ^3^ Cake friability is assessed by vortexing vials at maximum speed for 10 s on a laboratory vortex. ^4^ Assessment of particle presence is based on EP method 2.9.20. “*Particulate contamination: Visible particles*”. ^5^ Residual moisture level is determined by the Karl-Fisher method. ^6^ Reconstitution is assessed after addition of the adequate solvent (i.e., same volume as initial vial fill volume) and gentle manual shaking of the vials. ^7^ Size distribution analysis is carried out using Mastersizer instruments. From a rationale standpoint, it is to note that the first 12 reported parameters were comparatively graded for the 3 formulas of interest in view of ensuring intra-batch and inter-batch homogeneity and repeatability. Therein, no specific parameter was individually optimized, and the overall goal of the lyophilization process optimization consisted in obtaining a coherent structured lyophilizate with sufficient resistance and stability in the defined experimental conditions. The residual moisture level was graded with the objective of obtaining a stable lyophilizate (i.e., sufficiently dehydrated), but to avoid excessive water removal, which is known to adversely impact both structure and function of proteins. Grading of cake reconstitution was guided by the rapidity and simplicity of resuspending the lyophilized materials, which is a key element for extemporaneous treatment reconstitution. In view of specific regulatory classification workflows, cellular structural integrity maintenance and devitalization were assessed, in view of confirming the state of the active substance after processing (i.e., confirm the presence of whole cell units) and to exclude the presence of viable materials after processing.

**Table 3:** Quantitative results of primary keratinocyte proliferation stimulation by dermal FPC lysates and lyophilizates in homeostasis medium or in proliferation medium, respectively, for each group. In all individual settings, a significant and dose-dependent promotion of keratinocyte proliferation was obtained by the addition of the treatment, as compared to the control wells. When considering each medium separately, no statistically significant difference was found between the ability of cell lysates and the ability of cell lyophilizates to promote keratinocyte proliferation. Cell lysates were obtained by freeze-thaw (FT) cycles and lyophilizates were obtained using formula B for stabilization processing. Values are presented as average cell counts after harvest, with corresponding standard deviations. FPC, fibroblast progenitor cells; FT, freeze-thaw; LYO, lyophilizate.

Product Dosage Final Concentration in the Culture Medium (µg/mL) ^1^	Keratinocyte Count in Product-Supplemented Homeostasis Medium (10^3^ Cells)		Keratinocyte Count in Product-Supplemented Proliferation Medium (10^3^ Cells)	
	FE002-SK2-FT	FE002-SK2-LYO	FE002-SK2-FT	FE002-SK2-LYO
0	194 ± 10	194 ± 10	68 ± 3	68 ± 3
5	218 ± 20	202 ± 6	116 ± 8	98 ± 12
15	239 ± 29	247 ± 32	131 ± 9	128 ± 14

^1^ Product concentration is expressed in total protein content, as determined by BCA assay.

## Data Availability

The data presented in this study are available on request from the corresponding author. The data are not publicly available due to legal and statutory restrictions.

## Supplementary Materials

The following are available online at https://www.mdpi.com/article/10.3390/biomedicines9081072/s1. The document “Supplementary Materials” contains the Supplementary Methods, the Supplementary Figures as follows; Figure S1: Swiss progenitor cell biobanking program, Figure S2: Simultaneous differential dermal FPC isolation workflow, Figure S3: Detailed dermal FPC mechanical isolation process, Figure S4: Detailed dermal FPC enzymatic isolation process, Figure S5: FE002-SK2 dermal FPC morphological traits, Figure S6: FE002-SK2 dermal FPC diploid karyotype, Figure S7: FE002-SK2 dermal FPC DNA fingerprinting, Figure S8: FE002-SK2 dermal FPC representative TEM micrographs, Figure S9: FE002-SK2 dermal FPC p63 marker analysis, Figure S10: FE002-SK2 dermal FPC surface marker profiling, Figure S11: Optimized workflow for primary FPC type establishment, Figure S12: Benchmark optimization of FBS sourcing for FPC manufacture, Figure S13: Validation of dissociation reagent equivalence, Figure S14: Passage nomenclature definition for FE002-SK2 FPCs, Figure S15: Optimized multi-tiered banking of dermal FPCs, Figure S16: Optimized manufacture of stabilized dermal FPCs, Figure S17: Stabilization of dermal FPCs by lyophilization, and the Supplementary Table S1: Proteomic data for the qualitative and quantitative composition determination of lyophilized dermal FPC preparations. The document “Process Parameters” contains definitions of the optimized process parameter targets, controls, and acceptance criteria relative to FPC sourcing, manufacturing, and lyophilization, respectively. The document “Risk Analysis Matrix” contains general and specific risk analyses relative to FPC sourcing and manufacturing, respectively.

## Abbreviations

API	active pharmaceutical ingredient
ATMP	advanced therapy medicinal product
BCA	bicinchoninic acid assay
BPyV	bovine polyomavirus
BSA	bovine serum albumin
CD	cluster of differentiation
CHUV	Centre hospitalier universitaire Vaudois
CPP	critical process parameter
CQA	critical quality attribute
DMEM	Dulbecco’s modified Eagle medium
DMSO	dimethyl sulfoxide
DNA	deoxyribonucleic acid
EBV	Epstein-Barr virus
EC	European Commission
ECACC	European collection of authenticated cell cultures
EDQM	European Directorate for the Quality of Medicines & Healthcare
EDTA	ethylenediaminetetraacetic acid
ELISA	enzyme-linked immunosorbent assay
EOPCB	end of production cell bank
EP	European Pharmacopoeia
ESC	embryonic stem cells
FACS	fluorescence activated cell-sorting
FBS	fetal bovine serum
FDA	US Food and Drug Administration
FPC	fibroblast progenitor cells
FT	freeze-thaw
GMP	good manufacturing practices
HAV	hepatitis A virus
HBV	hepatitis B virus
HBoV	human bocavirus
hCMV	human cytomegalovirus
HCV	hepatitis C virus
HHV	human herpes virus
HIV	human immunodeficiency virus
HPV	human papilloma virus
HTLV	human T-cell lymphotropic virus
IPC	in-process control
KIPyV	KI polyomavirus
KPP	key process parameter
LYO	lyophilizate
MCB	master cell bank
MoA	mechanism of action
MSC	mesenchymal stem cells
PBB	Progenitor Biological Bandage
PBS	phosphate-buffered saline
PCB	parental cell bank
PDT	population doubling time
PDV	population doubling value
PPC	post-process control
RH	relative humidity
SD	standard deviation
SV40	simian virus 40
TEM	transmission electron microscopy
TEP	tissue engineering product
TrSt	standardized transplant product
USA	United States of America
WCB	working cell bank
WUPyV	WU polyomavirus

## Appendix A

Contents presented in Appendix A refer to the optimization steps performed in view of obtaining appropriate dermal FPC lyophilizates.

**Table A1:** Qualitative and quantitative compositions of the cryo- and lyo-protective solutions used for lyophilization of dermal FPCs. Formulation tonicity was corrected upon lyophilizate reconstitution, by addition of water and PBS in appropriate proportions. Quantities are presented in *m/v* percentages for sugars, salts, and polymers, and in *v*/*v* percentages for buffers and solvents. DMSO, dimethyl sulfoxide; FPC, fibroblast progenitor cells; PBS, phosphate-buffered saline; PVP, polyvinylpyrrolidone.

Formula ID	Individual Components of Cryo- and Lyo-Protective Solutions Used for Lyophilization of FPCs								
	Saccharose	Mannitol	Lactose	Dextran	PVP	DMSO	PBS	NaCl	Water
A	5	2	3	0	0	0.2	0	0	Ad. 100
B	5	1	4	0	0	0.2	0	0	Ad. 100
C	5	0	3	2	0	0.2	0	0	Ad. 100
D	5	0	3	0	2	0.2	0	0	Ad. 100
E	6.5	0	0	3.5	0	0.2	0	0	Ad. 100
F	6.5	3.5	0	0	0	0.2	0	0	Ad. 100
G	6.5	0	0	0	3.5	0.2	0	0	Ad. 100
H	6.5	0	3.5	0	0	0.2	0	0	Ad. 100
I	8	0	0	2	0	0.2	0	0	Ad. 100
J	8	0	0	0	2	0.2	0	0	Ad. 100
K	0	0	0	0	0	2	0	0	Ad. 100
L	0	0	0	0	0	10	0	0	Ad. 100
M	0	0	0	0	0	0	100	0	0
N	0	0	0	0	0	0	0	100	0

**Table A2:** Experimental results of thermal and electrical property determination of selected cryo- and lyo-protectant formulas used to lyophilize dermal FPCs. Presented data were experimentally gathered during freezing/cooling and heating phases of placebo formulations using formulas B, F, and I. FPC, fibroblast progenitor cells; Zsinφ, impedance analysis parameter.

Process Phase ID	Investigated Parameter	Lyophilization Formula ID		
		N°B	N°F	N°I
Freezing/cooling phase	Subcooling temperature (°C)	−11 ± 2	−13 ± 2	−12 ± 2
	Crystallization temperature (°C)	−2 ± 2	−2 ± 2	−2 ± 2
	Temperature when Zsinφ = 0 MΩ (°C)	−7 ± 2	−10 ± 2	−10 ± 2
	Temperature when Zsinφ = 1 MΩ (°C)	−15 ± 2	−16 ± 2	−18 ± 2
Heating phase	Temperature at beginning of fusion (°C)	−10 ± 2	−11 ± 2	−11 ± 2
	Temperature when Zsinφ = 0 MΩ (°C)	−4 ± 2	−3 ± 2	−3 ± 2
	Temperature when Zsinφ = 1 MΩ (°C)	−13 ± 2	−14 ± 2	−11 ± 2

**Table A3:** Optimized lyophilization cycle parameters (i.e., fluid temperature, chamber pressure, step duration) for stabilization of dermal FPCs using the optimized ad hoc cryo- and lyo-protecant formulas. FPC, fibroblast progenitor cells.

Process Phase ID	Optimized Parameters (Temperature, Pressure, and Time)
A. Loading	Ambient temperature; 1 atm
B. Freezing	−45 °C; 1 atm; 2 h
C. Chamber vacuum	0.08 mbar
D. Primary drying	Ramp from −10 °C to −30 °C and then ramp from −30 °C to 25 °C; 0.08 mbar; 39 h
E. Secondary drying	Ramp from 25 °C to 20 °C; maximal vacuum; 9 h
