# Supplementary material for: Optimized Manufacture of Lyophilized Dermal Fibroblasts for Next-Generation Off-the-Shelf Progenitor Biological Bandages in Topical Post-Burn Regenerative Medicine
Source: Biomedicines. 2021 Aug 23;9(8):1072. doi: 10.3390/biomedicines9081072 (PMC8394413; doi:10.3390/biomedicines9081072)
Supplement: Supplementary file 1 [file biomedicines-09-01072-s001.zip › Supplementary Documents/Process Parameters.pdf]

Process Parameters

Contents presented in this Supplementary Document are relative to the optimization steps performed in view of obtaining standardized workflows for dermal FPC procurement and isolation, culture expansion in vitro, and stabilization by lyophilization, respectively.

Table PP1. Definition of KPPs and CPPs within the optimized primary FPC type establishment workflow in relation with Figure S11. For each item, the predefined targets and the IPCs or PPCs to be appropriately implemented are listed, along with the corresponding acceptance criteria. API, active pharmaceutical ingredient; CPP, critical process parameter; EOPCB, end of production cell bank; FPC, fibroblast progenitor cells; IPC, in-process control; KPP, key process parameter; PCB, parental cell bank; PPC, post-process control; RH, relative humidity; TEM, transmission electron microscopy; WCB, working cell bank.

**CPP KPP**	**Targets**	**IPCs/PPCs**	**Acceptance criteria** (cumulative)
**CPP1**	●Preliminary inclusion of qualifying and qualified donor in the FPC transplantation program<br>●Exclusion of non-qualifying donors	●Medical screening<br>●Health questionnaire administration<br>●First serological testing before donation at T_0	Pregnancy resulting from natural insemination; donor undergoing voluntary and therapeutic pregnancy interruption; donor age between 18 and 25 years; gestational age of 14 weeks (i.e., 12 weeks post-amenorrhea); good overall health [1]; donor serological tests negative for specified pathogens [2]; donor not on any anti-inflammatory treatment during the last six months; donor capable of giving clear informed consent; donor can be reached three months after the donation for repeated blood sampling, testing, and consent confirmation.
**CPP2**	●Obtention of donor consent for pregnancy termination<br>●Obtention of donor consent for inclusion in the FPC transplantation program	●Specific consent forms for pregnancy termination<br>●Specific consent forms for inclusion in the FPC transplantation program	Documented evidence of consent for both pregnancy termination and inclusion in the FPC transplantation program.<br>*Although consent obtention is a target, no influence should be exerted on the donor for either consent categories (i.e., first, the pregnancy termination, followed by inclusion in the transplantation program).*
**CPP3**	●No physical abnormalities of the donation revealed upon pathological investigation	●Anatomopathology<br>●Histopathology	No observed general anomalies. No observed specific anomalies in the umbilical cord, cardiovascular, respiratory, urogenital, digestive, endocrine, hematopoietic, musculoskeletal, or central nervous systems.
**CPP4**	●Normal karyotype of cells from the tissue of interest	●Karyotyping analysis of primary cell cultures	No evidence of the significant presence of chromosomal abnormalities (i.e., hyperploidy, hypoploidy, polyploidy, aberrations, breaks, gaps, dicentrics, fragments, triradials, or rings) upon analysis.
**CPP5**	●Adherence of ≥70% of cells after 24 h of incubation	●Visual monitoring after 24 h of incubation	Cell adherence ≥70% after 24 h of incubation.<br>*This parameter is valid for enzymatic tissue treatment. For mechanical tissue treatment, replace "cells" by "tissue fragments".*
**CPP6**	●Confirmation of positive confluency evolution between medium exchanges	●Visual monitoring at medium exchanges<br>●Photographic recording of cultures	(Confluency value at medium exchange X) ≥ (Confluency value at medium exchange X-1).
**CPP7**	●No contaminating pathogens in retention samples	●Testing for bacteria, fungi, viruses, endotoxins, mycoplasma	Absence of detection for specified contaminants or values of detection < to specified thresholds. Absence of detection for non-specified contaminants.

**CPP8**	●Specific cellular morphology and behaviour maintenance in culture<br>●Absence of multiple cell populations in culture	●Visual monitoring of cultures during expansion<br>●Photographic recording of cultures	Spindle-shaped cells; distinctive fibroblastic phenotype; absence of multiple cell populations.
**CPP9**	●Final donor qualification<br>●Donor inclusion in the FPC transplantation program	●Repeated serological testing at $T_{0+90\ days}$<br>●Specific consent forms for inclusion in the FPC transplantation program	No seroconversion for specified pathogens [2] at the three-month timepoint after the donation.<br>Documented evidence of consent for inclusion in the FPC transplantation program.
**CPP10**	●Cell type lifespan >9 in vitro passages<br>●No modifications in the karyotype after expansion	●In vitro lifespan determination<br>●Iterative karyotyping analyses	Cell type lifespan >9 in vitro passages; no modifications in the karyotype from preliminary cultures up to EOPCB materials.
**CPP11**	●No contamination of biological material stocks	●Iterative contaminant screening	Absence of detection of bacteria, non-specified and specified viral contaminants [3], fungi, mycoplasma, endotoxins, reverse transcriptase activity, or observable (TEM) contaminants [4].
**CPP12**	●Absence of cell type toxicity<br>●Absence of tumor formation risk	●Qualification of pilot EOPCB for sterility, toxicity, tumorigenicity	Successful qualification of pilot EOPCB. Absence of tumorigenicity evidence in vitro, in ovo, and in vivo.
**CPP13**	●Specific cellular morphology and behaviour maintenance in culture<br>●Absence of multiple cell populations in culture	●Visual monitoring during expansion<br>●Marker panel analysis	Spindle-shaped cells; distinctive fibroblastic phenotype; absence of multiple cell populations; consistent cell surface marker profiles; absence of p63 marker detection for dermal FPCs.
**CPP14**	●Stable storage tank temperature	●Storage tanks temperature logs	Storage tank temperature constantly ≤ -145 °C.
**CPP15**	●Availability of all relevant documents and records for cell type master file, biobank inventory, and manufacturing batch files	●Follow-up of manufacturing data in cell type master file and storage data in biobank master inventory	Availability of all necessary authenticated records and documents at the time of reconciliation.
**KPP1**	●Maintenance of adequate culture conditions throughout expansion	●Incubator data logs	Relative limits [5] of 37 °C ± 2 °C, 5.0% ± 0.5% CO_2, 85% ± 5% RH.<br>Absolute limits [6] of 34 °C ± 5 °C, 0% – 6% CO_2, 50% – 95% RH.
**KPP2**	●Homogenous growth of cell monolayer over available culture surfaces	●Visual monitoring during expansion<br>●Photographic recording of cultures	Absence of unpopulated culture surface of ≥15% of total available culture surface in each vessel.

**KPP3**	●Consistent preliminary culture times with historic data	●Visual monitoring during expansion<br>●Photographic recording of cultures	Harvest of preliminary cultures at 15 ± 3 days.

[1] As assessed by specific anonymized health questionnaire. [2] Specified pathogens for donor serology screening comprise CMV, HIV-1, HIV-2, HBV, HCV, HTLV-1, HTLV-2, S-West Nile virus, Zika virus, Treponema pallidum, Toxoplasma gondii. [3] Specified detectable viral contaminants comprise adenovirus, B19 parvovirus, BPyV, EBV, HuPyV, HPV, HBoV, HAV, HBV, HCV, hCMV, HIV-1, HIV-2, HTLV-1, HTLV-2, HHV-6, HHV-7, HHV-8, KIPyV, orthomyxoviruses, paramyxoviruses, picornaviruses, reoviruses, S-West Nile virus, Zika virus, WUPyV, and SV40. [4] Specified observable contaminants comprise viruses, virus-like particles, mycoplasma, yeasts, fungi, or bacteria, assessed on >200 cell sections by TEM. [5] Relative limits refer to the individual and successive incubation phases between initiation, medium exchanges, and harvest (i.e., closed incubator doors). [6] Absolute limits refer to overall culture maintenance, from initial incubation up to harvest (i.e., considering incubator door opening for culture handling).

Table PP2. Definition of KPPs and CPPs within the optimized dermal FPC banking workflow in relation with Figure 2. For each item, the predefined targets and the IPCs or PPCs to be appropriately implemented are listed, along with the corresponding acceptance criteria. CPP, critical process parameter; FPC, fibroblast progenitor cells; IPC, in-process control; KPP, key process parameter; PCB, parental cell bank; RH, relative humidity; WCB, working cell bank.

**CPP KPP**	**Targets**	**IPCs/PPCs**	**Acceptance criteria** (cumulative)
**CPP1**	●Initiation of the correct vial lot	●Batch record and vial label reconciliation	Correspondence between initiation form, vial batch record, and vial labels.
**CPP2**	●No contaminating pathogens in biological starting material and in culture environment	●Asepsy testing upon culture initiation<br>●Testing for specified and non-specified contaminants	Absence of detection for specified and non-specified contaminants or values of detection < to specified thresholds.
**CPP3**	●Cellular viability ≥85% upon recovery	●Viable cell enumeration	Cellular viability ≥85% upon initiation.
**CPP4**	●Adherence of ≥80% of cells after 24 h of incubation	●Visual monitoring of cultures after 24 h of incubation	Cell adherence ≥80% after 24 h of incubation in every culture vessel.
**CPP5**	●Confirmation of positive confluency evolution between medium exchanges	●Visual monitoring at medium exchanges<br>●Photographic recording of cultures	(Confluency value at medium exchange X) ≥ (Confluency value at medium exchange X-1).
**CPP6**	●No contaminating pathogens in processed biological materials and in culture environment	●Asepsy testing upon culture initiation<br>●Testing for specified and non-specified contaminants	Absence of detection for specified and non-specified contaminants or values of detection < to specified thresholds.
**CPP7**	●Specific cellular morphology and behaviour maintenance in culture<br>●Absence of distinct cell populations in culture	●Visual monitoring of cultures during expansion<br>●Photographic recording of cultures	Spindle-shaped cells; distinctive fibroblastic phenotype; absence of multiple cell populations; consistent cell surface marker profiles; absence of p63 marker detection for dermal FPCs.
**CPP8**	●No contaminating pathogens in conditioned biological materials and in culture environment	●Asepsy testing upon culture initiation<br>●Testing for specified and non-specified contaminants	Absence of detection for specified and non-specified contaminants or values of detection < to specified thresholds.
**KPP1**	●Correspondence between values specified on initiation form, vial batch record, and vial labels	●Total cell enumeration and cell count reconciliation	Tolerance of ± 30% of total cell count from labelled vial contents.

**KPP2**	●Maintenance of adequate culture conditions throughout expansion	●Incubator data logs	Relative limits [1] of 37 °C ± 2 °C, 5.0% ± 0.5% CO_2, 85% ± 5% RH.<br>Absolute limits [2] of 34 °C ± 5 °C, 0% – 6% CO_2, 50% – 95% RH.
**KPP3**	●Homogenous growth of cell monolayer over available culture surfaces	●Visual monitoring during expansion<br>●Photographic recording of cultures	Absence of unpopulated culture surface of ≥15% of total available culture surface in each vessel.
**KPP4**	●Maintenance of bright to dark red colouring of culture medium containing a phenol red pH indicator	●Visual monitoring of culture medium colour during expansion	Absence of orange colouring of culture medium or signs of severe cell starvation.
**KPP5**	●Consistent cell population doubling times during expansion from PCB to WCBs	●Monitoring of batch manufacturing data during serial expansions	Cell batch harvest at 15 ± 1 days, or a maximum of 18 days.
**KPP6**	●Maximization of production yield<br>●Harvest confluency of 100%	●Endpoint visual assessment of cultures<br>●Endpoint photographic recording of cultures	Confluency ≥95% upon harvest.

[1] Relative limits refer to successive incubation phases between initiation, medium exchanges, and harvest (i.e., closed incubator doors). [2] Absolute limits refer to overall culture maintenance, from initial incubation up to harvest (i.e., considering incubator door opening for culture handling).

Table PP3. Definition of KPPs and CPPs within the optimized dermal FPC lyophilization workflow in relation with Figure 3. For each item, the predefined targets and the IPCs or PPCs to be appropriately implemented are listed, along with the corresponding acceptance criteria. CPP, critical process parameter; FPC, fibroblast progenitor cells; IPC, in-process control; KPP, key process parameter; PPC, post-process control; QC, quality control; SD, standard deviation.

CPP KPP	Targets	IPCs/PPCs	**Acceptance criteria** (cumulative)
**CPP1**	•Appropriate preparation tonicity for specified administration route	•Tonicity measurement in bulk product	Tonicity of 300 ± 35 mOsm/kg before lyophilization processing and after appropriate reconstitution. *Tonicity requirements and acceptable margins may vary depending on the intended product administration route.*
**CPP2**	•Appropriate preparation pH for specified administration route	•pH measurement in bulk product	Measured pH of 7.5 ± 1.0 before lyophilization processing and after appropriate reconstitution.
**CPP3**	•No contaminating pathogens in conditioned biological materials and in culture environment	•Asepsy testing upon processing initiation •Testing for specified and non-specified contaminants	Absence of detection for specified and non-specified contaminants or values of detection < to specified thresholds.
**CPP4**	•Vial stoppers in open position at the time of loading in lyophilizator	•Visual inspection of loaded vial batch	All vial stoppers observed to be open.
**CPP5**	•Identical contact surface between each vial and the lyophilizator shelf	•Visual inspection of loaded vial batch	All vials observed to be identically in contact with the lyophilizator shelf.
**CPP6**	•Presence of samples in frozen state at the time of loading in the lyophilizator	•Visual inspection of vial batch	All vials observed to be frozen.
**CPP7**	•Shelve temperature in adequation with specified value	•Lyophilizator monitoring system and logs	Shelve temperature at the specified value ± 1.5 °C.
**CPP8**	•Chamber vacuum in adequation with specified value	•Lyophilizator monitoring system and logs	Chamber vacuum at the specified value ± 0.03 mbar during primary drying. Chamber vacuum at the specified value ± 0.03 mbar during secondary drying.
**CPP9**	•Complete endpoint stoppering of all vials	•Visual inspection of vial batch	Observed complete stoppering of all vials.
**CPP10**	•Consistency in intra-batch and inter-batch cake aspect	•Descriptive QC	Overall and detailed appreciation of cake aspect within historical data brackets.

**CPP11**	●Removal of consistent water quantities between manufacturing batches	●Quantitative QC	Residual relative humidity level within specified mean value brackets; water weight loss within specified mean value brackets.
**CPP12**	●Ability of product to stimulate cell proliferation and migration	●Functional QC	Adequation of product functionality with historical data.
**CPP13**	●No contaminating pathogens in conditioned biological materials	●Testing for specified and non-specified contaminants	Absence of detection for specified and non-specified contaminants or values of detection < to specified thresholds.
**CPP14**	●Availability of all relevant documents and records for manufacturing batch files	●Follow-up of manufacturing and storage data	Availability of all necessary authenticated records and documents at the time of reconciliation.
**KPP1**	●Total lyophilization cycle duration within defined limits	●Lyophilizator logs	Total lyophilization cycle duration within ± 2 SD of mean value, based on historical data.
**KPP2**	●Appropriate level of water removal	●Visual inspection of ice trap icing level<br>●Weighing of removed water from ice trap collection recipient	Appreciation of trapped water quantity within historical data brackets; removed water mass within ± 2 SD of historical mean value.
**KPP3**	●Storage of products at constant temperature	●Refrigerator temperature monitoring system and logs	Stable refrigerator temperature at 5.0 °C ± 3.0 °C.
