# Supplementary material for: Optimized Manufacture of Lyophilized Dermal Fibroblasts for Next-Generation Off-the-Shelf Progenitor Biological Bandages in Topical Post-Burn Regenerative Medicine
Source: Biomedicines. 2021 Aug 23;9(8):1072. doi: 10.3390/biomedicines9081072 (PMC8394413; doi:10.3390/biomedicines9081072)
Supplement: Supplementary file 1 [file biomedicines-09-01072-s001.zip › Supplementary Documents/Risk Analysis Matrix.pdf]

Risk Analysis Matrix

Contents presented in this Supplementary Document are relative to risk analysis and control within the optimization steps performed in view of obtaining standardized workflows for dermal FPC processing.

Table RA1. General risk analysis matrix established for assessment of the sourcing, procurement, and culture initiation of primary FPC types. API, active pharmaceutical ingredient; DNA, deoxyribonucleic acid; EOPCB, end of production cell bank; FACS, fluorescence-activated cell sorting; FPC, fibroblast progenitor cells; PCB, parental cell bank; QC, quality control.

**Parameter**	**Pre-mitigation**			**Risk severity** (0-3) [1]	**Risk likelihood** (0-2) [2]	**Risk level** (0-2) [3]	**Mitigations**	**Post-mitigation risk level** (0-2) [4]
	**Risk**	**Cause**	**Effects**					
Donor qualification	●Seropositivity for specified pathogens<br>●Seropositivity for unspecified pathogens<br>●Seroconversion for specified pathogens	●Inadequate anamnesis<br>●Inadequate testing scheme<br>●Presence of undetectable or latent infection	●Donor qualification failure<br>●Production of contaminated PCB<br>●PCB qualification failure	3	1	2	●Thorough donor anamnesis<br>●Thorough initial serological screening<br>● Thorough repeated serological screening<br>●Use of highly specific and sensitive screening methods	0
Donation qualification	●Anatomical or physiological abnormality<br>●Cytogenetic abnormality	●Inadequate anamnesis<br>●Genetic singularity	●Donation qualification failure	3	1	2	●Extensive donor screening and anamnesis<br>●Thorough pathological investigation<br>●Thorough cytogenetic investigation	0

Cell type instability	●Non-qualification for in vitro culture<br>●Apparition of tumorigenicity/toxicity	●Non-adaptation to in vitro culture<br>●Insufficient quality of genetic materials	●Critical sustainability problematic<br>●Critical safety problematic	3	1	2	●Pilot qualification for in vitro serial expansion<br>●Extensive cytogenetic investigation<br>●Evolutive karyotyping scheme<br>●Establishment and qualification of pilot EOPCB before large scale manufacture	0
Short lifespan	●Inadequacy of cell type for extensive multi-tiered banking<br>●Low resistance to cryopreservation	●Inadequacy of chosen cell type or tissue sample<br>●High sensitivity to cryogenic shock	●Critical quality problematic<br>●Critical sustainability problematic	3	1	2	●Extensive cell sourcing optimization<br>●Monitoring of culture quality parameters<br>●Evolutive karyotyping scheme<br>●Establishment and qualification of pilot EOPCB before large scale manufacture	0
Contamination of cell banks	●Introduction of extraneous contaminants by reagents, equipment, material, personnel<br>●Emergence of latent or transient virus<br>●Cross-contamination by a similar cell strain<br>●Population switch	●Adventitious agent introduction during manufacture, transport, or storage<br>●Inadequate segregation of cultures<br>●Poor initial cell population purity<br>●Inadequate manufacturing process<br>●Insufficient characterization of cell type	●Inadequate cell type in manufactured batch<br>●Contamination of manufactured batch<br>●Critical quality problematic	3	1	2	●Iterative qualification of source cell banks<br>●Aseptic procurement environment<br>●Class A manufacturing environment<br>●Selection of qualified and tested materials and reagents<br>●Environmental controls during open-container manipulations<br>●Minimization of open-container processes<br>●Minimization of contact processes<br>●Use of sterile single-use consumables<br>●Retention sample testing<br>●Post-production cell banks testing and qualification<br>●Identity and purity QCs (i.e., morphology recording, immunostaining, FACS, DNA fingerprinting)	0

Tumorigenicity	●Tumoral proliferation of biological API	●Spontaneous mutation<br>●Cells beyond acceptable in vitro age	●Tumor formation<br>●Critical safety problematic	3	0	1	●Qualification of manufacturing EOPCB for in vitro lifespan confirmation<br>●Full stability testing of manufacturing EOPCB<br>●Full safety testing (i.e., in vitro, in ovo, in vivo) of manufacturing EOPCB<br>●Use of cells ≤ ⅔ of the qualified lifespan, determined from EOPCB passage level	0

[1] Risk severity is classified as (0) acceptable, (1) tolerable, (2) undesirable, or (3) intolerable. [2] Risk likelihood is classified as (0) improbable, (1) possible, or (2) probable. [3] Risk level is classified as (0) low, (1) medium, or (2) high. [4] Post-mitigation risk level is classified as (0) low, (1) medium, or (2) high.

Table RA2. General risk analysis matrix established for assessment of the banking of primary FPC types. API, active pharmaceutical ingredient; DNA, deoxyribonucleic acid; FACS, fluorescence-activated cell sorting; FPC, fibroblast progenitor cells; MCB, master cell bank; PCB, parental cell bank; QC, quality control; WCB, working cell bank.

Parameter	Pre-mitigation			Risk severity (0-3) [1]	Risk likelihood (0-2) [2]	Risk level (0-2) [3]	Mitigations	Post-mitigation risk level (0-2) [4]
	**Risk**	**Cause**	**Effects**					
Cell viability	●Loss of cell viability	●Inadequate storage or handling	●Reduction of manufacturing yield<br>●Reduced batch quality	2	1	1	●Storage stability validation and monitoring<br>●Iterative total and viable cell enumeration<br>●Monitoring of culture quality [5]<br>●Rinsing of detached cells	0
Storage system failure	●Critical rise in vial temperature/thawing<br>●Catastrophic defect in vial structure or in Dewar storage tank system [6]	●Material and equipment failures<br>●System failures<br>●Absence of redundancies	●Loss of vial batch or loss of whole banks	3	0	1	●Use of qualified primary containers (e.g., polymeric vials) and storage tanks (e.g., off-line tanks)<br>●Segregation of high-value vials in redundant facilities<br>●Segregation of high-value vials in redundant tanks<br>●Nitrogen level/temperature monitoring and alarms<br>●Critical failure alarms<br>●Regular inspection of storage tanks<br>●Inspection of vials at the time of initiation/thawing	0

Cross-contamination or population switch	●Cross-contamination by a similar cell strain<br>●Population switch	●Inadequate segregation of cultures<br>●Poor initial cell population purity	●Inadequate cell type introduced during manufacturing	3	1	2	●Iterative identity and purity QCs (i.e., morphology recording, immunostaining, FACS, DNA fingerprinting)<br>●Segregation of cell strains to specific manufacturing areas and equipment<br>●Use of sterile single-use consumables	0
Functional loss of API	●Ineffective product manufacture	●Inadequate cell manufacture or storage	●Rejection of finished product<br>●Ineffective therapeutic intervention	2	1	1	●Monitoring of culture quality<br>●Use of qualified and consistent WCB tier within the in vitro lifespan<br>●Standard functional QCs	0
Adventitious contamination of MCBs	●Contaminated MCB<br>●Non-qualification and rejection of MCB	●Adventitious agent introduction during manufacture, transport, or storage	●Loss of large quantities of cells<br>●Need for MCB reestablishment from PCB	3	1	2	●Qualification of PCB<br>●Class A manufacturing environment<br>●Environmental controls during open-container manipulations<br>●Selection of qualified and tested materials and reagents<br>●Minimization of open-container processes<br>●Minimization of contact processes<br>●Use of sterile single-use consumables<br>●Retention sample testing<br>●Post-production MCB qualification	0

Adventitious contamination of WCBs	●Contaminated WCB<br>●Non-qualification and rejection of WCB	●Adventitious agent introduction during manufacture, transport, or storage	●Loss of cell batch<br>●Need for WCB reestablishment from MCB	2	1	2	●Qualification of source MCB<br>●Class A manufacturing environment<br>●Environmental controls during open-container manipulations<br>●Selection of qualified and tested materials and reagents<br>●Minimization of open-container processes<br>●Minimization of contact processes<br>●Use of sterile single-use consumables<br>●Retention sample testing<br>●Post-production WCB qualification	0
Tumorigenicity	●Tumoral proliferation of biological API	●Spontaneous mutation<br>●Cells beyond acceptable in vitro age	●Tumor formation	3	0	1	●Qualification of manufacturing EOPCB for in vitro lifespan confirmation<br>●Full stability testing of manufacturing EOPCB<br>●Full safety testing (i.e., in vitro, in ovo, in vivo) of manufacturing EOPCB<br>●Use of cells ≤ ⅔ of the qualified lifespan, determined from EOPCB passage level	0

[1] Risk severity is classified as (0) acceptable, (1) tolerable, (2) undesirable, or (3) intolerable. [2] Risk likelihood is classified as (0) improbable, (1) possible, or (2) probable. [3] Risk level is classified as (0) low, (1) medium, or (2) high. [4] Post-mitigation risk level is classified as (0) low, (1) medium, or (2) high. [5] Monitoring includes proliferative cellular morphology, cell adhesion, growth rate, confluency level, cell monolayer homogeneity, sub-population exclusion, and gross contamination exclusion. [6] Includes rupture or explosion of vials and catastrophic defect in liquid nitrogen auto-filling system.

Table RA3. Specific risk analysis matrix established for assessment of the viral safety of primary FPC types, based on EP chapter 5.1.7. "*Viral safety*" (i.e., interpreted in conjunction with ICH Q5A R1), considering the cells as cryopreserved APIs for medicinal products. The viral safety of the materials serving for manufacture of medicinal products is appropriately ensured by general and specific measures at the time of selection of starting, raw, and ancillary materials and testing thereof, before production, during production, and during post-production testing. A thorough risk assessment is necessary, since the considered aseptic process is devoid of a sterilization step or viral agent removal/inactivation step. API, active pharmaceutical ingredient; EOPCB, end of production cell bank; EP, European pharmacopoeia; FPC, fibroblast progenitor cells; MCB, master cell bank; PCB, parental cell bank; QC, quality control; TEM, transmission electron microscopy; WCB, working cell bank.

**Parameter**	**Pre-mitigation**			**Risk severity** (0-3) [1]	**Risk likelihood** (0-2) [2]	**Risk level** (0-2) [3]	**Mitigations**	**Post-mitigation risk level** (0-2) [4]
	**Risk**	**Cause**	**Effects**					
Species of origin	●Risk of infection by zoonotic viruses	●Inclusion of infected donor materials	●Zoonotic viral contamination of API and infection of patient	3	0	1	●Selection of human starting materials<br>●Thorough testing for pathogens with human tropism	0
Tissue of origin	●Use of contaminated starting materials	●Vertical transmission of pathogens<br>●Use of tissue type prone to contamination	●Contamination of API<br>●Infectious risk for the patient	3	1	1	●Selection of tissue with low probability of high contaminant yield<br>●Thorough qualification of donor<br>●Thorough qualification of donation	0

History of the donor, vertical transmission of pathogens	•Unknown seropositivity of donor<br>•Seroconversion for specified pathogens<br>•Seropositivity of donor for unspecified pathogens	•Inadequate donor anamnesis<br>•Inadequate viral screening of donor<br>•Presence of latent viral infection	•Contamination of API<br>•Infectious risk for the patient	3	1	1	•Thorough donor anamnesis<br>•Thorough initial serological screening<br>• Thorough repeated serological screening<br>•Use of highly specific and sensitive viral screening methods	0
Viral contamination during API manufacturing	•Introduction of extraneous viral contaminant by reagents, equipment, material, personnel<br>•Emergence of latent or transient virus in culture	•Inadequate manufacturing process<br>•Inadequate control process<br>•Insufficient initial characterization of cell type<br>•Presence of latent virus in materials<br>•Absence of purification regimen and terminal sterilization	•Contamination of API<br>•Infectious risk for the patient	3	1	2	•Qualification of source cell banks<br>•Class A manufacturing environment<br>•Selection of qualified and tested materials and reagents<br>•Environmental controls during open-container manipulations<br>•Minimization of open-container processes<br>•Minimization of contact processes<br>•Use of sterile single-use consumables<br>•Retention sample testing<br>•Post-production cell bank testing and qualification<br>•Post-production bulk product and final product testing and qualification	0

Infectivity or iatrogenesis of contaminated API	●Iatrogenic infection of patient<br>●Inadequate management of patient pathology<br>●Systemic viral infection	●Contaminated API<br>●Non-functional or potentially iatrogenic API<br>●Invasive product administration route	●Infectious risk for the patient<br>●No amelioration or worsening of patient health status	3	1	2	●Qualification of WCBs<br>●Class A manufacturing environment for API<br>●Environmental controls during open-container manipulations<br>●Retention sample testing<br>●Iterative viral testing scheme<br>●Post-production API testing and qualification	0
Amount of API per product dose	●Contamination of patient with large dose of viral pathogens	●Large dose of API per product dose	●Higher susceptibility toward infection and severe consequences	3	1	1	●Use of relatively small API quantity per product dose<br>●Use of sensitive detection methods for specified contaminants during testing<br>●Use of restrictive pathogen limits and thresholds	0
Process controls (donor, starting material, products)	●Failure in implemented process controls<br>●Inadequacy of process controls	●Systematic error in implemented controls<br>●Occasional error in implemented controls<br>●Apparition of new unspecified contaminants	●Liberation of contaminated API batch<br>●Infectious risk for the patient	3	1	2	●Iterative updates of process controls<br>●Iterative validation of process controls<br>●Use of orthogonal testing methods and experimental conditions (i.e., in vitro, in vivo, TEM)<br>●Redundant process controls<br>●Process controls implemented at appropriate stages of manufacture	0

Biosafety testing scheme	●Failure in implemented testing scheme<br>●Inadequacy of implemented testing scheme<br>●Emergence of pathogen absent in lower bank tiers (integrated, quiescent)	●Presence of latent or transient virus<br>●Presence of virus in undetectable quantities in low bank tiers	●Contamination of API batch<br>●Infectious risk for the patient	3	1	1	●Iterative updates of testing schemes<br>●Iterative and redundant testing steps<br>●Full quality and safety testing of WCBs (i.e., testing performed on manufacturing EOPCBs)	0

[1] Risk severity is classified as (0) acceptable, (1) tolerable, (2) undesirable, or (3) intolerable. [2] Risk likelihood is classified as (0) improbable, (1) possible, or (2) probable. [3] Risk level is classified as (0) low, (1) medium, or (2) high. [4] Post-mitigation risk level is classified as (0) low, (1) medium, or (2) high.

Table RA4. Specific risk analysis matrix established for assessment of the microbiological safety (i.e., excluding viruses) of primary FPC types, considering the cells as cryopreserved APIs for medicinal products. The microbiological safety (i.e., absence of bacteria, fungi, mycoplasma, mycobacteria, endotoxins) of the materials serving for manufacture of medicinal products is ensured by appropriate measures at the time of selection of starting, raw, and ancillary materials and testing thereof, before production, during production, and during post-production testing. API, active pharmaceutical ingredient; FPC, fibroblast progenitor cells; MCB, master cell bank; PCB, parental cell bank; QC, quality control; TEM, transmission electron microscopy; WCB, working cell bank.

Parameter	Pre-mitigation			Risk severity (0-3) [1]	Risk likelihood (0-2) [2]	Risk level (0-2) [3]	Mitigations	Post-mitigation risk level (0-2) [4]
	Risk	Cause	Effects					
Species of origin	●Risk of infection by zoonotic pathogens	●Inclusion of infected donor materials	●Zoonotic contamination of API and infection of patient	3	0	1	●Selection of human starting materials<br>●Thorough testing for pathogens with human tropism	0
Tissue of origin	●Use of contaminated starting materials	●Vertical transmission of pathogens<br>●Use of tissue type prone to contamination	●Contamination of API<br>●Infectious risk for the patient	3	1	1	●Selection of tissue with low probability of high contaminant yield<br>●Thorough qualification of donor<br>●Thorough qualification of donation	0
History of the donor, vertical transmission of pathogens	●Unknown contamination of donor<br>●Contamination of donor for unspecified pathogen	●Inadequate donor anamnesis<br>●Inadequate screening of donor	●Contamination of API<br>●Infectious risk for the patient	3	1	1	●Thorough donor anamnesis<br>●Thorough initial screening<br>●Thorough repeated screening<br>●Use of highly specific and sensitive screening methods	0

Contamination during API manufacturing	●Introduction of extraneous contaminant by reagents, equipment, material, personnel<br>●Emergence of latent or transient contaminant in culture	●Inadequate manufacturing process<br>●Inadequate control process<br>●Insufficient initial characterization of cell type<br>●Absence of purification regimen and terminal sterilization	●Contamination of API<br>●Infectious risk for the patient	3	1	2	●Qualification of source cell banks<br>●Class A manufacturing environment<br>●Selection of qualified and tested materials and reagents<br>●Environmental controls during open-container manipulations<br>●Minimization of open-container processes<br>●Minimization of contact processes<br>●Use of sterile single-use consumables<br>●Retention sample testing<br>●Post-production cell bank testing and qualification<br>●Post-production bulk product and final product testing and qualification	0
Infectivity or iatrogenesis of contaminated API	●Iatrogenic infection of patient<br>●Inadequate management of patient pathology	●Non-functional or potentially iatrogenic API	●Patient contamination<br>●No amelioration or worsening or patient health status	3	1	2	●Qualification of WCBs<br>●Class A manufacturing environment for API<br>●Environmental controls during open-container manipulations<br>●Retention sample testing<br>●Post-production API testing and qualification<br>●Post-production bulk product and final product testing and qualification	0

Amount of API per product dose	●Contamination of patient with large dose of pathogen	●Large dose of API per product dose	●Higher susceptibility toward infection and severe consequences	3	1	1	●Use of relatively small API quantity per product dose<br>●Use of sensitive detection methods for specified contaminants during testing<br>●Use of restrictive pathogen limits and thresholds	0
Process controls (donor, starting material, products)	●Failure in implemented process controls<br>●Inadequacy of process controls	●Systematic error in implemented controls<br>●Occasional error in implemented controls<br>●Apparition of new unspecified contaminants	●Liberation of contaminated API batch<br>●Infectious risk for the patient	3	1	2	●Iterative updates of process controls<br>●Iterative validation of process controls<br>●Use of orthogonal testing methods and experimental conditions (i.e., in vitro, in vivo, TEM)<br>●Redundant process controls<br>●Process controls implemented at appropriate stages of manufacture	0
Biosafety testing scheme	●Emergence of pathogen undetected in lower bank tiers	●Presence of pathogen in undetectable quantities in low bank tiers	●Contamination of API batch<br>●Infectious risk for the patient	3	1	1	●Iterative updates of testing schemes<br>●Iterative and redundant testing steps<br>●Full microbiological quality testing of WCBs	0

[1] Risk severity is classified as (0) acceptable, (1) tolerable, (2) undesirable, or (3) intolerable. [2] Risk likelihood is classified as (0) improbable, (1) possible, or (2) probable. [3] Risk level is classified as (0) low, (1) medium, or (2) high. [4] Post-mitigation risk level is classified as (0) low, (1) medium, or (2) high.
