# Supplementary material for: Optimized Manufacture of Lyophilized Dermal Fibroblasts for Next-Generation Off-the-Shelf Progenitor Biological Bandages in Topical Post-Burn Regenerative Medicine
Source: Biomedicines. 2021 Aug 23;9(8):1072. doi: 10.3390/biomedicines9081072 (PMC8394413; doi:10.3390/biomedicines9081072)
Supplement: Supplementary file 1 [file biomedicines-09-01072-s001.zip › Supplementary Documents/Supplementary Materials.pdf]

Supplementary Methods

1. Dermal FPC Sourcing and Parental Cell Bank Establishment

1.1. Primary Dermal FPC Sourcing

Two organ donations at 14 weeks of gestation (i.e., FS20/E16 and FE002 donations) served for the procurement of biological starting materials for establishment of the dermal FPC types (i.e., FS20/E16-Sk and FE002-SK2 cell types, respectively) used in the investigations presented herein. The main progenitor cell source used for this study was the FE002-SK2 primary cell type, a dermal progenitor fibroblast type established after processing of a cutaneous sample (i.e., 14 weeks of gestation) in 2009. FE002-SK2 primary progenitors were isolated from a voluntary organ donation following a validated protocol, approved by the State Ethics Committee (University Hospital of Lausanne – CHUV, Ethics Committee Protocol #62/07: "Development of fetal cell banks for tissue engineering", August 2007). The FE002 donation was registered under the Swiss FPC transplantation program and its biobank, complying with the laws and regulations within both programs (Figure S1).

1.2. Primary Dermal FPC Enzymatic Isolation

Anonymized and dissected tissue biopsies were processed by the institutional pathology department in the CHUV. Tissues were provided in individual and labelled containers, covered in sterile transport buffer (i.e., phosphate-buffered saline, PBS, Laboratorium Dr G. Bichsel AG, Interlaken, Switzerland). Tissues were thereafter processed immediately for cell culture initiation or were alternatively stored at refrigerated temperature (i.e., 2–8 °C) until further processing. Approximately 2 cm^2 of cutaneous tissue were isolated in culture plates and were rinsed thrice in conserved PBS (i.e., PBS containing 1% penicillin-streptomycin, Gibco™, Thermo Fisher Scientific, Waltham, MA, USA). The available tissue was then dissected into smaller fragments and placed in sterile centrifuge tubes (50 mL, Falcon®, Corning, Glendale, AZ, USA) before being covered with 5 mL of warmed (i.e., 37 °C) trypsin-EDTA (i.e., 0.25% trypsin and 0.1% ethylenediaminetetraacetic acid, Gibco™, USA) (Figure S4). After 15 min of incubation of the tubes at 37 °C, the enzymatic cell dissociation reaction was ended by addition of liquid cell culture medium (i.e., Dulbecco's Modified Eagle Medium, DMEM, Gibco™, USA; with 10% *v*/*v* clinical-grade fetal bovine serum, FBS, Invitrogen™, Thermo Fisher Scientific, Waltham, MA, USA). The cell dissociation tubes were then centrifuged at (230 ± 10) × *g* at ambient temperature for 15 min, before the resulting cellular materials were resuspended in pre-warmed initial cell culture medium. Cell suspension titers were determined, and the suspensions were used to seed non-coated culture Petri dishes (Falcon®, USA, Figure S4). The seeded culture vessels were then incubated at 37 °C, 5% CO_2, and 80% relative humidity. Thereafter, cell culture medium was exchanged every 48 h. Cultures were microscopically examined at each medium exchange procedure, for confirmation of cell adhesion, proliferation, adequate proliferative cellular morphology maintenance, and absence of observable extraneous agent contamination. Once optimal confluency was attained (i.e., 50% for Passage 0 cells, Figure S4), cell cultures were rinsed twice with PBS and were submitted to trypsinization (i.e., 0.05% trypsin-EDTA, Gibco™, USA). Following collection and pooling, cell suspension titers were determined. The suspensions were then centrifuged at (230 ± 10) × *g* at ambient temperature for 15 min, before cells were resuspended in pre-warmed complete growth medium (i.e., initial growth medium, with addition of L-glutamine, in order to reach a final concentration of 5.97 mM) and eventually distributed into one hundred T80 cell culture flasks (80 cm^2, Nunc™, USA) at a relative viable seeding density of 1.5×10^3 ± 200 cells/cm^2. Complete medium final volume was set at 15 mL/vessel, and cultures were incubated as described previously. Medium exchange procedures were performed every 48 h until 95% confluency was attained. After appropriate controls, Passage 1 cells were enzymatically harvested and resuspended in an ad hoc cryopreservation medium (i.e., complete medium 50% *v*/*v*; FBS 40% *v*/*v*; dimethyl sulfoxide 10% *v*/*v*, Sigma-Aldrich®, USA). Therein, final cell densities ranged from 10^6 to 10^7 viable cells/mL, and the cell suspensions were homogenously dispensed in labelled cryovials (1.8 mL cryovials, Nunc™, USA). Following appropriate controlled-rate freezing down to -80 °C, vial lots were transferred to liquid nitrogen (i.e., vapor phase) in a quarantine Dewar storage tank. Following batch release, the vials were further traceably stored in distinct secure storage tanks to mitigate risks of destruction. The described cryopreserved cellular material was defined as the enzymatically isolated FE002-SK2 parental cell bank (PCB, Passage 1).

1.3. Primary Dermal FPC Mechanical Isolation

Appropriate tissue biopsies were provided and rinsed, as described hereabove in the enzymatic cell isolation workflow. Then, fragments were transferred to culture Petri dishes (Falcon®, USA), which were previously scored in a checkerboard pattern (Figure S3). Biopsy fragments were further minced and attached along scored plastic regions (i.e., with 6–8 fragments/dish), favoring adherence and cell outgrowth. Approximately 2 mL of initial liquid growth

medium were carefully dispensed around the fragments. Culture vessels were incubated as described previously. The following day, 10 mL of cell culture medium were carefully added to each dish, before cultures were incubated again. Subsequently, the medium was exchanged every 48 h. Culture vessels were microscopically examined at each medium exchange procedure, for confirmation of cell emission from the tissue, adequate proliferative cellular morphology maintenance, and absence of observable extraneous agent contamination. Once optimal confluency was attained (i.e., 50% for Passage 0 cells, Figure S3), cell cultures were rinsed twice with PBS before trypsinization. Following cell collection and pooling, cell suspension titers were quantified, the suspensions were centrifuged at (230 ± 10) × *g* at ambient temperature for 15 min, and the cells were resuspended in pre-warmed complete growth medium. These cell suspensions were used to further seed one hundred T80 cell culture flasks (80 cm^2, Nunc™, USA) at a relative viable seeding density of $1.5 \times 10^3 \pm 200$ cells/cm^2. Complete medium final volume was 15 mL/vessel, and cultures were incubated as described hereabove. Cultures were maintained, harvested, and cells were cryopreserved as described previously for enzymatic cell isolation. The resulting cryopreserved cellular material was defined as the mechanically isolated FE002-SK2 parental cell bank (PCB, Passage 1).

2. Dermal FPC Manufacturing Optimization

2.1. Optimization of Cell Culture Parameters for Dermal FPCs

Various culture vessel surfaces were used for optimization of manufacturing yields. Therefore, dermal FPC WCB vials were used in recovery procedures and cell suspensions were seeded at various relative viable cell densities (i.e., 1.5×10^3, 3×10^3, 5×10^3, and 10^4 cells/cm^2) in culture flasks of various surfaces (i.e., 175 cm^2, 225 cm^2, and 500 cm^2, Nunc™, USA). Cultures were incubated as described hereabove for cell type establishment and were maintained appropriately with two medium exchange procedures per week. Various culture medium volumes (i.e., 0.05, 0.10, 0.15, 0.20, 0.25, and 0.30 mL/cm^2 of culture surface) and total expansion periods (i.e., 8, 10, 12, and 15 days) were used, before harvest and endpoint enumeration of expanded cells for comparison of respective manufacturing yields.

2.2. Optimization of Medium Supplement Source for Dermal FPCs

Dermal FPC WCB vials were used in recovery procedures for optimization of the medium supplementation source. Therefore, complete culture medium was prepared in adequate quantities using fetal bovine serum from Sigma-Aldrich® (i.e., three distinct lots), Invitrogen® (i.e., two distinct lots), and HyClone™ (i.e., one lot). In view of manufacturing yield maximization, cell culture Petri dishes (i.e., 78 cm^2) were seeded with 10^4 viable FE002-SK2 dermal FPCs at Passage 5. Cultures were maintained at 37 °C in humidified incubators under 5% CO_2. The culture medium (i.e., complete medium with the different FBS sources included for each condition) was exchanged twice per week and proliferative cellular morphology was assessed at that time by two senior operators. After fourteen days of culture, Giemsa staining was performed and endpoint grading (i.e., based on microscopic and macroscopic assessments of cultures) was performed (Figure S12).

2.3. Validation of Dissociation Reagent Equivalence for Dermal FPC Harvest

For validation of the switch from porcine trypsin to TrypLE™ dissociation reagent, WCB vials of two distinct dermal FPC types were used in recovery procedures and expanded to confluency in T75 culture flasks (75 cm^2, Nunc™, USA). For the assays, confluent cell cultures were harvested either using trypsin-EDTA (i.e., 0.05% trypsin) or TrypLE™ Express enzyme 1× (i.e., identical reagent volumes and dissociation reaction time of 6 min). Then, three T75 flasks per condition were seeded with 1.5×10^3 viable dermal FPCs (i.e., FE002-SK2 or FS20/E16-Sk cells) per cm^2 at Passage 5. Cultures were maintained at 37 °C in humidified incubators under 5% CO_2 and the culture medium (i.e., complete medium) was exchanged twice per week. At the defined timepoints (i.e., days 8, 10, and 12 after seeding), cells were harvested (i.e., using the appropriate specific dissociation reagent) and enumerated, for comparison of evolutive and endpoint cell yields (Figure S13).

2.4. Cell Surface Marker Analysis of Dermal FPCs

Dermal FPC WCB vials were used in recovery procedures for analysis of cell surface marker profiles by FACS analysis, for confirmation of cell population identity and purity. For characterization of cell surface markers, confluent FE002-SK2 fibroblast cultures at Passage 4 were rinsed thrice with PBS, were harvested using TrypLE™, and total cell counts were determined by hemocytometer enumeration. Cells were thereafter suspended in FACS buffer (i.e., 2% *v*/*v*

FBS in PBS) at a concentration of 10^6 cells/mL. Equivalent volumes of 100 μL of cell suspension were dispensed in 96-well round-bottom microtitration plates (Nunc™, USA). The plates were centrifuged at 400 × *g* for 5 min at ambient temperature. Thereafter, supernatants were removed, and cells were resuspended in 100 μL FACS buffer, before appropriate volumes of respective antibody stock solutions were added to each sample. Target cell surface markers included in the analysis comprised CD14, CD26, CD34, CD44, CD45, CD73, CD90, CD105, CD166, HLA-ABC, HLA-DP,DQ,DR, and D7-FIB. Accordingly, included antibodies were mouse IgG1 isotype CTRL FITC (ref. BD 554679, BD Biosciences, San Jose, CA, USA), mouse IgG1 anti-CD90 FITC (ref. BD 555595, BD Biosciences, USA), mouse IgG1 anti-CD105 FITC (ref. BD 561443, BD Biosciences, USA), mouse IgG1 anti-CD73 FITC (ref. BD 561254, BD Biosciences, USA), mouse IgG1 anti-CD45 FITC (ref. BD 555482, BD Biosciences, USA), mouse IgG1 anti-CD34 FITC (ref. BD 555821, BD Biosciences, USA), mouse IgG1 anti-HLA-ABC FITC (ref. BD 560965, BD Biosciences, USA), mouse IgG2a anti-isotype CTRL FITC (ref. BD 555573, BD Biosciences, USA), mouse IgG2a anti-CD14 FITC (ref. BD 555397, BD Biosciences, USA), mouse IgG2a anti-HLA-DP,DQ,DR FITC (ref. BD 555558, BD Biosciences, USA), mouse IgG2b anti-isotype CTRL FITC (ref. BD 555742, BD Biosciences, USA), mouse IgG2b anti-CD44 (ref. BD 555478, BD Biosciences, USA), mouse IgG2a anti-isotype CTRL PE (ref. BD 559319, BD Biosciences, USA), mouse IgG1 anti-CD26 PE (ref. BD 555437, BD Biosciences, USA), mouse IgG1 anti-CD166 PE (ref. BD 559263, BD Biosciences, USA), and mouse IgG2a anti-D7-FIB PE (ref. MA5-16642, Thermo Fisher Scientific, USA). The assay plates were protected from sunlight and were incubated appropriately for 30 min. Cells were thereafter resuspended in 400 μL FACS buffer and briefly vortexed before analysis. Samples were then processed on a BD Accuri™ C6 Plus flow cytometer (Beckman Coulter, Indianapolis, IN, USA) and data were processed with the FlowJo software (FlowJo, BD, Ashland, OR, USA).

2.5. Investigation of p63 Marker Expression by Dermal FPCs

Dermal FPC WCB vials were used in recovery procedures for additional confirmation of cell population identity and purity by analysis of p63 nuclear marker expression. Therefore, FE002-SK2 cells at Passage 6 were grown to confluency in optimized in vitro conditions. Positive control cells were HaCaT keratinocytes (CHUV Biobank). Confluent cultures were microscopically assessed for verification of normal cellular morphology. Cultures were then plated in 24-well microplates with approximately 42 × 10^3 cells per well and incubated as described previously. Four days later, the cultures were fixed with MeOH for 10 min, were rinsed once with PBS, and were stored at 4 °C until further processing. On the first day of immunostaining, cells were permeabilized with Triton® X-100 (A1388, AppliChem, Darmstadt, Germany) at 0.3% for 10 min and quickly washed once for 2 min with PBS-Tween 20 (A1389, AppliChem, Germany) at 0.025%. Quenching was then performed with BLOXALL® (Vector SP-6000, Vector Laboratories, Burlingame, USA) for 10 min, before a second wash for 2 min with PBS-Tween 20 at 0.025%. Then, the primary p63 antibody (i.e., Abcam, EPR5701, ab124762, 1:10^3, in PBS with 10% goat serum) was added and the plates were incubated overnight at 4 °C. Then, the cells were quickly washed once for 2 min with PBS-Tween 20 at 0.025%. The secondary antibody (i.e., in ImmPRESS™ anti-rabbit HRP, MP-7401, Vector Laboratories, USA) was then added and the plates were incubated at ambient temperature for 1 h. Then, the cells were quickly washed once for 2 min with PBS-Tween 20 at 0.025%. Revelation was then performed with ImmPACT™ DAB (SK-4105, Vector Laboratories, USA) and cells were rinsed twice for 5 min with water at ambient temperature. Cells were dehydrated with successive 30 s applications of the following solvents: EtOH 70%, EtOH 94%, EtOH 100%, EtOH 100%, xylol, and xylol. Finally, mounting was performed with Eukitt (Sigma-Aldrich®, USA) and cells were photographed appropriately, along with corresponding controls.

3. Optimization of Lyophilization Parameters for Stabilization of Dermal FPCs

3.1. Preparation of Cryoprotective/Lyoprotective Formulations for Dermal FPCs

In order to prepare samples for lyophilization, WCB vials of FE002-SK2 dermal FPCs were used in recovery procedures. Cells were seeded at 1.5 × 10^3 viable cells/cm^2 in T75 culture flasks and were culture-expanded using complete medium under 37 °C and 5% CO_2 until reaching 95% confluency. Cells were then enzymatically harvested, rinsed, enumerated, and resuspended in various sterile cryoprotective/lyoprotective solutions (Table A1). Final cell titers in the resulting suspensions were 10^6 to 10^7 total cells/mL. Corresponding placebos were prepared using bovine serum albumin (BSA) to replace the cellular components, based on equivalent total protein quantities. The resulting cell suspensions were then aseptically dispensed in 2R lyophilization vials (i.e., 2 mL ISO Schott type I glass vials, Schott, Mainz, Germany) with final filling volumes of 0.75 mL/vial. Vials were stoppered (i.e., 13 mm FluoroTec®

laminated Stoppers, Adelphi Healthcare Packaging, Haywards Heath, UK) at half position and either placed in LyoGuards® (Gore®, Newark, DE, USA) or in AdaptiQ® nests (AdaptiQ® Clip nest, 100 vials/nest, Schott, Germany) subsequently placed in lyophilization bags (Lyoprotect® single-use bag 420 x 460 and LPMU VS46 bag closure system, Teclen, Oberpframmern, Germany). The resulting packs were frozen in a defined process using ultra-low temperature freezers until further processing.

3.2. Dermal FPC Lyophilization Process Optimization

Technical optimization and semi-industrial production of dermal FPC lyophilizates were performed in collaboration with Prof. Louis Rey and subsequently with a specialized platform (Aérial, Illkirch, France). Differential thermal analysis (i.e., cooling and heating phases) and impedance measurements (i.e., Zsinφ, low frequency current, 1,000 Hz) on placebo formulations allowed to establish the optimal temperature, vacuum, and duration settings for the lyophilization cycle, with the objective of obtaining stable cakes and a residual relative humidity level under 2%. Structural analysis and data acquisition were performed using a cryo-microscope (Lyostat, Linkam, Tadworth Surrey, UK). Subsequently, for large-scale lyophilization processing, products were aseptically transferred to a cooled stainless-steel tray and were loaded for freeze-drying (LyoBeta 25, Telstar, Barcelona, Spain). The optimized processing phase parameters were determined and are presented in Table A3. After lyophilization, vials were fully stoppered and sealed using aluminum crimp-seals.

3.3. Quality Controls for Lyophilized Dermal FPCs

Residual humidity in the preparations was measured following the Karl Fischer method (Karl Fischer Compact, Mettler Toledo, Greifensee, Switzerland). Cake morphology was assessed by visual observation of the cake after the lyophilization process (i.e., application of parameters and criteria from Table 2). Sample homogeneity was assessed by particle size distribution analysis (Mastersizer 3000, Malvern Panalytical, Malvern, UK). Total protein contents (i.e., determined following manufacturer instructions with a BCA assay, QuantiPro™ BCA Assay Kit, Sigma-Aldrich®, USA) were compared to those of freeze-thawed (i.e., three successive cycles of liquid nitrogen to 37 °C waterbath transfers, 3 minutes per step) cell suspensions. Cell devitalization was assessed by microscopic observation using Trypan blue exclusion dye and was confirmed by cell recovery experiments (i.e., direct seeding of six-well microplates) using complete growth medium.

4. Characterization of Lyophilized Dermal FPCs

4.1. Proteomic Analysis of Lyophilized Dermal FPCs

A multiplex comparative analysis was performed with a specialized platform (Eve Technologies, Calgary, Alberta, Canada) on lyophilized FE002-SK2 cells and on freshly lysed FE002-SK2 cells (i.e., freeze-thawed). Both sample types were prepared using homogenous stock FE002-SK2 cell suspensions (i.e., PBS solvent, formula M, Table A1). For the lyophilization group samples, the cell suspension (i.e., 5×10^6 cells/mL) was distributed in vials and lyophilized as previously described, before being reconstituted in distilled water. Both sample types were sent frozen (-20 °C) for proteomic analysis. Noteworthy results are presented in Table S1.

4.2. Cell Surface Marker Analysis of Lyophilized Dermal FPCs

Selected cell surface markers (i.e., CD90, CD73, CD45, HLA-ABC, and CD44) were analyzed by FACS in the conditions and with the reagents previously described. Test-items were freshly harvested FE002-SK2 dermal FPCs, a sterilized version thereof (i.e., processed during 10 min at 121 °C for steam sterilization), and formulas F and I lyophilizates. Obtained plots were compared, in order to assess the effect of the processing method (i.e., sterilization or lyophilization) on the detection of the chosen cell surface markers.

4.3. Keratinocyte Proliferation Stimulation Potential of Lyophilized Dermal FPCs

Proliferation of primary keratinocytes was assessed after stimulation by FE002-SK2 cell lysates or lyophilized cell preparations (i.e., formula B). The cell lysate stock solution was standardized at 3.8 mg/mL, while the lyophilizate preparation was standardized at 2.8 mg/mL after BCA quantification of total proteins. Primary keratinocytes (MAR-54aF-K cell type, Passage 2, CHUV Biobank) were grown in CnT Prime Epithelial culture medium (N°3524, CELLnTEC, Bern, Switzerland) in T75 culture flasks (TPP, Switzerland), maintained as previously described. Once

optimal confluency was attained, cells were harvested, enumerated, and seeded in 6-well plates (i.e., triplicate conditions) at 3 × 10^3 viable cells/cm^2 in CnT Prime Epithelial culture medium (2 mL/well). Plates were incubated overnight at 37 °C under 5% CO_2. The medium was then discarded and replaced with CnT Prime Epithelial culture medium or CnT Prime Ker homeostasis medium (CELLnTEC, Switzerland) containing 0–15 µg/mL of cell lysates or lyophilized cell preparations, expressed in total protein concentration equivalents. Culture plates were incubated for 96 h before being photographically recorded and processed for cell counting (NanoEntek Counting chamber, N°DHC-N01, NanoEntek, Seoul, Korea).

4.4. Keratinocyte Migration Stimulation Potential of Lyophilized Dermal FPCs in a Scratch Assay

HaCaT cell migration stimulation potential of FE002-SK2 lyophilized cell preparations (i.e., formula M) was assessed. Keratinocytes were grown in specific medium in T75 flasks (TPP, Switzerland) as previously described. Once confluency was attained, cells were harvested, enumerated, and seeded in 24-well plates (N°3524 Costar®, Corning, USA) containing Ibidi inserts (N°80209, Ibidi, Gräfelfing, Germany). Cultures were maintained until confluent as described previously. Thereafter, the Ibidi inserts were removed, and the cell layers were washed with PBS. A volume of 400 µL of DMEM medium was added to each well, along with 100 µL of the test-item (i.e., reconstituted lyophilizates) in final concentrations (i.e., expressed in total protein concentration equivalents) of 0, 2.5, 5, and 10 µg/mL, respectively. Cultures were incubated again and regularly recorded. Pictures of the gaps were taken at the time of stimulation, 17 h later, and 26 h later, respectively. The reconstituted pictures were analyzed using ImageJ (NIH, USA) to quantitatively determine the remaining unpopulated surface of the assay wells.

4.5. Juvenile Fibroblast Proliferation Stimulation Potential of Lyophilized Dermal FPCs

Proliferation of primary juvenile fibroblasts was assessed after stimulation by FE002-SK2 cell lysates or lyophilized cell preparations (i.e., formula M). The lyophilizate preparation was standardized at 0.12 mg/mL after BCA quantification of total proteins. Primary juvenile fibroblasts (i.e., SNS/4a-F cell type, four-year-old donor, Passage 6, CHUV Biobank) were grown in complete culture medium in T75 flasks (TPP, Switzerland), maintained as previously described. Once optimal confluency was attained, cells were harvested, enumerated, and seeded in 24-well plates (i.e., in triplicate conditions) at 2 × 10^3 viable cells/cm^2 in culture medium containing 2% *v/v* FBS (i.e., 1 mL/well). The plates were incubated overnight at 37 °C under 5% CO_2. The medium was then discarded, and cells were washed once with PBS. Fresh culture medium (i.e., 500 µL) was then dispensed in the assay wells, and appropriate wells were fitted with Transwell® inserts (Greiner, Frickenhausen, Germany). Then, a volume of 100 µL of appropriate stimulation solution was added in each well, either in the culture medium or loaded in the Transwell® insert, to obtain final concentrations of lyophilizate (i.e., expressed in normalized total protein contents at the final dilution in the culture medium) of 0, 1, 5, and 15 µg/mL, respectively, for both assay conditions (i.e., with or without the insert). Assay plates were then incubated at 37 °C as described previously. After 72 h, the spent medium was exchanged appropriately for fresh medium, and the stimulation was repeated a second time, as described hereabove. Assay plates were then incubated at 37 °C as described previously. After 48 h, the plates were photographically recorded, the cells from each well were detached with TrypLE™ (Gibco™, USA), and were further processed for cell counting (NanoEntek Counting chamber, N°DHC-N01, NanoEntek, Seoul, Korea).

4.6. Adult Fibroblast Proliferation Stimulation Potential of Lyophilized Dermal FPCs

Proliferation of primary adult fibroblasts was assessed after stimulation by FE002-SK2 lyophilized cell preparations (i.e., formula I). The cell lyophilizate preparation was standardized at 1.7 mg/mL after BCA quantification of total proteins. The cell lysate preparation was standardized at 4.3 mg/mL after BCA quantification of total proteins. Primary adult fibroblasts (i.e., NP/CHFR-F cell type, seventy-six-year-old donor, Passage 6, CHUV Biobank) were grown in complete culture medium in T75 flasks (TPP, Switzerland), maintained as previously described. Once optimal confluency was attained, cells were harvested, enumerated, and seeded in 24-well plates (i.e., triplicate conditions) at 2 × 10^3 viable cells/cm^2 in culture medium containing 10% *v/v* FBS (i.e., 1 mL/well). Plates were incubated overnight at 37 °C under 5% CO_2. The medium was then discarded, and cells were washed once with PBS. Fresh culture medium (i.e., 500 µL) was then dispensed in the assay wells, and appropriate wells were fitted with Thincert® 0.4 µm inserts (Greiner, Germany). Then, a volume of 100 µL of appropriate stimulation solution was added in each well, either in the culture medium or loaded in the Thincert® insert, to obtain final concentrations of lysate or

lyophilizate (i.e., expressed in normalized total protein contents at the final dilution in the culture medium) of 0, 5, and 15 μg/mL, respectively, for both assay conditions (i.e., with or without the insert). Assay plates were then incubated at 37 °C as described previously. After 96 h, the cells from each well were detached with TrypLE™ (Gibco™, USA) and further processed for cell counting (NanoEntek Counting chamber, N°DHC-N01, NanoEntek, Seoul, Korea).

4.7. Effects of Dermal FPC Lyophilizates on Interleukins in a TNF Challenge Assay

A TNF-α challenge assay was used to assess the effects of lyophilized dermal FPC preparations on interleukins. Therefore, the FE002-SK2 lyophilizate preparations were standardized at 1.12 mg/mL (i.e., formula N), 2.80 mg/mL (i.e., formula B), and 1.70 mg/mL (i.e., formula I) after BCA quantification of total proteins. The FE002-SK2 lysate preparation was standardized at 4.30 mg/mL after BCA quantification of total proteins. Primary adult fibroblasts (i.e., NP/CHFR-F cell type, Passage 6) were grown in complete culture medium in T75 flasks (TPP, Switzerland), maintained as previously described. Once optimal confluency was attained, cells were harvested, enumerated, and seeded in 12-well plates (i.e., duplicate conditions) at 2.6×10^3 viable cells/cm^2 in culture medium containing 10% *v/v* FBS (i.e., 1 mL/well). Plates were incubated at 37 °C under 5% CO_2 as described previously. After 5 days of culture (i.e., cells at 90% confluency), assay wells were stimulated for 24 h with 10 ng/mL TNF-α (PeproTech 300-01A, PeproTech, London, UK), with and without cell-based preparations in various concentrations (i.e., expressed in normalized total protein contents at the final dilution in the culture medium) of 0, 1, 5, and 15 μg/mL, respectively. Assay plates were incubated at 37 °C as described previously. Then, conditioned media were harvested from specific wells and frozen at -20 °C until further analysis. Then, for quantification of IL-6 and IL-8 in collected supernatants, ELISA kits purchased from PeproTech (i.e., PeproTech 900-TM16 and PeproTech 900-T18, respectively) were used. Therefore, 96-well plates (high-binding ELISA plates, Greiner, Germany) were coated with a capture antibody (i.e., 100 μL per well of 0.5 μg/mL antibody, diluted in PBS). Plates were incubated overnight at ambient temperature. Wells were then washed thrice with washing buffer (i.e., PBS-Tween 20 at 0.05%, AppliChem, Darmstadt, Germany), before being filled with 200 μL of blocking buffer (i.e., PBS-BSA 1%). Plates were incubated for 1 h at ambient temperature. Wells were then washed thrice with washing buffer, before samples and standards (i.e., serial dilutions) were added. IL-6 and IL-8 standards were included in the ELISA kits. Manufacturer recommendations were followed for the assays. Supernatant test-items were used without being diluted. Prepared plates were then incubated for 2 h at ambient temperature, before being washed thrice with washing buffer. The detection antibody was then added to the assay wells (i.e., 100 μL of 100 ng/mL solution). Plates were then incubated for 2 h at ambient temperature, before being washed thrice with washing buffer. Streptavidin HRP (N°18-152, Merck Millipore, Burlington, VT, USA) was then added to the wells (i.e., 100 μL), and plates were then incubated for 30 min at ambient temperature and protected from sunlight. Wells were then washed thrice with washing buffer, and TMB (N°421101, BioLegend, San Diego, CA, USA) was added (i.e., 100 μL). The plates were then incubated at ambient temperature and protected from sunlight. The reaction was then stopped with 100 μL HCl 1 M, and the plates were analyzed for optic density determination (i.e., 450 nm and 560 nm wavelengths) on a Tecan microplate reader (Tecan Trading, Männedorf, Switzerland). Thereafter, the data processing and analysis was performed with the website (http://www.elisaanalysis.com/app), wherein a 4-parameter logistic regression curve was used.

Supplementary Figures

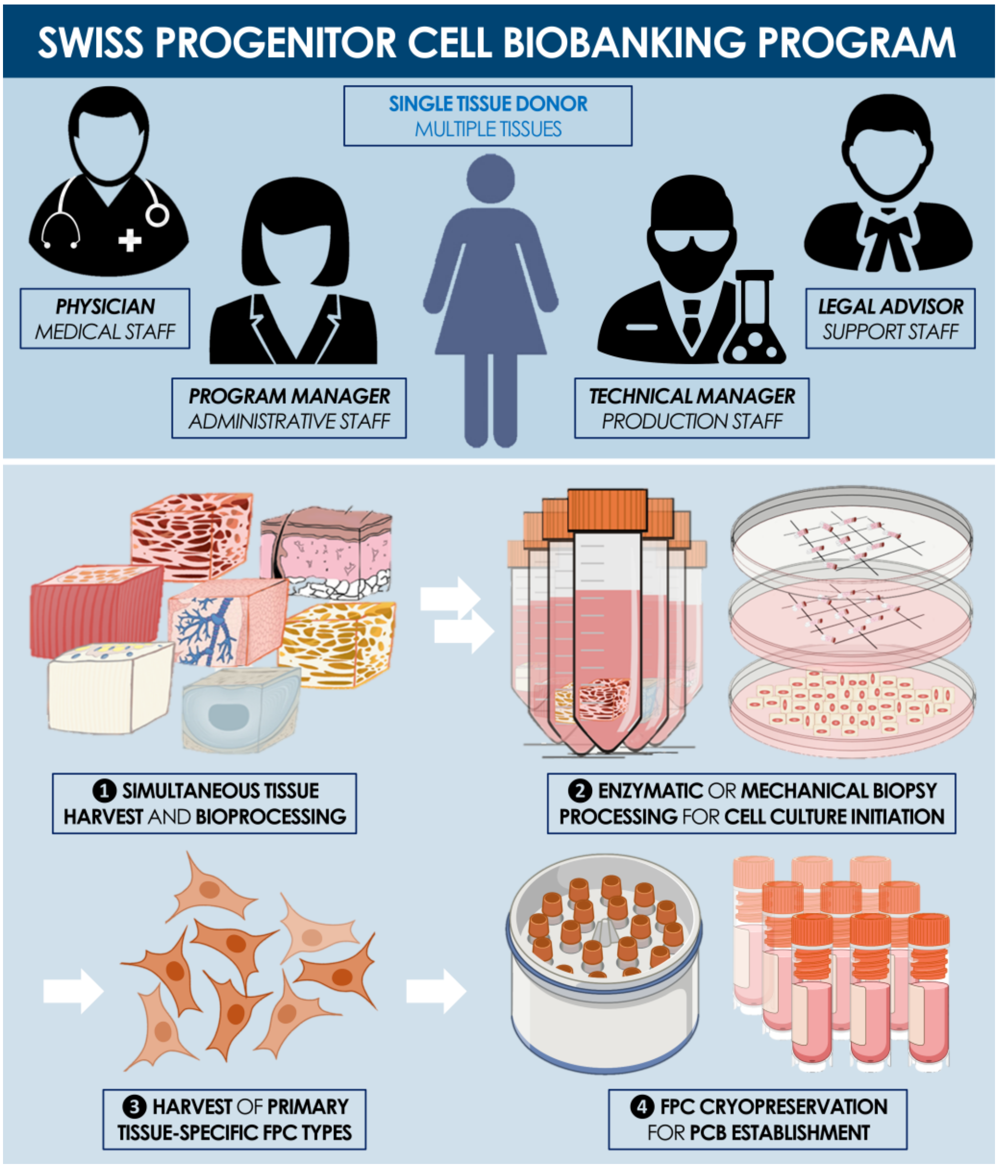

Figure S1. Schematic overview of the Swiss FPC transplantation program. Complementary interdisciplinary collaboration and pooling of competences enable the optimal design and implementation of such workflows for traceable yet anonymous procurement of specific tissue biopsies for bioengineering purposes. Designed compartmentalization between the different stakeholders ensures preservation of donor and donation anonymity. The program manager (i.e., biologist or pharmacist) establishes and coordinates the program, based on extensive experience in cell type establishment, technical specification optimization, and biobank administration. The legal advisor supports the program manager in the interpretation of applicable regulatory frameworks for transplantation medicine and designs the administrative portions of the program. The technical manager (i.e., biologist or senior laboratory technician with extensive experience in cell type establishment) oversees the tissue bioprocessing and subsequent cell banking steps. The medical doctor (i.e., experienced gynecologist) obtains the donation after screening and recruitment of a qualifying donor within the qualified tissue procurement organization. Overall, the defined program organigram enables anonymous traceability within the information and material flows. Iterative testing and validation steps ensure optimal microbiological safety and biological quality of all processed materials. Extensive pathology and serology testing reports are evaluated to confirm that each individual donation fulfills the requirements for inclusion in the program. Finally, established PCB lots are quarantined in liquid nitrogen tanks until the post-production testing results exclude seroconversion of the donor for selected target pathogens. The key value herein is the standardized approach for starting material procurement, bioprocessing thereof, and rapid establishment of consistent stocks of primary cultured FPCs to form parental cell banks. FPC, fibroblast progenitor cells; PCB, parental cell bank.

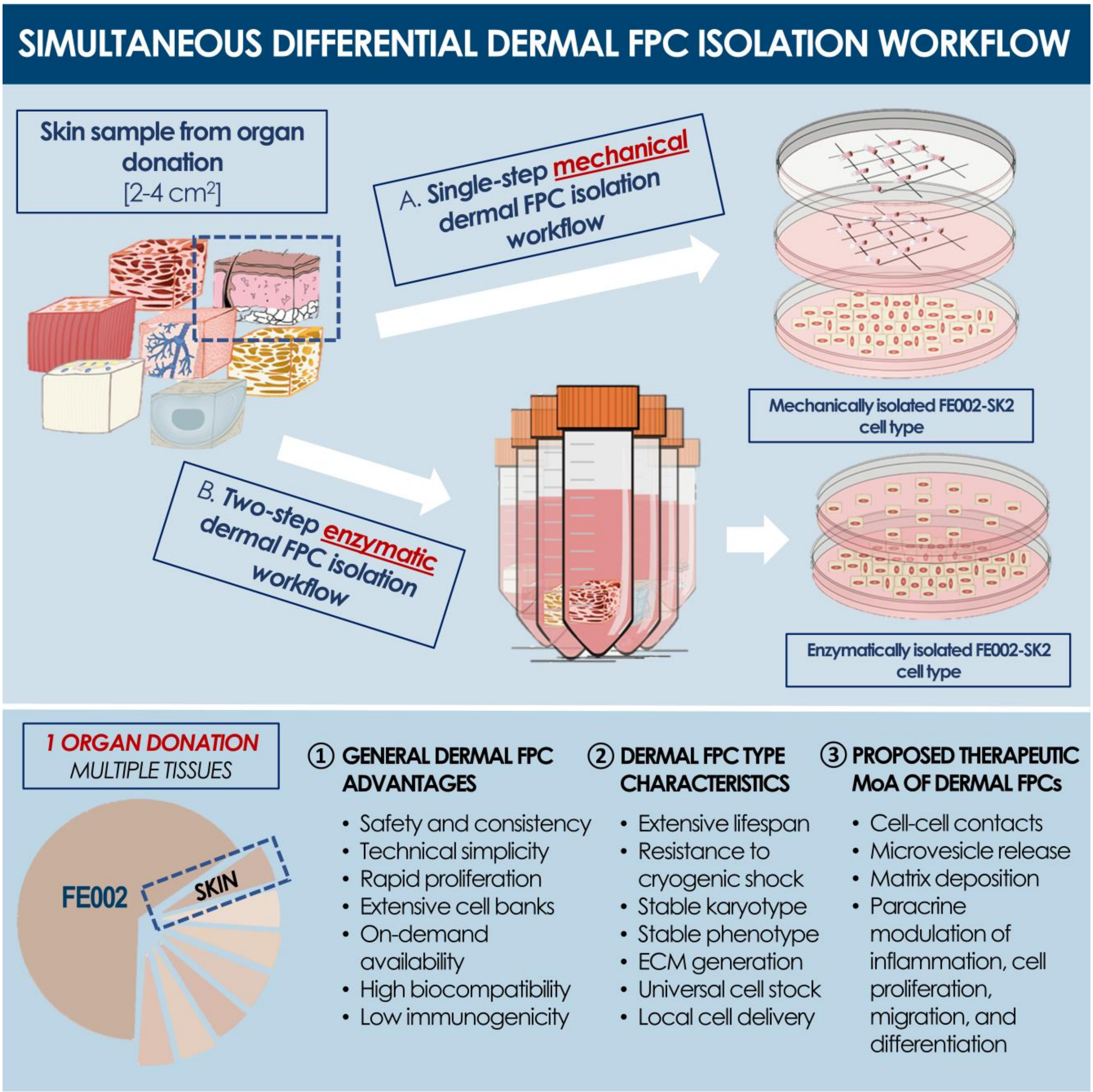

Figure S2. Schematic overview of the differential cell isolation workflow for dermal FPCs (e.g., FE002-SK2 cell types), along with general and specific advantages of using such cell types, as well as proposed therapeutic mechanisms of action (MoA) thereof. From a single organ donation (i.e., FE002 donation, 2009) included in a specifically devised FPC transplantation program, various tissue biopsies (e.g., bone, cartilage, intervertebral disc, lung, muscle, skin, tendon, etc.) were simultaneously differentially treated for primary FPC isolation and in vitro culture initiation, parallelly using both enzymatic (i.e., two-step) and non-enzymatic/mechanical (i.e., single-step) workflows. Optimized and standardized cell isolation procedures enable rapid and robust establishment of tissue-specific parental cell banks (PCB) to be valorized in diverse regenerative medicine applications. ECM, extra-cellular matrix; FPC, fibroblast progenitor cells; MoA, mechanism of action.

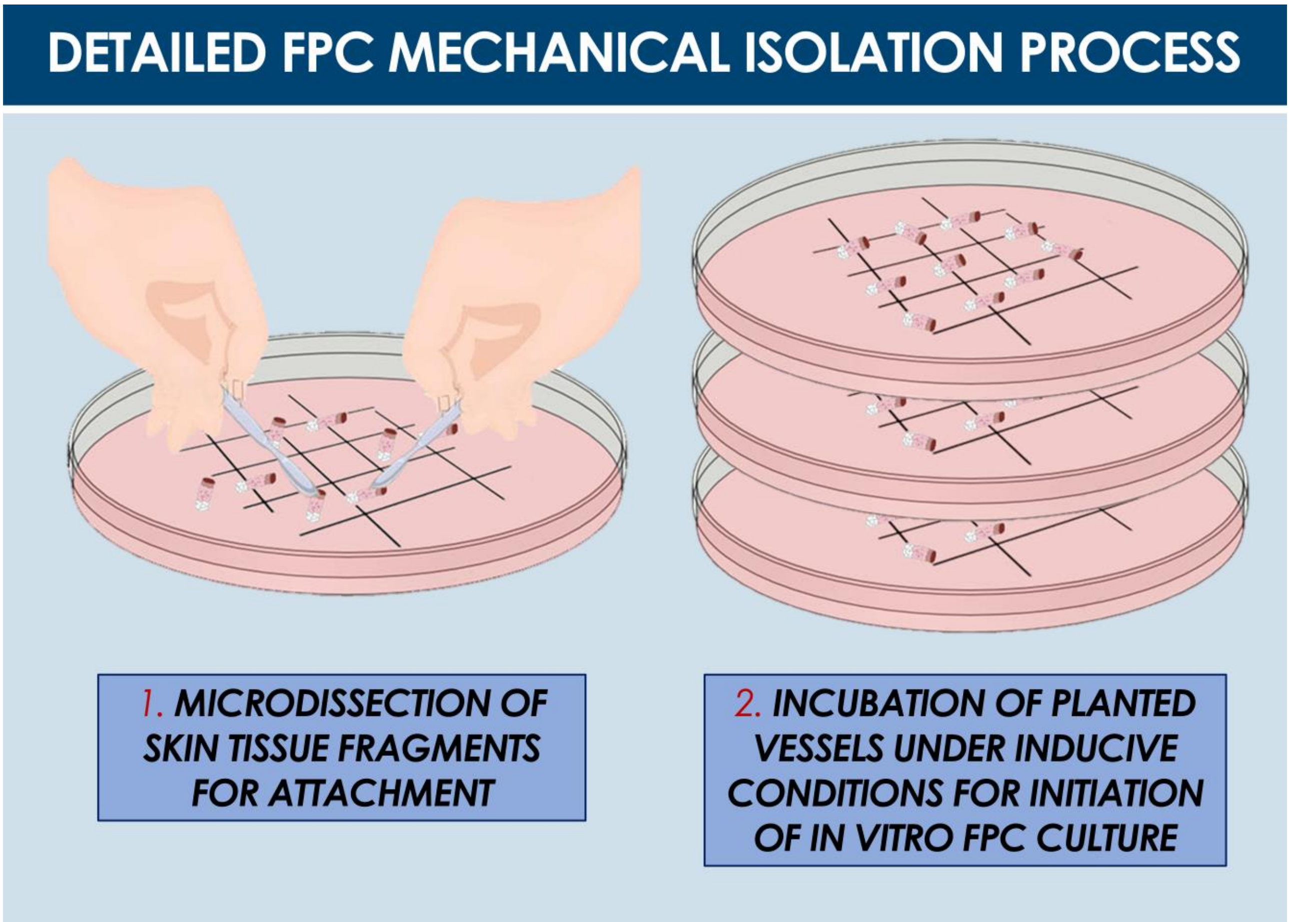

Figure S3. Schematic process for in vitro non-enzymatic/mechanical (i.e., single-step) isolation of dermal FPCs and primary culture initiation thereof. Briefly, non-coated tissue culture vessels are appropriately pre-conditioned, by means of deep scoring of the culture surfaces in a checkerboard pattern. Tissue fragments are then transferred in the vessels and are further minced. Following homogenous distribution over the scored surface, fragments are attached to the grooves. Vessels are subsequently incubated to appropriately favor adherent cell culture initiation. Such material processing and limited initial culture medium addition reduce the probability of tissue detachment and flotation during the first 24 h of culture. This method was described in the related patent "*Preparation of Parental Cell Bank from Foetal Tissue*", N°WO 2013/008174 A1, Applegate L.A.A., 2013. Specifically, following primary FPC isolation from the FE002 organ donation in 2009, the FE002-SK2 cell type (i.e., dermal progenitor fibroblasts) was deposited in the European Collection of Authenticated Cell Cultures (ECACC, Porton Down, UK, N°12070301-FE002-SK2, 2012) and at the Food Industry Research and Development Institute (FIRDI, Taiwan, N°BCRC 960460, 2012). FPC, fibroblast progenitor cells.

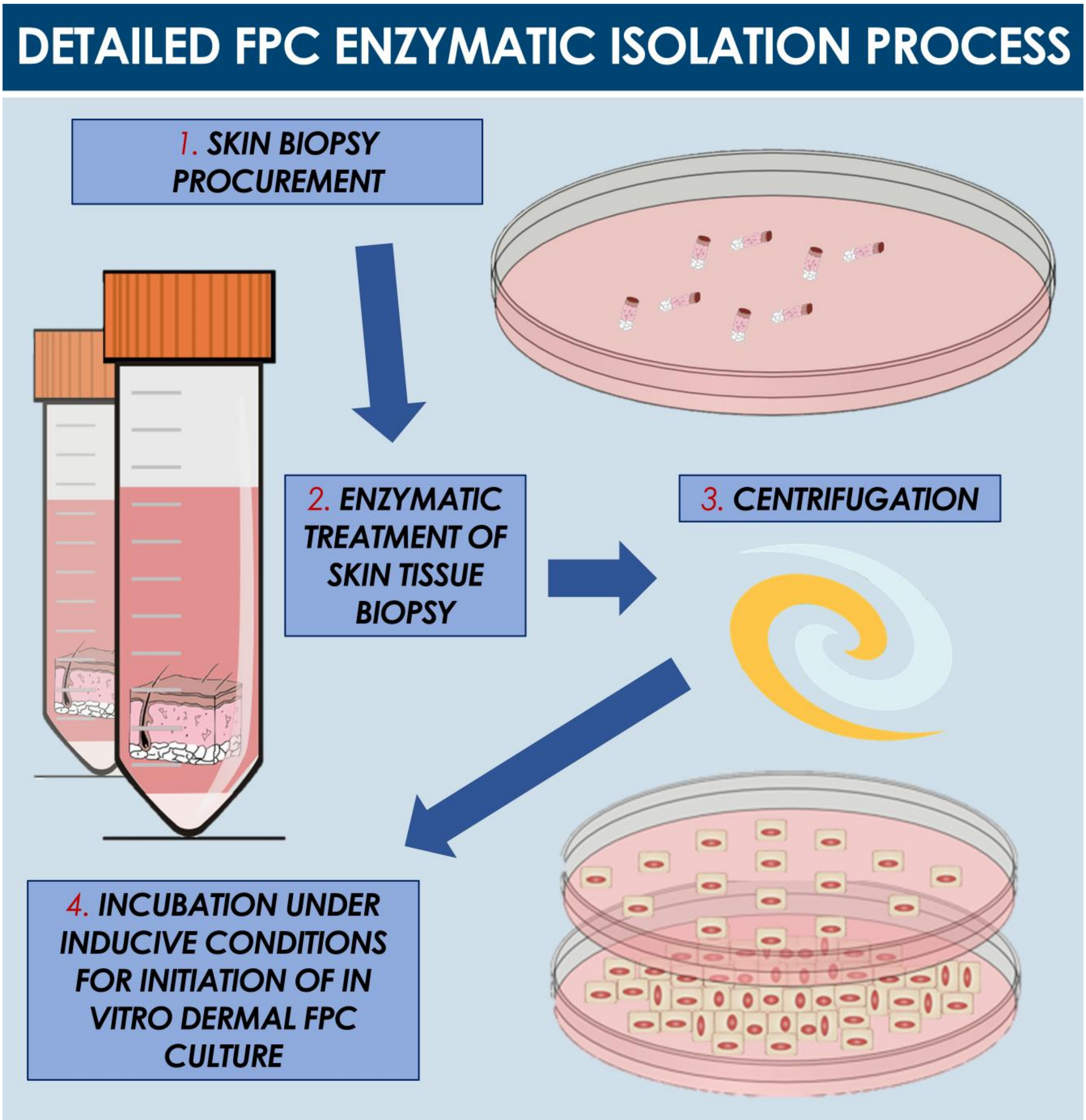

Figure S4. Schematic process for in vitro enzymatic (i.e., two-step) isolation of dermal FPCs and primary culture initiation thereof. Skin biopsies are processed into fragments and transferred into prepared tissue digestion tubes. After appropriate treatment (i.e., 10–20 min at 37 °C) with trypsin-EDTA (i.e., 0.25% trypsin and 0.1% ethylenediaminetetraacetic acid), dissociated FPCs are centrifuged, collected, resuspended in appropriate culture medium, and plated in culture dishes for adherent cell proliferation initiation. EDTA, ethylenediaminetetraacetic acid; FPC, fibroblast progenitor cells.

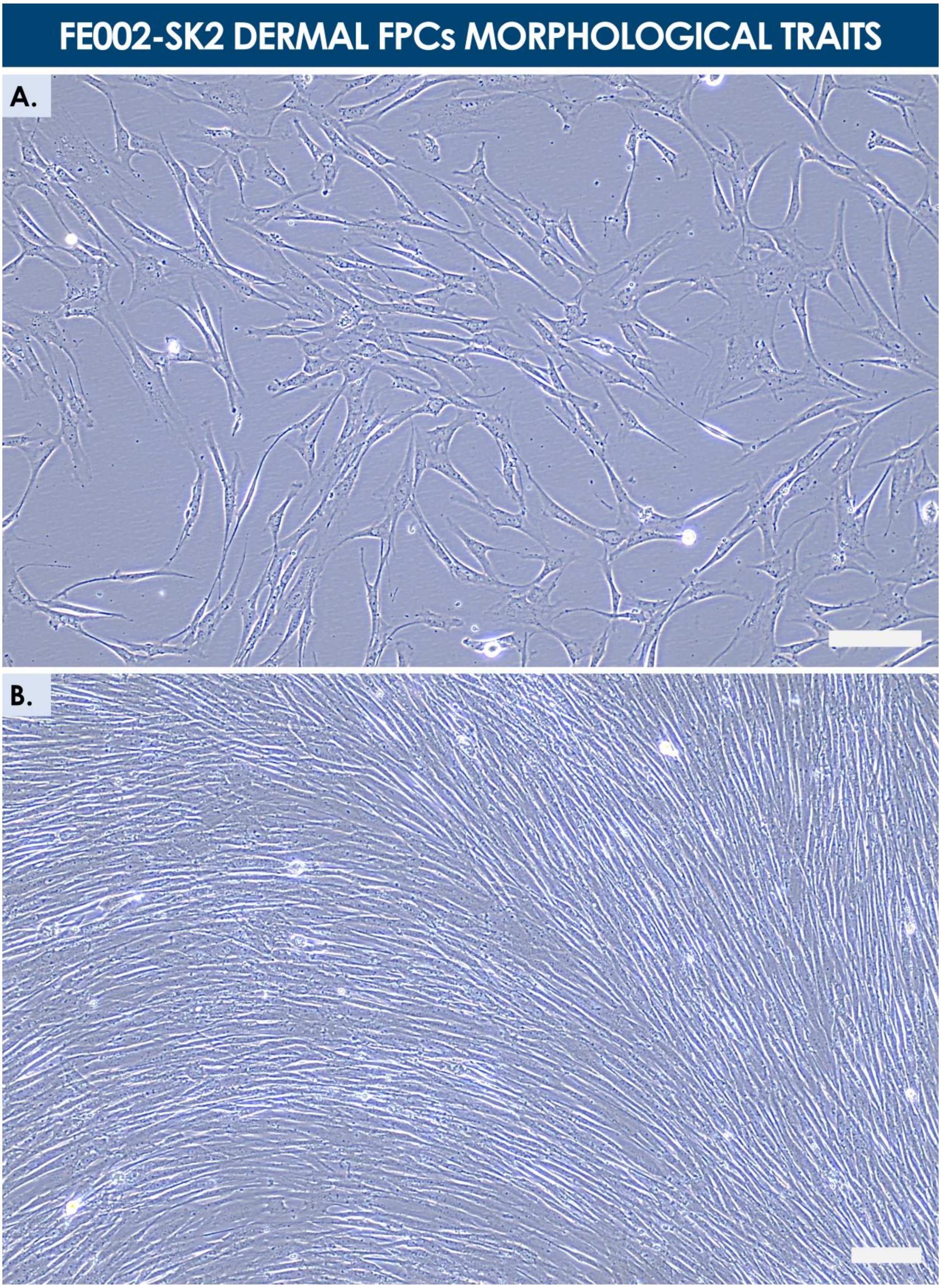

Figure S5. Morphological traits of cultured FE002-SK2 cells observed by contrast phase microscopy. (**A**) Photographic imaging of proliferating cells (i.e., Passage 6, after 7 days of culture). (**B**) Photographic imaging of confluent cells (i.e., Passage 6, after 14 days of culture). This cell type was deposited in the ECACC and at the FIRDI in 2012. Scale bars = 50 µm. ECACC, European Collection of Authenticated Cell Cultures; FIRDI, Food Industry Research and Development Institute; FPC, fibroblast progenitor cells.

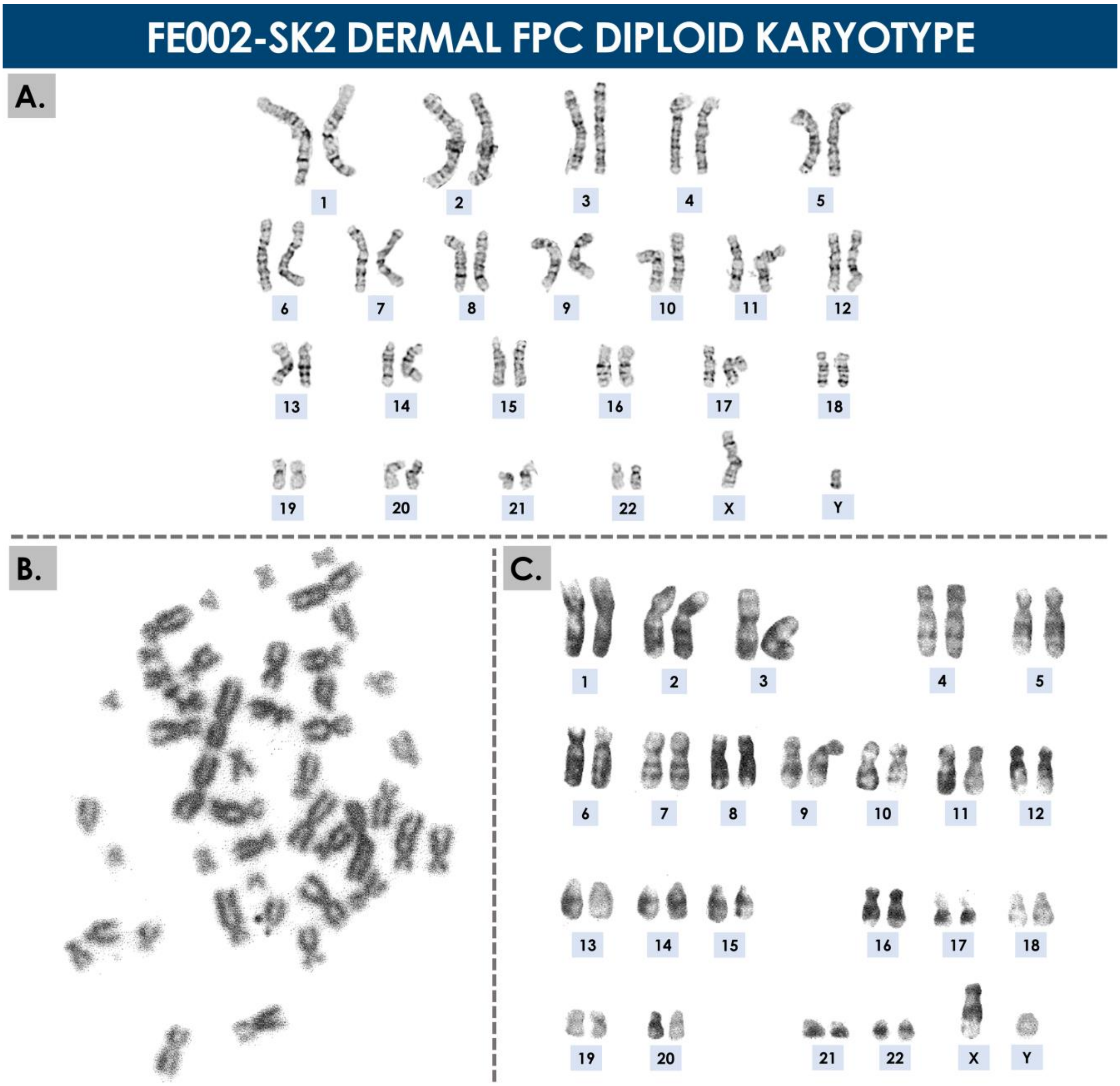

Figure S6. Diploid karyotype of the FE002-SK2 cell type. (**A**) Metaphase analysis of the FE002-SK2 fibroblast (i.e., Passage 3 cells) karyotype performed during donation qualification (Cytogenetics Laboratory, CHUV, Switzerland). (**B**) Karyotypic analysis (solid stained) of 50 metaphases of cellular materials (i.e., Passage 14 cells) derived from a FE002-SK2 EOPCB (BioReliance, UK) and stained with Giemsa stain. (**C**) Karyotypic analysis (G-banding) of 50 metaphases of cellular materials (i.e., Passage 14 cells) derived from a FE002-SK2 EOPCB (BioReliance, UK) and stained with Giemsa stain. Karyotypic analyses of metaphases indicated that the investigated cell type presented a strong modal chromosome number of 46. None of the scored metaphases had a count in the polyploid range. No evidence of chromosomal damage or chromosomal aberrations were observed. The investigated karyotype was consistent with a cell type of human origin. The number and appearance of chromosomes in all examined metaphases were consistent, thus confirming the absence of contamination by an extraneous cell line. CHUV, centre hospitalier universitaire Vaudois; EOPCB, end of production cell bank; FPC, fibroblast progenitor cells.

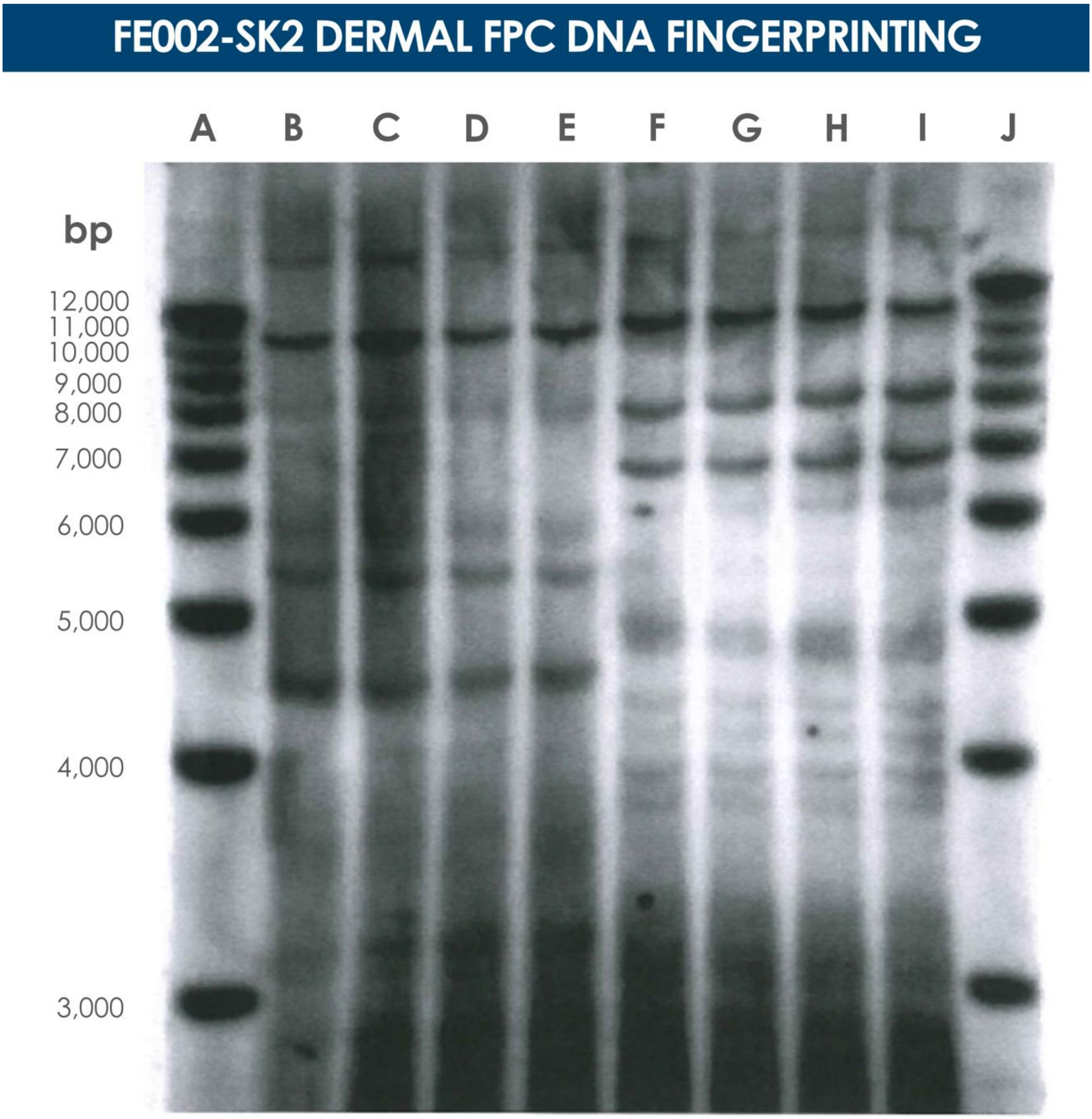

Figure S7. Hybridization of FE002-SK2 EOPCB-derived cell DNA (i.e., cells at Passage 13) using the multi-locus probe 33.15 for DNA fingerprinting and comparison to WCB-derived cell DNA (i.e., cells at Passage 5). In the presented assay, the EOPCB test-article generated DNA fingerprint profiles identical to the standard WCB DNA upon hybridization with the multi-locus probe 33.15. Specifically, generated DNA fingerprint profiles were identical for cells derived from the WCB and EOPCB, for which genetic materials were restricted with endonucleases Hae III and Hinf I, respectively, before being hybridized with the multi-locus probe 33.15. Results confirmed that the comparatively investigated cell samples were genetically identical at the repetitive mini-satellite loci detected by the multi-locus probe. No evidence of cell type cross-contamination was observed. (**A**) Lane loaded with 1 kbp DNA ladder. (**B**,**C**) Lanes loaded with 5 µg DNA from WCB-derived cells, digested with endonuclease Hae III. (**D**,**E**) Lanes loaded with 5 µg DNA from EOPCB-derived cells, digested with endonuclease Hae III. (**F**,**G**) Lanes loaded with 5 µg DNA from WCB-derived cells, digested with endonuclease Hinf I. (**H**,**I**) Lanes loaded with 5 µg DNA from EOPCB-derived cells, digested with endonuclease Hinf I. (**J**) Lane loaded with 1 kbp DNA ladder. Bp, base pairs; DNA, deoxyribonucleic acid; EOPCB, end of production cell bank; FPC, fibroblast progenitor cells; WCB, working cell bank.

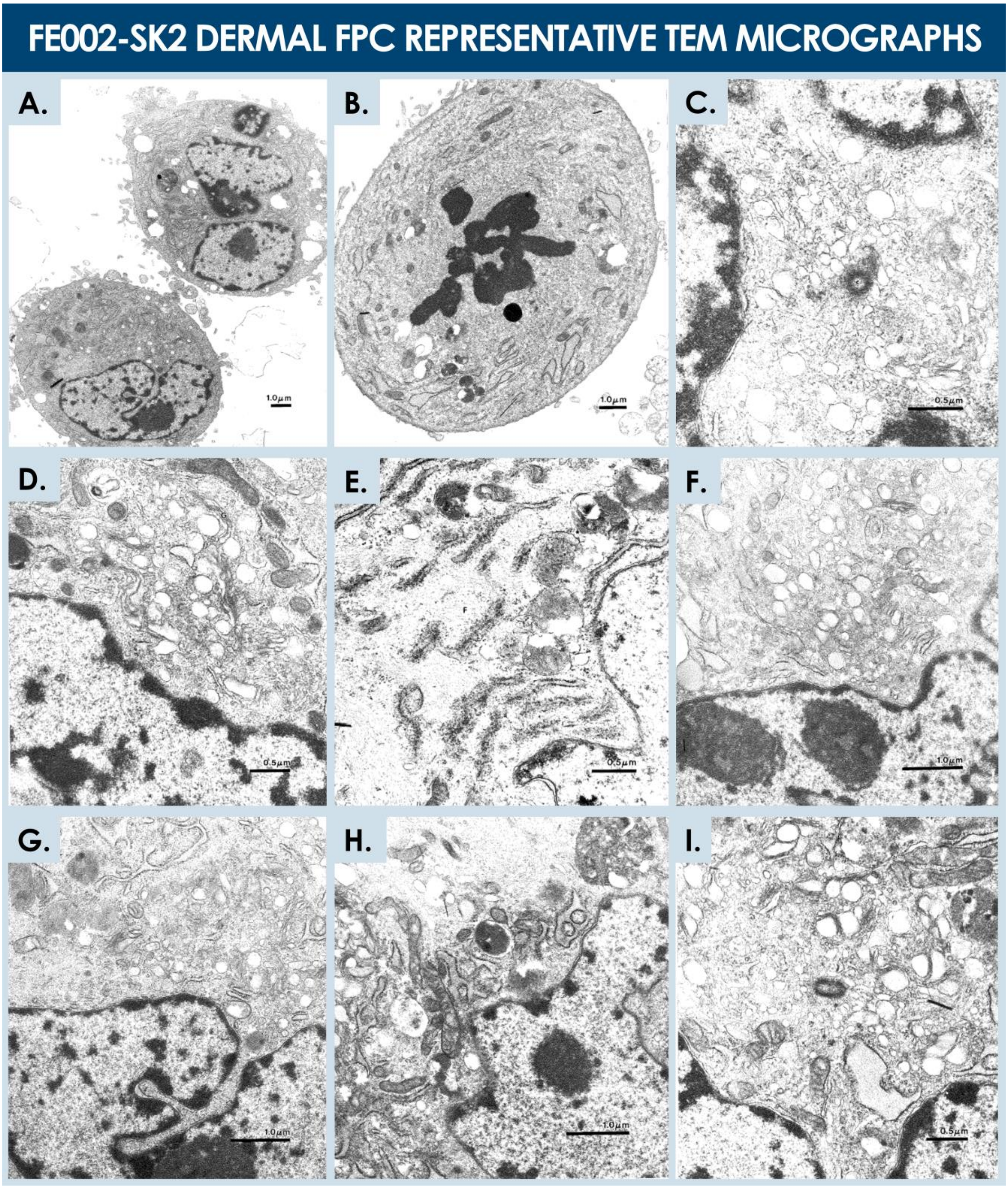

Figure S8. Representative TEM micrographs of cell sections from a FE002-SK2 WCB. (**A**) Cell areas illustrating the main characteristics of the cells present in the sample. (**B**) A mitotic cell. (**C**) Area of a cell with a centriole, Golgi bodies, vacuoles, mitochondria, and part of the nucleus. (**D**) Area of a cell with Golgi bodies, mitochondria, and part of the nucleus. (**E**) Area of a cell with fibrils, vacuoles, and part of the nucleus. (**F**) Area of a cell with mitochondria, Golgi bodies, vacuoles, and part of the nucleus. (**G**) Area of a cell with Golgi bodies, mitochondria, and part of the nucleus. (**H**) Area of a cell with fibrils, vacuoles, mitochondria, and part of the nucleus. (**I**) Area of a cell with a centriole, vacuoles, Golgi bodies, and part of the nucleus. Scale bars = 0.5 µm or 1.0 µm. FPC, fibroblast progenitor cells; TEM, transmission electron microscopy; WCB, working cell bank.

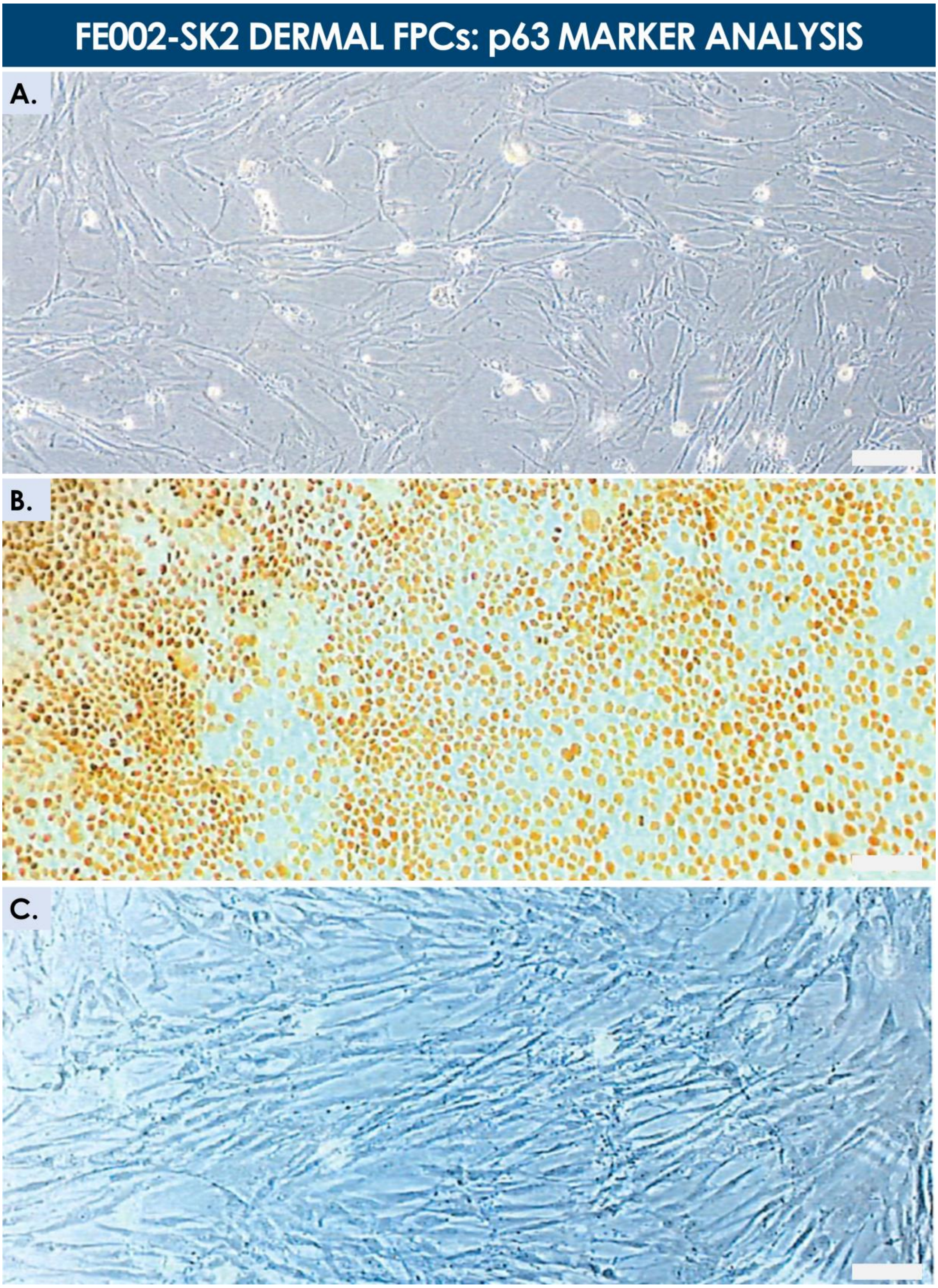

Figure S9. Analysis of the p63 marker expression by FE002-SK2 cells (e.g., Passage 6 cells). (**A**) Photographic imaging of FE002-SK2 cell cultures in standard conditions. (**B**) HaCaT cell positive controls for p63 marker expression, stained. (**C**) FE002-SK2 sample analysis for p63 marker expression, stained. All investigated FE002-SK2 test-samples were found to be negative for p63 marker expression, confirming the absence of population contamination by epidermal cells. Scale bars = 50 µm. FPC, fibroblast progenitor cells.

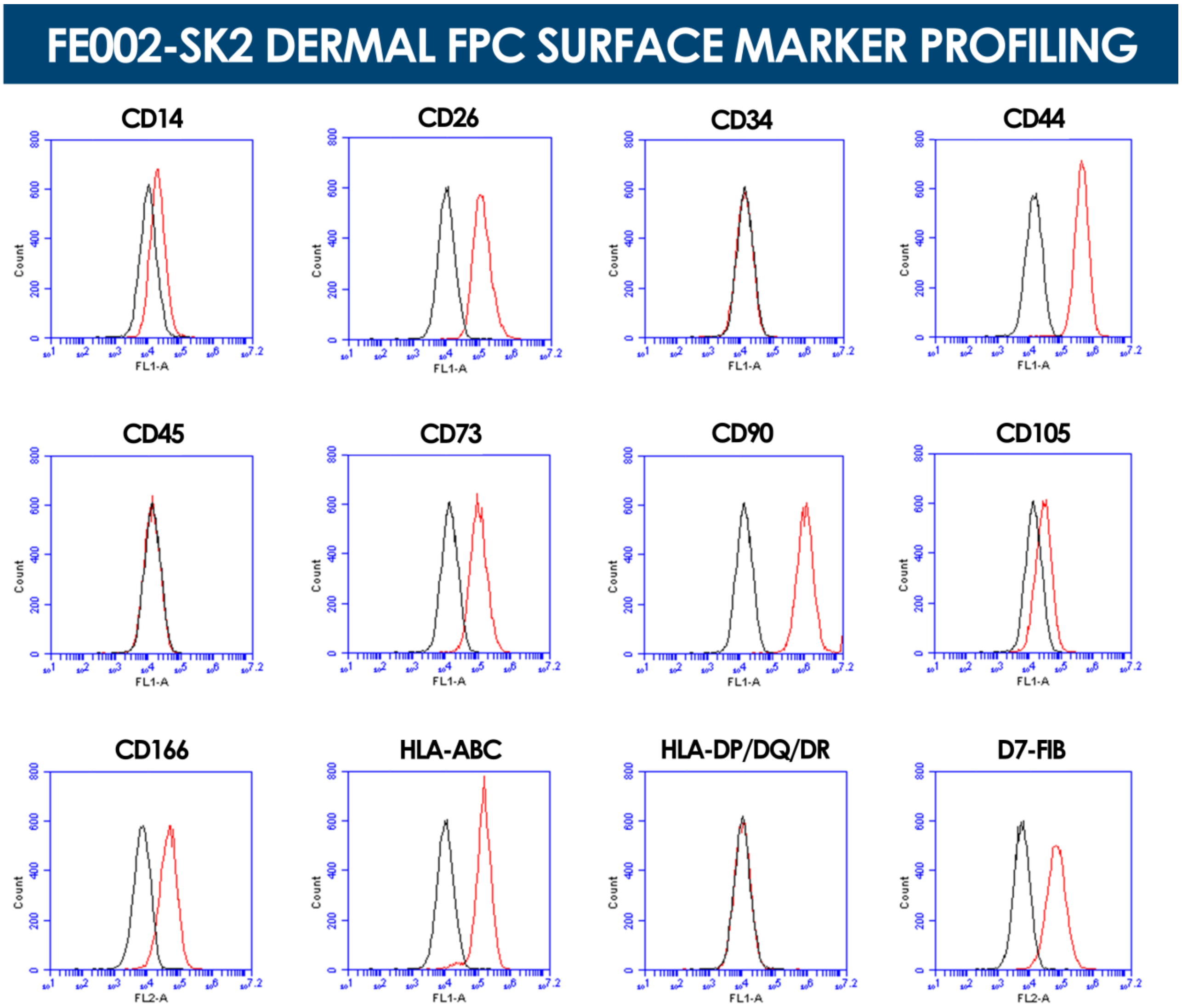

Figure S10. Surface marker profiling results of dermal FPCs (e.g., FE002-SK2 cells at Passage 4) using FACS analysis, following EP monograph 2.7.24. "*Flow cytometry*". Control curves are presented in black. Results showed a consistent panel of surface markers for FE002-SK2 dermal FPCs (i.e., CD34$^-$, CD45$^-$, HLA-DP,DQ,DR$^-$, CD14$^+$, CD26$^+$, CD44$^+$, CD73$^+$, CD90$^+$, CD105$^+$, CD166$^+$, HLA-A,B,C$^+$, and D7-FIB$^+$). Notably, investigated cells were found to be positive for general MSC markers (i.e., CD44, CD73, CD90, and CD105), whereas the adipogenic, osteogenic, and chondrogenic differentiation potentials of the same dermal FPCs were found to be highly restricted or inexistant, as compared to those of ASCs (data not shown). ASC, adipose stem cells; CD, cluster of differentiation; EP, European pharmacopoeia; FACS, fluorescence-activated cell sorting; FPC, fibroblast progenitor cells; HLA, human leukocyte antigen; MSC, mesenchymal stem cells.

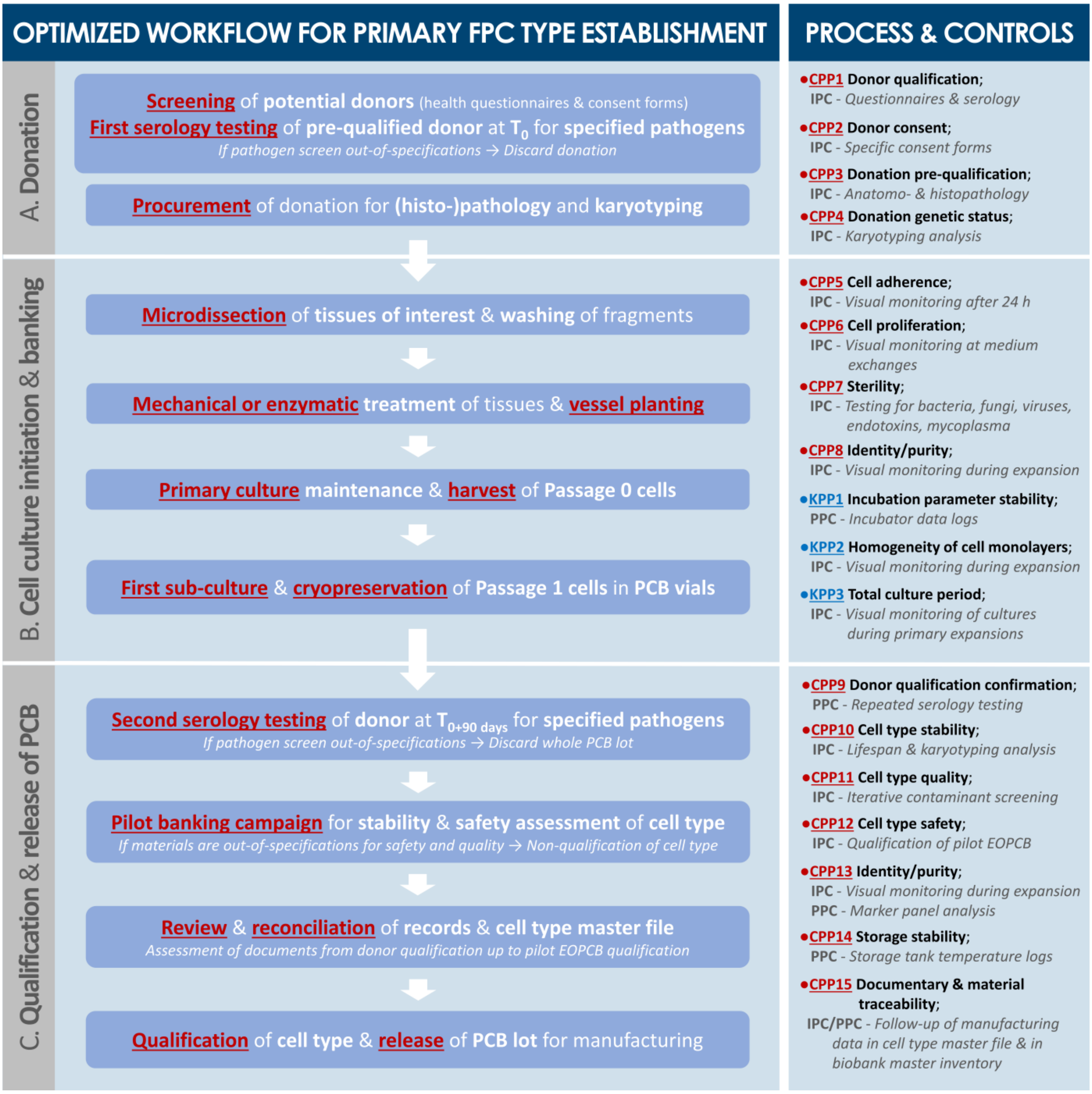

Figure S11. Optimized schematic workflow for biological starting material sourcing and primary dermal FPC type establishment. (**A**) Critical aspects in the donation procurement phase are the donor qualification and consent obtention, in view of mitigating potential ethical and legal exposure, as well as the donation qualification, in view of ensuring maximal safety and quality of starting biological materials. (**B**) Critical aspects in the cell isolation and culture initiation phase comprise the correct adherence or migration of primary FPCs following tissue processing, as well as confirmed proliferation for obtention of consistent preliminary cultures of the cell type of interest. (**C**) For sound exploitation of specific established primary FPC types, appropriate qualification thereof is essential for assurance of material safety, quality, and sustainability. Overall, highest attention must be paid to methodological and documentary aspects governing tissue sourcing and primary cell type establishment, as varied subsequent therapeutic applications require exhaustive starting material traceability. CPPs were defined as parameters exerting a critical effect on the quality of the final manufactured cell batch (i.e., FPC PCB). KPPs were defined as parameters exerting a key effect on the quality of the final manufactured cell batch (i.e., FPC PCB). CPPs, KPPs, IPCs, and PPCs presented herein are further defined and detailed in the Supplementary Document "Process Parameters". CPP, critical process parameter; EOPCB, end of production cell bank; FPC, fibroblast progenitor cells; IPC, in-process control; KPP, key process parameter; PCB, parental cell bank; PPC, post-process control.

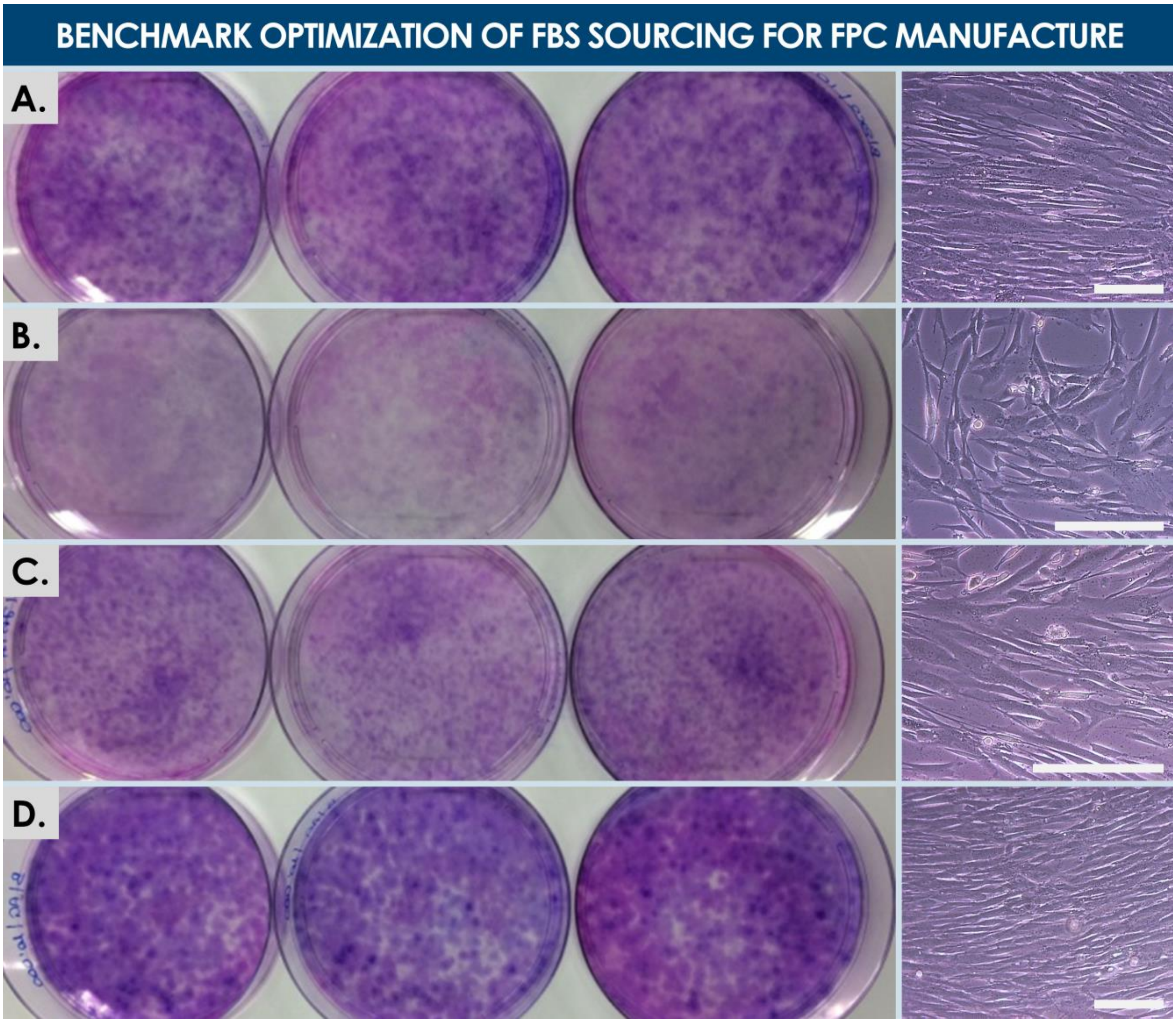

Figure S12. Optimization of FBS sourcing for dermal FPC manufacturing. In view of manufacturing yield maximization, several sources (i.e., manufacturers and lot numbers) of FBS were benchmarked. Presented assay dishes (i.e., 78 cm^2) were seeded with 10^4 viable FE002-SK2 dermal FPCs at Passage 5. Cultures were maintained at 37 °C in humidified incubators under 5% CO_2. The culture medium (i.e., complete medium with the different FBS sources included for each condition) was exchanged twice per week and proliferative cellular morphology was iteratively assessed at that time by two senior operators. After fourteen days of culture, Giemsa staining was performed on all cultures, and endpoint grading (i.e., based on microscopic and macroscopic assessments of cultures) was performed for each FBS source. (**A**) Triplicate conditions for Sigma® FBS lot A. (**B**) Triplicate conditions for Sigma® FBS lot B. (**C**) Triplicate conditions for HyClone™ FBS lot A. (**D**) Triplicate conditions for Sigma® FBS lot C. Scale bars = 50 µm. FBS, fetal bovine serum; FPC, fibroblast progenitor cells.

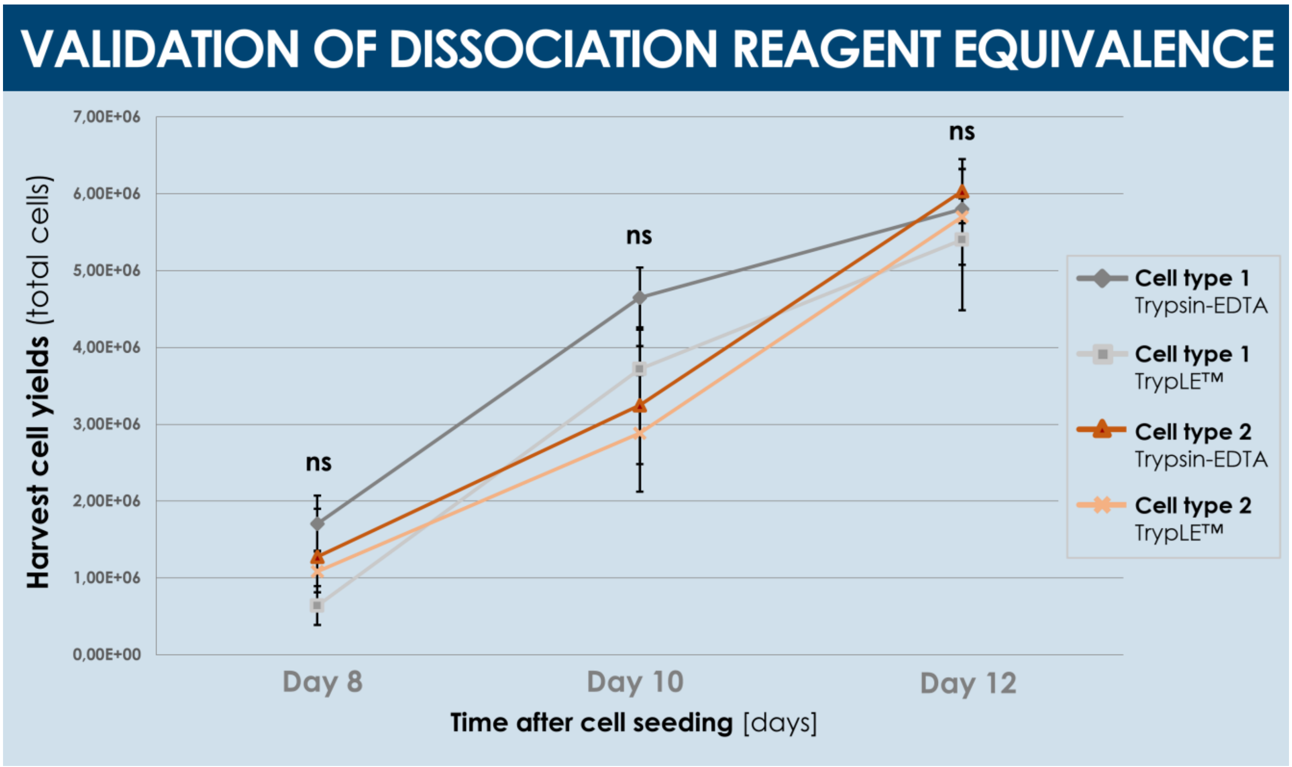

Figure S13. Experimental validation of the equivalence between trypsin-EDTA and TrypLE™ Express enzyme, based on cell recovery, proliferation, and enumeration after harvest. For the assay, confluent cultures of dermal FPCs were harvested either using trypsin-EDTA (i.e., 0.05% trypsin) or TrypLE™ Express enzyme 1× (i.e., identical reagent volumes and dissociation reaction time of 6 min). Then, three T75 flasks per condition were seeded with 1.5×10^3 viable dermal FPCs (i.e., FE002-SK2 or FS20/E16-Sk cells) per cm^2 at Passage 5. Cultures were maintained at 37 °C in humidified incubators under 5% CO_2 and the culture medium (i.e., complete medium) was exchanged twice per week. At the defined timepoints (i.e., days 8, 10, and 12 after seeding), cells were harvested and enumerated, for comparison of evolutive and endpoint cell yields. Results were not determined to be statistically different (i.e., when comparing obtained results for each respective cell type). EDTA, ethylenediaminetetraacetic acid; FPC, fibroblast progenitor cells; ns, non-significative.

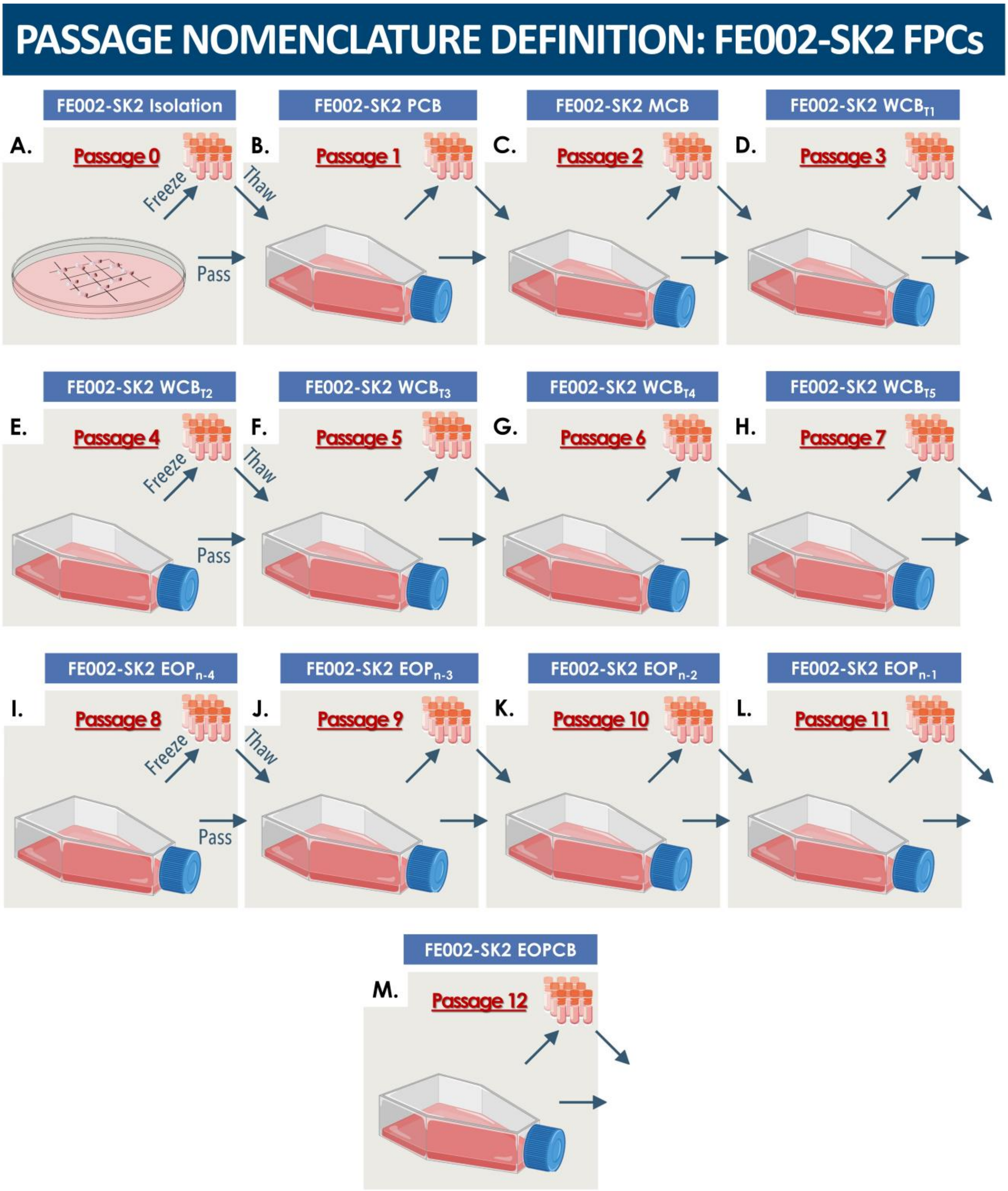

Figure S14. Schematic overview of mechanical dermal FPC culture initiation (e.g., FE002-SK2 cell type) and definition of in vitro passage (P) numbers in relation with standardized subsequent cell banking tiers. Therein, cells in PCB vials were defined as belonging to P1 in frozen state, becoming P2 upon thawing and subsequent culture initiation. (**A**) Initiation of FE002-SK2 adherent culture following non-enzymatic cell isolation, preliminary cell expansion, and cell harvest for preservation or passage of P0 cells. (**B**) Initiation or expansion of cells followed by endpoint harvest for cell preservation (i.e., FE002-SK2 PCB) or passage of P1 cells. (**C**–**M**) Serial initiation, expansion, harvest, and preservation procedures for cells through the various cell bank tiers (i.e., MCB, WCB, EOPCB). EOPCB, end of production cell bank; FPC, fibroblast progenitor cells; MCB, master cell bank; PCB, parental cell bank; WCB, working cell bank.

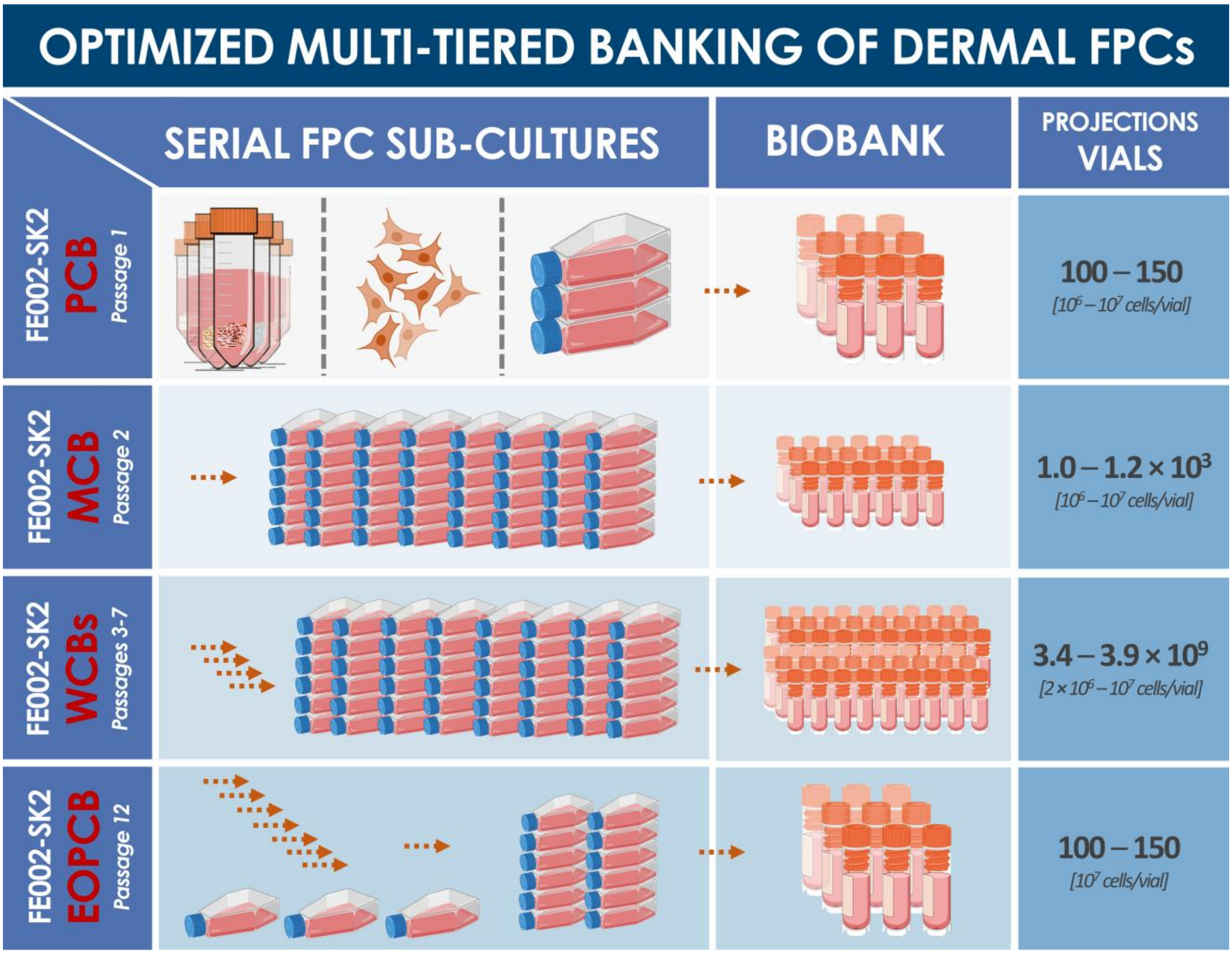

Figure S15. Optimized multi-tiered biobanking workflow for sustainable exploitation of primary dermal FPC types (e.g., enzymatically isolated FE002-SK2 cell type). Therein, with conditioning in vials of 10^6 to 10^7 cells, the theoretical numbers of obtainable PCB, MCB, and WCB vials are presented in the last column on the right. Such values were obtained by applying a specifically devised model of FPC banking, based on the optimized technical specifications presented in this study. Model data were repeatedly experimentally confirmed in GMP production for MCBs and WCBs. It is to note that passage levels are preferred to population doubling levels for characterization of in vitro cell age. This is made possible by the consistent use of constant cell seeding densities, culture periods, and harvest cell densities. EOPCB, end of production cell bank; FPC, fibroblast progenitor cells; GMP, good manufacturing practices; MCB, master cell bank; PCB, parental cell bank; WCB, working cell bank.

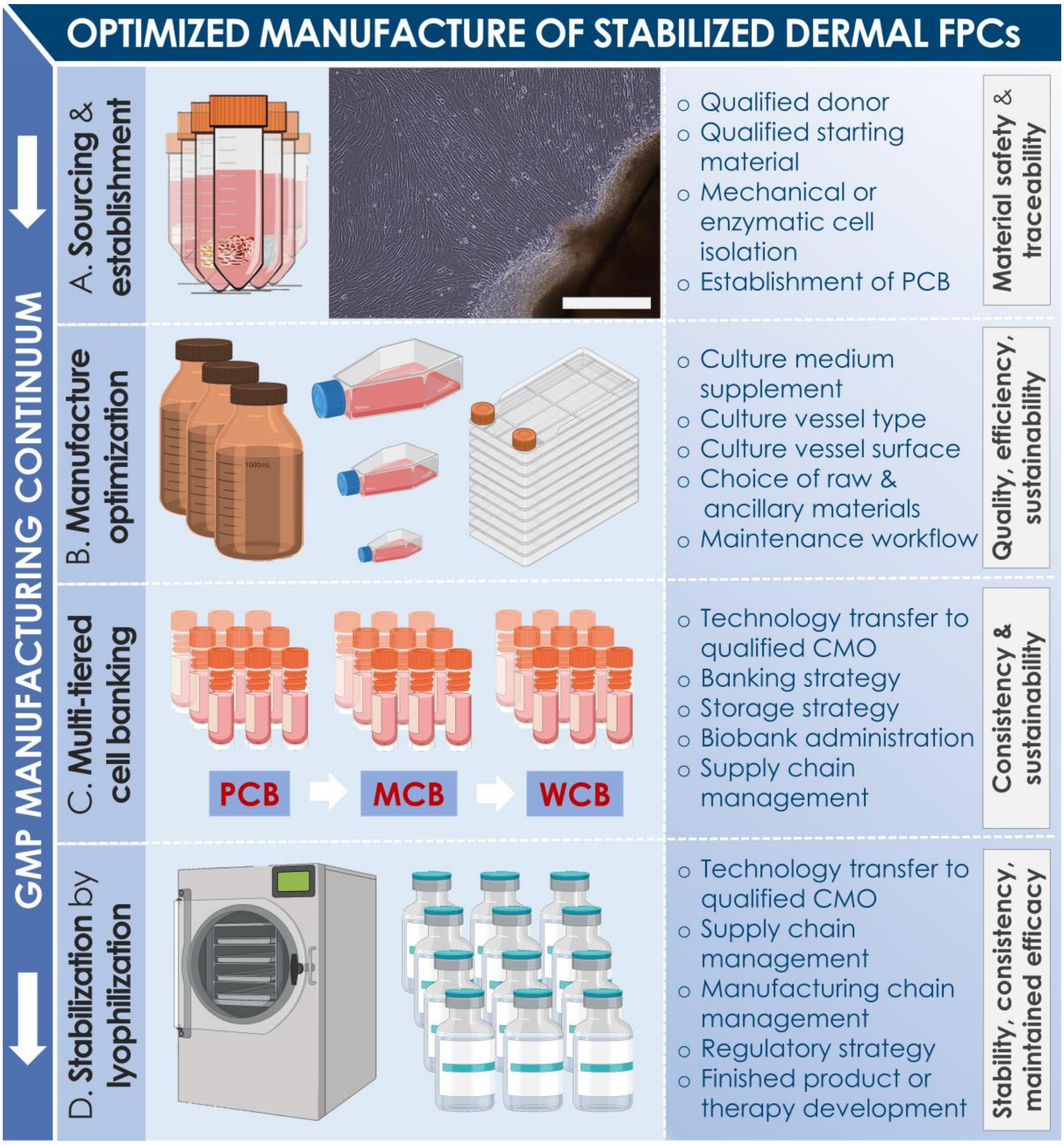

Figure S16. Overview of optimized manufacturing workflow of stabilized dermal FPCs. Critical or key process-related elements or phases are mentioned for each step as bullet points, along with the overall target parameters to be optimized at each step (i.e., in grey boxes). (**A**) Biological starting material sourcing, cell isolation, and establishment of dermal FPC PCBs. Critical parameters comprise the qualification of the tissue donor and of the tissue donation, in order to guarantee safety and traceability during further manufacturing phases and eventual clinical application. The central panel contains a photographic representation of dermal FPCs (i.e., FE002-SK2 cells, Passage 0) emitting from the tissue biopsy during mechanical culture initiation. Scale bar = 200 µm. (**B**) Manufacture process optimization for definition of standardized technical specifications to be subsequently used in large-scale production of cell lots. Appropriate benchmarking and selection of consumables and materials (e.g., FBS source, culture vessel model and surface) are paramount for quality, efficiency, and sustainability of defined cell type exploitation. (**C**) Multi-tiered cell banking of dermal FPCs, with sequential derivation of MCB and WCB production lots from original PCBs. For ensurance of consistency and sustainability, exhaustive and optimized technical specifications enable fluid transposition of manufacturing of the cellular active substance to GMP CMOs. (**D**) Optimization of dermal FPC stability using lyophilization processes enable enhanced consistency, maintained efficacy, and use of highly simplified logistical workflows. Stabilized cells or cell derivatives may thereafter constitute components of finished products to be appropriately registered. CMO, contract manufacturing organization; FBS, fetal bovine serum; FPC, fibroblast progenitor cells; GMP, good manufacturing practices; MCB, master cell bank; PCB, parental cell bank; WCB, working cell bank.

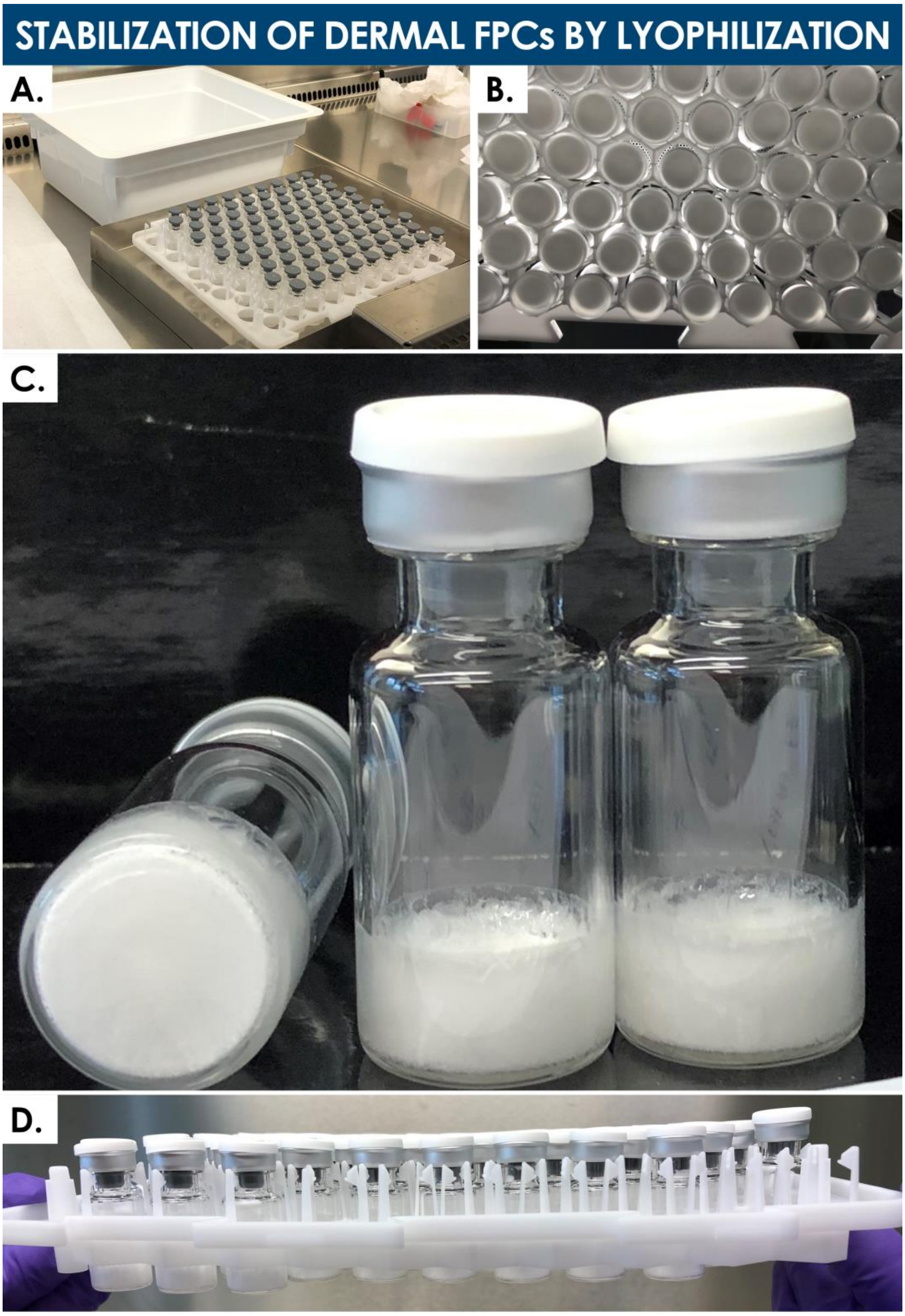

Figure S17. Photographic overview of lyophilization processing of a batch of FE002-SK2 FPCs in a Schott AdaptiQ® Nest (i.e., 2R vials). The cryo- and lyo-protective mix used in the formulation of the batch was formula I (Table A1). (**A**) Photographic imaging of the Nest, with vials half-stoppered after filling. (**B**) Photographic imaging of the inferior side of the vials after lyophilization, presenting homogenous and low-porosity cakes. (**C**) Photographic imaging (i.e., close-up) of lyophilized formulas recorded during product characterization. It is to note that the upper part of the cakes did not suffer from collapse, but the presence of liquid residues on the vial walls at the time of freezing results in a fill line higher than the effective initial fill volume. (**D**) Photographic imaging of a batch of lyophilizates, stoppered and capped. FPC, fibroblast progenitor cells.

Supplementary Tables

Table S1. Proteomic data for the qualitative and quantitative composition determination of lyophilized dermal FPC (i.e., FE002-SK2 cell type) preparations, as compared to harvested and lysed cells. Detected protein concentrations are presented in μg/mL. FPC, fibroblast progenitor cells.

**Protein ID**	**Protein content in cell lysates** (μg/mL)	**Protein content in cell lyophilizates** (μg/mL)	**Relative increase factor in detected protein concentration** (log_{10})
sVEGFR-2	10.74	8,289.71	2.89
sIL-1RII	5.61	1,364.38	2.39
sVEGFR-3	10.41	2,378.33	2.36
sVEGFR-1	50.37	6,020.90	2.08
HB-EGF	2.24	161.62	1.86
MMP-13	100.39	3,116.19	1.49
TGF-β1	6.55	179.48	1.44
FGF-2	256.82	6,149.67	1.38
Fractalkine	3.50	83.76	1.38
sgp130	1,139.91	24,569.00	1.33
Leptin	16.43	336.33	1.31
GRO-a	13.70	268.24	1.29
Follistatin	395.05	6,664.81	1.23
MMP-3	122.60	1,672.90	1.13
MCP-1	62.22	511.19	0.91
IL-8	11.08	77.05	0.84
TIMP-2	8,044.15	52,486.76	0.81

HGF	290.64	1,510.57	0.72
VEGF-A	463.74	1,881.57	0.61
sEGFR	2,006.26	7,808.76	0.59
TIMP-1	4,599.24	14,807.10	0.51
sTNFRI	676.86	1,705.00	0.40
MMP-1	1,496.74	3,364.57	0.35
MMP-2	3,224.36	2,551.05	-0.10
